# Mammea B/BA Isolated From the Seeds of *Mammea americana* L. (Calophyllaceae) is a Potent Inhibitor of Methicillin-Resistant *Staphylococcus aureus*

**DOI:** 10.3389/fphar.2022.826404

**Published:** 2022-03-11

**Authors:** Yina Pájaro-González, Andrés F. Oliveros-Díaz, Julián Cabrera-Barraza, Eduardo Fernández-Daza, Niradiz Reyes, Oscar A. Montes-Guevara, Daneiva Caro-Fuentes, Luis Franco-Ospina, Wiston Quiñones- Fletcher, Cassandra L. Quave, Fredyc Díaz-Castillo

**Affiliations:** ^1^ Laboratory of Phytochemical and Pharmacological Researches, School of Pharmaceutical Sciences, University of Cartagena, Cartagena, Colombia; ^2^ Research Group in Healthcare Pharmacy and Pharmacology, Faculty of Chemistry and Pharmacy, University of Atlántico, Barranquilla, Colombia; ^3^ Research Group Genetic and Molecular Biology, School of Medicine, University of Cartagena, Cartagena, Colombia; ^4^ Biological Evaluation of Promising Substances Group, Faculty of Pharmaceutical Sciences, University of Cartagena, Cartagena, Colombia; ^5^ Organic Chemistry of Natural Products, University of Antioquia, Medellín, Colombia; ^6^ Center for the Study of Human Health and Department of Dermatology, Emory University, Atlanta, GA, United States

**Keywords:** bacteriostatic, anti-staphylococcal, mamey, coumarins, bioguided-fractionation

## Abstract

*Staphylococcus aureus* remains a pathogen of high concern in public health programs worldwide due to antibiotic resistance and emergence of highly virulent strains. Many phytochemicals have demonstrated activity against *S. aureus* and other Gram-positive bacteria, but the minimum inhibitory concentration (MIC) values comparable to commonly used antibiotics are needed. In the present study, bio-guided fractionation of the ethanol extract of seeds of *Mammea americana* L. (Calophyllaceae) throughout the antibacterial activity, against *S. aureus* strains that are sensitive and resistant to methicillin, led to the isolation of four coumarins identified as mammea B/BA, mammea B/BC, mammea A/AA cyclo D and mammea A/AA cyclo F, and a mixture of mammea B/BA cyclo F plus mammea B/BD cyclo F. The extract inhibited the growth of *S. aureus* with MIC values of 2–4 μg/ml and Mammea B/BA (MaBBA) presented MIC values in a range between 0.5 and 1.0 μg/ml in six methicillin-sensitive strains and eight methicillin-resistant strains evaluated. We consider MaBBA the most potent of all mammea coumarins reported to date, according to the literature review carried out at the time of writing of this article. Toxicity assessment *in vivo* against the nematode *Caenorhabditis elegans* and *in vitro* against human fibroblasts of the extract and the compound MaBBA indicated that both had low toxicity.

## Introduction

Due to the multiple advantages of natural products (Atanasov et al., 2021), modern medicine still depends on them and their molecules for the development of new drugs, being the area of the anti-infectives and anti-cancers where there is a more marked influence (Newman and Cragg, 2020; Luo et al., 2021). Natural products remain the main source of antibacterials (Singh, 2012; Harvey et al., 2015; Wiese and Imhoff, 2019). Metabolites isolated from microorganisms have proven to be the most potent antibiotics used by man; however, several bioactive molecules have been isolated and characterized from plants with great potential to fight all types of infections (Chassagne et al., 2021; Porras et al., 2021). Some plant metabolites have matched the potency of antibiotics isolated from microorganisms; for example, hyperforin, an acylfloroglucinol isolated from *Hypericum perforatum* L. (St. John’s Wort) showed a minimum inhibitory concentration (MIC) of 1 μg/ml against methicillin-resistant *S. aureus* (Reichling et al., 2002). One of the main drawbacks of the secondary metabolites isolated from plants has been their limited activity against Gram-negative bacteria, compared with their antibacterial activity against Gram-positive bacteria, since the former have reduced permeability, this being a common drawback with molecules from other natural sources (Singh, 2012). Although it is preferable to have broad-spectrum antibiotics (Cheung et al., 2021), the isolation of active molecules against Gram-positive bacteria remains a laudable alternative because one of the microorganisms of greatest concern due to the lack of new antibiotics continues to be *S. aureus*. This organism is the major cause of uncomplicated skin infections and serious invasive infections worldwide due to its numerous toxins, immune evasion factors, and its resistance to almost all available antibiotics. Effects resulting from a combination of antibiotic resistance and high virulence have been observed on several occasions in strains of methicillin-resistant *S. aureus* (MRSA). In the US, mortality from MRSA remains the highest for any antibiotic-resistant pathogen (Cheung et al., 2021).

The most recently approved anti-staphylococcal antibiotics include dalbavancin, tedizolid, oritavancin, and delafloxacin (Terreni et al., 2021), none of which belongs to a new structural class, so it is likely that bacteria resistant to them will soon be found as has happened with other antibiotics. Plants offer not only new structural scaffolds, but also the potential for different antibacterial mechanisms that have not yet been fully exploited; for example, inhibition of quorum sensing, inhibition of efflux pumps and other mechanisms that may even make it possible to rescue the use of antibiotics implicated in resistance (Gorlenko et al., 2020; Khare et al., 2021). The various secondary metabolites of plants include large groups such as the alkaloids, flavonoids, terpenes, and coumarins; several representatives of these groups have been found with good activity (Gibbons, 2004). Some coumarins are very active against microorganisms; for example, the compound 7-amino-4-methylcoumarin, isolated from the extract of an endophytic fungi of *Ginkgo biloba* L., exhibited broad-spectrum antibacterial and antifungal activity *in vitro* against *S. aureus* (MIC: 16 μg/ml), *Escherichia coli* (10 μg/ml), *Salmonella typhimurium* (15 μg/ml), *Salmonella enteritidis* (8.5 μg/ml), *Aeromonas hydrophila* (4 μg/ml), *Yersinia* sp. (12.5 μg/ml), *Shigella sp*. (6.3 μg/ml), *Vibrio parahaemolyticus* (12.5 μg/ml), and *Candida albicans* (15 μg/ml) (Liu et al., 2008). On the other hand, aspodelin A, isolated from the plant species *Asphodelus microcarpus* Salzm. and Viv., also showed broad-spectrum antibacterial activity against *S. aureus* (MIC: 16 μg/ml), *Escherichia coli* (4 μg/ml), and *Pseudomonas aeruginosa* (8 μg/ml) (El-Seedi, 2007).

Plants of the family Calophyllaceae and their genera *Mammea*, *Mesua*, and *Calophyllum* are good sources of coumarins (Rouger et al., 2019). These genera produce mammea coumarins, molecules with a very restricted structure in the plant kingdom, with a particular and unique nomenclature. They are uniquely characterized as being hydroxylated at C-5 and C-7; substituted at C-4 with phenyl, propyl, pentyl, methyl-propyl, or 1-acetoxypropyl residues and at C-6 and C-8 with an acyl or prenyl residue (geranyl in the case of surangin A and B). Based on the substituent at C-4, there are so far five designated series: mammea A (4-fenil), B (4-propil), C (4-pentil), D [4-(1-metilpropil)], and E (1-acetoxipropil) (Crombie et al., 1987). These types of coumarins are biologically active with antiprotozoal activity against *Leishmania amazonensis* (Brenzan et al., 2007, 2012), *L. brasiliensis* (Honda et al., 2010) and *Trypanosoma cruzi* (Reyes-Chilpa et al., 2008); cytotoxic activity (Reyes-Chilpa et al., 2004; Ito et al., 2006; Du et al., 2010; Gómez-Verjan et al., 2019), and others have shown good activity against dengue and chikungunya viruses (Gómez-Calderón et al., 2017).

*Mammea americana* L. (Calophyllaceae), a tree native to western India and northern South America (Brenzan et al., 2008) and best known for its insecticidal use, has shown antibacterial activity against *S. aureus* (Yasunaka et al., 2005) and *Streptococcus mutans* (ATCC 25175) and *Porphyromonas gingivalis* (ATCC 33277), bacteria from the oral cavity (Herrera Herrera et al., 2014). In this study, the bioguided fractionation of the ethanol extract of *M. americana* seeds was carried out and the MICs of some of the anti-staphylococcal secondary metabolites present in the active fraction were determined.

## Materials and Methods

### Materials and Equipment

*Column chromatography (CC):* Silica gel 60 (230–400 mesh, Merck^®^), hexane (Hex), chloroform (CHCl_3_), ethyl acetate (AcOEt), acetone (Me_2_CO) and methanol (MeOH) brand analytical grade Merck. Thin layer chromatography (TLC): Silica gel 60 F_254_ 0.2 mm plates, Merck^®^ (Cat. 1.05729.0001); Silica gel 60 F_254_ 0.5 mm preparative plates, Merck brand (Cat. 1.05744.0001); Vanillin/H_2_SO_4_ plate developing reagent (1% vanillin in 10% H_2_SO_4_ solution in ethanol); UV lamp 254 and 365 nm. HPLC: HPLC Waters 1,515 Isocratic-UV/VIS detector 2,489; normal phase column PhenoSphere-NEXT (Phenomenex), 250 × 4.6 mm (i.d), 5 μm; reverse phase column C18 PhenoSphere-NEXT (Phenomenex), 250 × 4.6 mm (i.d), 5 μm; HPLC grade hexane, acetone and acetonitrile (Merck); water milliQ. NMR: ^1^H, ^13^C and 2D NMR spectra were recorded on a Bruker Fourier 300 spectrometer (Bruker Bio-Spin GmbH, Rheinstetten, Germany) operating at 300 MHz for ^1^H and 75 MHz for ^13^C NMR, using CDCl_3_ (Sigma, St Louis, Mo, USA) as the solvent, and TMS as an internal standard. Chemical shifts (δ) are reported in ppm, and the coupling constants (*J*) are reported in Hz. HPLC-QTOF-MS/MS: Chromatographic separation was carried out using a 1,260 Infinity II HPLC (Agilent Technologies), equipped with a variable wavelength detector (VWD) using an InfinityLab Proroshell 120 EC-C18 column, 100 × 4.6 mm (i.d), 2.7 μm (Agilent Technologies, USA) with a mobile phase flow of 0.3 ml/min, composed of water and 0.1% formic acid (A) and acetonitrile with 0.1% formic acid (B). The gradient profile was as follows: (0–1 min, 95%A; 1–13 min, 95–5%A; 13–17 min, 5–0%A; 17–26 min, 0–95%A). Mass analysis was obtained using a 6530 Q-ToF quadrupole time-of-flight tandem mass spectrometer (Agilent Technologies), with electrospray ionization (ESI), positive ion mode operator. The mass detector conditions were as follows: Capillary voltage +3.5 kV, nitrogen gas temperature 320°C, drying gas flow rate 8.0 L/min, nebulizer gas pressure 35 PSI, shredder voltage 135 V, skimmer 65 V, and RF OCT 750 V. The mass range in the MS and MS/MS experiments was m/z 100–1,200 and 50–1,200 at 3 spectra/s, respectively. MS and MS/MS data were obtained with the Agilent MassHunter purchase software (version 10.1) and processed in the Agilent MassHunter Qualitative Analysis 10.0 with the METLIN metabolite database (metlin.scripps.edu), providing an error mass less than 5 ppm. Melting point: Electrothermal melting point apparatus (Electrothermal IA9100X1). Antibacterial activity tests: cation adjusted Mueller Hinton broth (CAMHB); trypticase soy agar; trypticase soy broth (TSB); Mueller Hinton agar; dimethyl sulfoxide (DMSO); Vancomycin hydrochloride MP Biomedicals (LLC Cat No. 195540 Lot No QR14945); Gentamicin sulfate salt, Sigma-Aldrich (G1264-250 MG, Lot#048M4758V); Oxacillin sodium salt, Sigma-Aldrich (28221-1G, Batch# 89022). Optical density (OD) reading: Skanlt 4.1/Multiskan FC, Thermo Scientific spectrophotometer.

### Bioguided Chromatographic Fractionation and Isolation of Active Components From *M. americana* Extract

The fruits of *M. americana* were acquired from a farm (Mameyal) in the municipality of Turbaco, department of Bolívar in northern Colombia (10°19′05.2″N 75°24′31.3″W). The plant was identified at the herbarium of the Botanical Garden Guillermo Piñeres (Cartagena, Colombia) as *M. americana* L. and the voucher No. JBC 467 was assigned. The seeds were washed and subsequently the cover was removed. The endosperm of the seed (546,5 g) was dried, ground and subjected to cold maceration with 96% ethanol with successive extractions and filtration until the material was exhausted, which was verified by TLC. The filtrate was subjected to rotary evaporation at 40°C and the dry extract (62 g) encoded as FD-I-34S was stored at −20°C.

**FIGURE 1:**
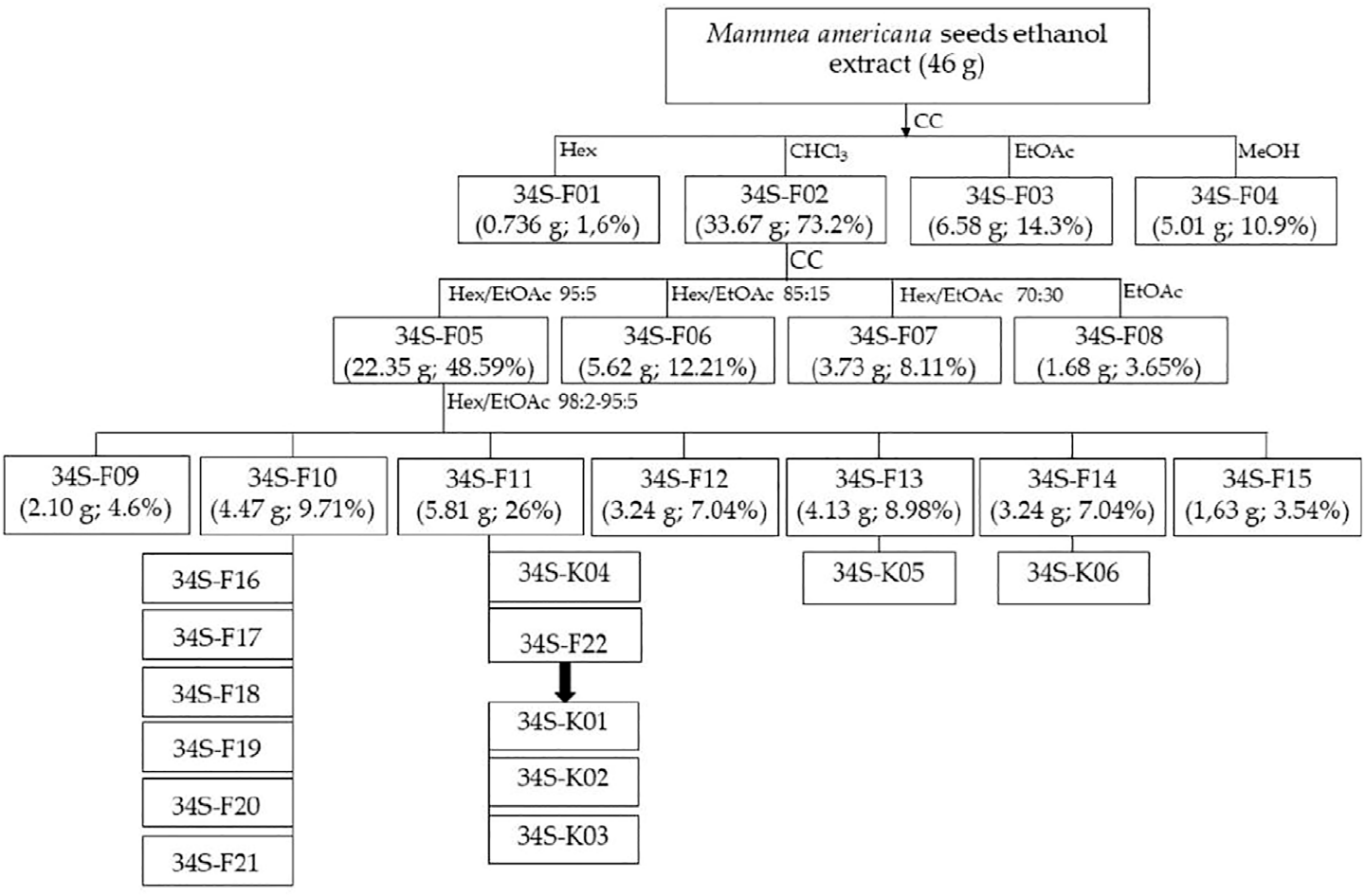
Scheme of the biodirected fractionation of the extract of *M. americana* seeds. CC, open column chromatography; Hex, hexane; CHCl3, chloroform; EtOAc, ethyl acetate; MeOH, methanol.

Forty-six grams of the ethanol extract were subjected to fractionation by normal phase open column chromatography using Silica gel 60 (230–400 mesh, Merck^®^) as stationary phase and elution gradient from hexane (100%), chloroform (100%), ethyl acetate (100%), and methanol (100%); four fractions (34S-F01 to 34S-F04) of different polarities were obtained (Figure 1). The fraction eluted with chloroform (34S-F02) was subjected to open column chromatography using gradient elution with different mixtures of hexane and ethyl acetate (95:5, 85:15, and 70:30), ending with 100% ethyl acetate, resulting in four fractions coded as 34S-F05, F06, F07, and F08. The active fraction 34S-F05 was subjected to column chromatography and eluted with a gradient elution from hexane/ethyl acetate (98:2) to hexane/ethyl acetate (95:5), giving rise to another seven fractions (from 34S-F09 to 34S-F15).

The active fraction 34S-F10 was subjected to open column chromatography with a gradient from hexane/ethyl acetate (99.5:0.5) to hexane/ethyl acetate (98:2) and subsequently to preparative thin layer chromatography in normal phase, using as mobile phase hexane/ethyl acetate 9:1. Six bands named 34S-F16, 34S-F17, 34S-F18, 34S-F19, 34S-F20, and 34S-F21, were isolated and tested against the *S. aureus* strains. All fractions were monitored by TLC and developed with UV light 254 and 366 nm and with vanillin/H_2_SO_4_ solution. Supplementary Figure S1 shows the TLC chromatographic profile of fractions 34S-F01 to 34S-F15.

The yellow pasty fraction 34S-F11 was washed several times with hexane; the mother liquors were combined, and the solvent was allowed to evaporate at laboratory temperature, producing transparent needles on the walls and bottom of the container (34S-F22). The crystals were subjected to reverse phase HPLC (acetonitrile/acetic acid 0.1% (7:3), flow 1.0 ml/min, 254 and 365 nm) and two coumarins (34S-K01 and 34S-K02) were isolated from them. A third coumarin (34S-K04) was isolated by preparative TLC from the mother liquor from the hexane wash of the 34S-F11 fraction, using hexane/ethyl acetate (9:1) as mobile phase. Three other coumarins were isolated by open column chromatography from fractions 34S-F13 and 34S-F14 as shown in Figure 1.

The structures of four isolated coumarins and two unseparated one, were determined by extensive NMR spectroscopic analysis of the 1D (^1^H and ^13^C) and 2D (^1^H–^1^H COSY, HSQC and HMBC) NMR spectra and by comparison with data reported in the literature (Prachyawarakorn et al., 2000; Yang et al., 2005; Yasunaka et al., 2005; Canning et al., 2013) and by HRESIMS for the structure of coumarins Mammea B/BA (34S-K01) and Mammea B/BC (34S-K02).

### Bacteria Strains and Culture

ATCC (American Type Culture Collection) methicillin-sensitive and methicillin-resistant *S. aureus* strains (MSSA and MRSA, respectively) were used. The bacterial strains were: ATCC 29213 (MSSA), ATCC 33591 (MRSA), ATCC 43300 (MRSA) and USA300-0114 (MRSA); ATCC 25922 (*Escherichia coli*), ATCC 700603 (*Klebsiella pneumoniae*) and ATCC 27853 (*Pseudomonas aeruginosa*). Clinical MSSA and MRSA strains isolated from pediatric patients from a hospital in the city of Cartagena-Colombia (Supplementary Table S1) were also used. Bacterial strains were maintained and prepared for the tests according to the protocol M07-A9 of the Clinical and Laboratory Standards Institute (CLSI, 2012). Briefly, from the stock (stored at −80°C) of each strain, primary cultures were prepared in plates of trypticase soy agar (TSA) or Mueller Hinton agar (MHA), which were kept for 4 weeks at 2–8°C (primary culture, P1). Secondary cultures (P2) were prepared from P1 in TSA or MHA and kept at 2–8°C for 1 week. A third culture (P3) was made from P2, in CAMHB, 12 h before each test to prepare the bacterial inoculum.

### Minimum Inhibitory Concentration of the Extract, Fractions and Compounds

The MIC (MIC_50_ and MIC_90_) was evaluated by the broth microdilution method following the protocol M07-A9 of the Clinical and Laboratory Standards Institute (CLSI, 2012), with some modifications. Briefly, solutions at 4,000 μg/ml of the extract, fractions and compounds prepared in dimethyl sulfoxide (DMSO), were diluted with CAMHB in the range 1–32.0 μg/ml (twice the concentration to be evaluated); when necessary, the compounds were diluted to 0.125 μg/ml. The final DMSO concentration was kept below 1%. A volume of 100 µl of each dilution was mixed with 100 µl of a bacterial suspension in CAMHB of 1 × 10^6^ CFU/ml to obtain a final suspension of 5 × 10^5^ CFU/ml. For each treatment, a non-inoculated sample served as blank to correct the optical density (OD) reading at 620 nm. Gentamicin, vancomycin, and oxacillin antibiotics served as positive inhibition controls, while 1% DMSO served as the vehicle control. The plates were incubated at 37°C for 18 h and read in a spectrophotometer at 620 nm. Tests were carried out three times in triplicate, at three different times. The formula of Quave et al. (Quave et al., 2008) with some modifications, was used for calculating the percent inhibition of each replicate; the mean of the concentration with 90 and 50% of growth inhibition was the MIC_90_ and MIC_50_, respectively:

$$\%\;inhibition=(1-\left(\frac{\left(ODt-ODbt\right)}{\left(ODv-ODbv\right)}\right)x100$$

ODt, OD_620_ for inoculated treatment; ODbt, OD_620_ for not inoculated treatment; ODv, OD_620_ for inoculated 1% DMSO; ODbv, OD_620_ for 1% DMSO not inoculated.

### Minimum Bactericidal Concentration of the Extract and MaBBA

Seven serial dilutions from the extract of seeds of *M. americana* (64, 32, 16, 8, 4, 2, and 1 μg/ml) and five serial dilutions from MaBBA (8, 4, 2, 1, and 0.5 μg/ml) were prepared in CAMHB. These solutions were inoculated with a suspension of *S. aureus* to reach 5 × 10^5^ CFU/ml in a final volume of 2 ml. The samples were incubated for 24 h at 37°C. 10 µl of the culture was seeded on Brain Heart Infusion (BHI) agar plates and incubated for 24–48 h; finally, the number of CFUs for each plate was recorded and the MBC determined (Hindler and Humphries, 2016). Tests were carried out with three technical replicates and three biological replicates (performed at three different times).

### Growth Curve of Methicillin Resistant *S. Aureus* Treated With Extract and Mammea B/BA

Seven serial dilutions of concentrations 8, 4, 2, 1, 0.5, 0.25, and 0.125 μg/ml of MaBBA and two dilutions of concentrations 64 and 1 μg/ml of extract were prepared in CAMHB and inoculated with a bacterial suspension of *S. aureus* ATCC 33591 to achieve 5x10^5^ UFC/ml in a final volume of 2 ml. A bacterial suspension with 1% DMSO was used as growth control and gentamicin at 2 μg/ml (MIC: 2 μg/ml) and vancomycin 1 μg/ml (MIC: 1 μg/ml) were used as control. 20 µl of 10^−2^, 10^−4^, 10^−6^, 10^−8^, and 10^−10^ dilutions, prepared from each sample at time 0 (before incubation), 4, 8, 12, and 24 h (after incubation), were spread on BHI agar plates and incubated at 37°C for 24–48 h (Hindler and Humphries, 2016). The number of CFUs for each plate was recorded and plotted versus time.

### Toxicity *in vivo* on *Caenorhabditis elegans*

The *in vivo* toxicity assay of the extract (FD-I-34S) and two fractions, 34S-F05 and 34S-F11, was carried out on *Caenorhabditis elegans* N2 Bristol. Briefly, batches of 10 ± 2 young adult worms (L4 instar) were transferred to 24-well microplates. The nematodes were treated with eight two-fold concentrations of FD-I-34S, 34S-F05, and 34S-F11 from 2 μg/L to 256 μg/L. The control group was set up with K medium containing 1% of DMSO. Mortality was measured after 24 h of exposure, for this, the number of living and dead organisms in each well was recorded. Worms that did not present movement within 30 s were considered dead. Three technical replicates were established for each treatment and the tests were performed in triplicate (three biological replicates) on different days and the mortality percentage was calculated (Tejeda-Benitez and Olivero-Verbel, 2016).

### Cytotoxicity on Human Fibroblasts

MRC-5 fibroblasts (ATCC^®^ CCL-171™, Manassas, VA, USA) were seeded (150.000 cells/ml) into a 96-well plate and incubated at 37°C, 5% CO_2_ and 99% relative humidity for 24 h. After that, cells were exposed to different concentrations (6.25–200 μg/ml) of the extract (FD-I-34S) for 48 h. Subsequently, cells were incubated with a MTT solution (0.25 mg/ml) for 4 h, and the generated formazan crystals were dissolved using DMSO and the OD_550_ was measured using a microplate reader (Multiskan Go, ThermoScientific, Waltham, MA, USA). The absorbance values were presented as percent growth of viable cells against untreated cells as control and expressed as the mean with confidence intervals (CI) of triplicate samples from three independent experiments.

### Biofilm Inhibition

Five serial dilutions of the ethanol extract of *M. americana* (1, 0.5, 0.25, 0.125, and 0.0625 μg/ml) and five of MaBBA (0.5, 0.25, 0.125, 0.0625 and 0.0312 μg/ml) were prepared in a 96-well plate using TSB supplemented with dextrose 2.5% and, subsequently inoculated with *S. aureus* USA-300 0114 as described in the MIC assay. The plate was incubated for 24 h at 37°C. After incubation, the OD at 620 nm was recorded in order to calculate growth inhibition. The biofilms in the microtiter plates were processed following a published protocol (O’Toole, 2011). The percent of biofilm inhibition was determined using DMSO as control (Quave et al., 2008). Tests were carried out with three technical replicates and three biological replicates.

### Statistical Analyses

All values are expressed as mean ± standard deviation (SD). Statistical analysis was carried out with GraphPad Prism 8.0.1. The means of the different treatments and the control were compared using one-way analysis of variance (ANOVA), followed by Tukey’s post hoc test. Statistical significance was considered at *p* < 0.05.

## Results

### Bioguided Chromatographic Fractionation and Isolation of Active Components From *M. americana* Extract

62 g of dry extract were obtained from 546.5 g of dry seeds of *M. americana* (yield 11.3%). From the chromatographic fractionation of 46 g of the ethanol extract of *M. americana* seeds, four fractions (34S-F01 to 34S-F04) of different polarities were obtained (Figure 1). The fraction eluted with chloroform (34S-F02) had the highest yield and the highest antibacterial activity against *S. aureus* strains (ATCC 29213, ATCC 33591, ATCC 43300 and USA300-0114), which, together with the fraction eluted with ethyl acetate, accounted for approximately 90% of the extract content, so it could be considered that components of the extract are mostly of medium polarity. This fraction produced four sub-fractions (Figure 1), of which 34S-F05 was the most active against *S. aureus*. From open column chromatographic fractionation with gradient elution of 34S-F05, two active subfractions named 34S-F10 and 34S-F11 were obtained.

**FIGURE 2:**
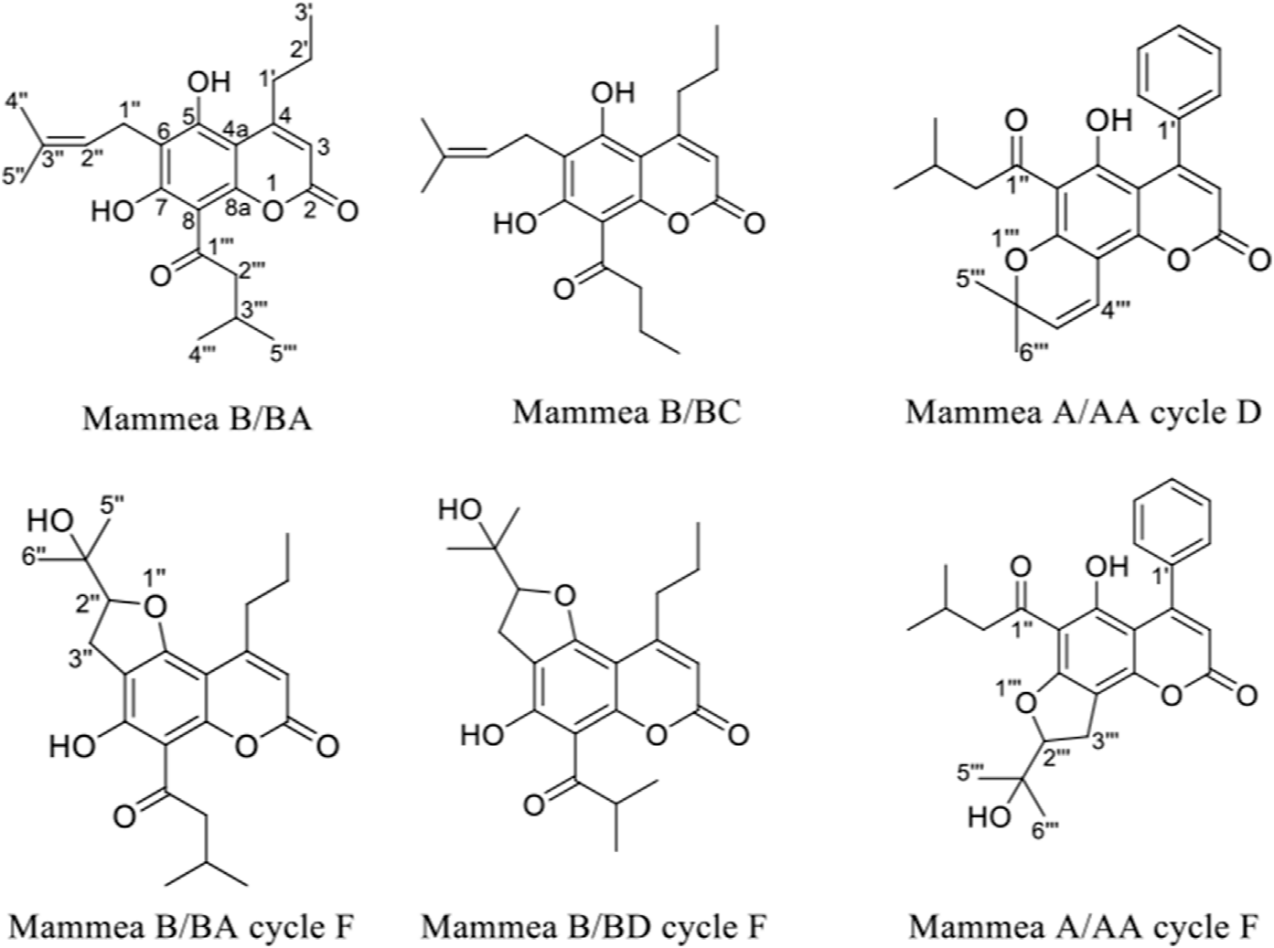
Structures of coumarins isolated from the extract of the seeds of *M. americana*.

The reverse phase HPLC chromatogram of the clean needles (34S-F22) showed the presence of three major peaks at 23.5, 25.3, and 35 min (Supplementary Figure S2). The ^1^H-NMR spectrum of the crystalline fraction (34S-F22) confirmed the mixture of three coumarin-like compounds from mamey, according to the reviewed literature (Supplementary Figure S3). After each peak had been individually collected, they were analyzed in Nuclear Magnetic Resonance (NMR) studies. The NMR spectrum of the compound at 25.3 min indicated the structure of a mammea coumarin called mammea B/BC (5 mg) while the peak at 35 min corresponded to mammea B/BA (6 mg). Mammea A/AA cycle D coumarin (10 mg) was isolated from fraction 34S-F11 by preparative TLC. From fraction 34S-F13, a mixture of mammea B/BA cycle F and mammea B/BD cycle F (40 mg) was obtained; finally, mammea A/AA cycle F (20 mg) was also isolated from fraction 34S-F14. The structures of the isolated coumarins are presented in Figure 2; ^1^H and ^13^C spectra are shown in Supplementary Figures S4–S17 and proton chemical shifts are described below:

Mammea B/BA (34S-K01): colorless crystals. Melting point: 121°C. vanillin: pink. Purity: 89%. 1H NMR (300 MHz, CDCl_3_): 14.64 (1H, s, 7-OH), 6.87 (1H, s, 5-OH), 6.03 (1H, s, H-3), 5.23 (1H, t, J = 9 Hz, H-2″), 3.93 (1H, sext, H-3‴), 3.50 (2H, d, J = 6 Hz, 1″), 2.93 (2H, t, J = 9 Hz, H-1′), 1.88 (3H, s, H-4″), 1.83 (3H, s, H-5″), 1.61 (1H, m, H-2′a), 1.49 (2H, m, H-2‴), 1.25 (3H, d, J = 9 Hz, H-5‴), 1.00 (7H, m, H-3'; H-4‴; H-2′b). HRESIMS *m/z* 373,20141 [M + H]^+^ corresponding to the molecular formula C_22_H_28_O_5_ (Supplementary Figure S18).

Mammea B/BC (34S-K02): colorless crystals. Melting point: 141°C. vanillin: pink. Purity: 93%. 1H NMR (300 MHz, CDCl_3_): 14.67 (1H, s, 7-OH), 6.87 (1H, s, 5-OH), 6.02 (1H, s, H-3), 5.22 (1H, t, J = 6 Hz, H-2″), 3.49 (1H, d, J = 6 Hz, H-1″), 3.29 (2H, d, J = 9 Hz, 3‴), 2.92 (2H, t, J = 6 Hz, H-1′), 1.88 (3H, s, H-4″), 1.83 (3H, s, H-5″), 1.77 (2H, m, 2‴), 1.64 (1H, m, H-2′), 1.02 (3H, d, H-4‴; H-3′). HRESIMS *m/z* 359,18543 [M + H]^+^ corresponding to the molecular formula C_21_H_26_O_5_ (Supplementary Figure S19).

Mammea A/AA ciclo D (34S-K04): yellow crystals. Melting point: 142°C. vanillin: green. Purity: 87%. 1H NMR (300 MHz, CDCl_3_): 14.80 (1H, s, 5-OH), 7.40 (3H, m, H-2'; H-4'; H-6′), 7.31 (2H, m, H-3'; H-5′), 6.89 (1H, d, J = 12.0 Hz, H-4‴), 5.99 (1H, s, H-3), 5.63 (1H, d, J = 9.0 Hz, H-3‴), 2.95 (2H, d, J = 9.0 Hz, H-2″), 2.22 (1H, sept, H-3″), 1.67 (6H, s, H-5‴; H-6‴), 0.96 (6H, d, J = 6.0 Hz, H-4''; H-5″).

Mammea A/AA ciclo F (34S-K06): white powder. Melting point: 107°C. vanillin: colorless. Purity: 85%. 1H NMR (300 MHz, CDCl_3_): 14.52 (1H, s, 5-OH), 7.40 (2H, m, H-2'; H-6′), 7.31 (2H, m, H-4′), 7.29 (1H, m, H-3'; H-5′), 5.95 (1H, s, H-3), 4.91 (1H, t, J = 9.0 Hz, H-2‴), 3.32 (2H, d, z J = 9.0 Hz, H-3‴), 2.98 (2H, m, 2″), 2.83 (1H, dd, J = 9.0, 6.0 Hz, H-2a''), 3.00 (1H, dd, J = 6.0, 6.0 Hz, H-2b''), 1.44 (3H, s, H-5‴), 1.32 (3H, s, H-6‴), 0.98 (3H, d, J = 6.0 Hz, H-4″), 0.96 (3H, d, J = 6.0 Hz, H-5″).

Mixture of Mammea B/BA ciclo F and Mammea B/BD ciclo F (34S-K05), yellow powder. Melting point: 119–122°C. vanillin: colorless. Some characteristic signals in the NMR spectra are: Mammea B/BA ciclo F: 1H NMR (300 MHz, CDCl_3_): 14.21 (1H, s, 7-OH), 6.00 (1H, s, H-3), 4.86 (1H, t, J = 9 Hz, H-2″), 3.90 (1H, m, H-2‴), 3.17 (2H, d, J = 6 Hz, 3″), 2.84 (2H, t, J = 6 Hz, H-1′), 1.61 (1H, m, H-2′), 1.45 (3H, s, H-5″), 1.28 (3H, s, H-6″), 1.24 (3H, m, H-3‴), 1.02 (3H, s, H-3′), 0.97 (3H, s, H-4‴). Mammea B/BD ciclo F: 1H NMR (300 MHz, CDCl_3_): 14.21 (1H, s, 7-OH), 6.00 (1H, s, H-3), 4.86 (1H, t, J = 9 Hz, H-2″), 3.90 (1H, m, H-2‴), 3.17 (2H, d, J = 9 Hz, 3″), 2.84 (2H, t, J = 9 Hz, H-1′), 1.61 (1H, m, H-2′), 1.45 (3H, s, H-5″), 1.28 (3H, s, H-6″), 1.24 (3H, m, H-3‴), 1.02 (3H, s, H-3′), 0.97 (3H, s, H-4‴).

### Antibacterial Activity of the Extract Fractions and Compounds of *M. americana* Seeds

The ethanol extract of the seeds of *M. americana* (FD-I-34S) inhibited the growth of all *S. aureus* strains (ATCC and clinical) at low concentrations, with MIC_90_ values between 2 and 4 μg/ml and MIC_50_ between 0.5 and 1 μg/ml (Tables 1, 2). The activity of the extract was concentrated in the 34S-F02 fraction with MIC_90_ values of 2–8 μg/ml. The lowest (34S-F01) and highest (34S-F03 and 34S-F04) polarity fractions were found to be inactive up to a concentration of 16 μg/ml. The 34S-F05 fraction obtained from 34S-F02 remained in the MIC range of 2–8 μg/ml, but in the fractions obtained from it, coded as 34S-F10 and 34S-F11, the MIC decreased, being between 1 and 4 μg/ml. Through TLC analysis (Silica gel 60 F_254_, hexane/ethyl acetate 8:2) it was possible to characterize the fractions 34S-F10 and 34S-F11, clearly distinguishable by their pink, violet and green spots when developed with 1% vanillin (Supplementary Figure S1). The band encoded as 34S-F21, isolated from 34S-F10, showed strong activity against methicillin-resistant *S. aureus*, but it was not possible to isolate a compound in sufficient quantity for analysis by NMR.

**TABLE 1:** Minimum inhibitory concentration (µg/ml) of the extract (FD-I-34S), fractions, and compounds isolated from the seeds of *M. americana* against ATCC strains of *S. aureus* sensitive (ATCC 29213) and resistant to methicillin (ATCC 33591, ATCC 43300, and USA300-0114).

Extract /Fraction	ATCC 29213	ATCC 33591	ATCC 43300	USA300-0114
**FD-I-34S**	**4**	**2**	**2**	**4**
34S-F01	>16	>16	>16	>16
34S-F02	8	8	2	4
34S-F03	>16	>16	>16	>16
34S-F04	>16	>16	>16	>16
34S-F02				
** 34S-F05**	8	4	2	4
34S-F06	16	8	8	8
34S-F07	>16	>16	>16	>16
34S-F08	>16	>16	>16	>16
**34S-F05**				
34S-F09	>16	>16	>16	>16
34S-F10	4	2	2	4
34S-F11	4	1	1	4
34S-F12	16	8	2	16
34S-F13	16	8	16	16
34S-F14	16	8	16	>16
34S-F15	>16	>16	>16	>16
**34S-F10**				
34S-F16	>16	>16	>16	>16
34S-F17	>16	>16	>16	>16
34S-F18	>16	>16	>16	>16
34S-F19	>16	>16	>16	>16
34S-F20	>16	16	>16	>16
34S-F21	2	1	1	2
**34S-F11**				
34S-F22 (Crystalline fraction, mixture of 34S-K01, 34S-K02, 34S-K03)	4	2	2	4
Mammea B/BA (34S-K01)	1	0.5	0.5	1
Mammea B/BC (34S-K02)	>16	16	16	>16
Mammea A/AA cycle D (34S-K04)	>16	>16	>16	>16
**34S-F13**				
Mammea B/BA cycle F + Mammea B/BD cycle F (34S-K05)	>16	>16	>16	>16
**34S-F14**				
Mammea A/AA cycle F (34S-K06)	>16	>16	>16	>16
**Antibiotics**				
Gentamicin	0.25	2.0	>8	-
Vancomycin	1	1	1	1
Oxacillin	0.125	>8	>8	>8

Due to the low MIC value shown by the extract and due to its low solubility in the culture broth, all fractions and compounds were evaluated up to a maximum concentration of 16 μg/ml against *S. aureus*.

**TABLE 2:** Minimum inhibitory concentration (µg/ml) and minimum bactericidal concentration (µg/ml) of the extract of *M. americana* and MaBBA in different strains of clinical isolates of *S. aureus*.

*S. aureus* strain	*M. americana* extract			MaBBA			Van.	Gent.	Oxac.
	MIC_90_	MIC_50_	MBC	MIC_90_	MIC_50_	MBC	MIC_90_	MIC_90_	MIC_90_
ATCC 29213^a^	4	1.0	>64	1	0.25	>8	1	0.25	0.125
ATCC 33591	2	0.5	>64	0.5	0.25	>8	1	2	>8
Sau-02	4	0.5	>64	0.5	0.25	>8	0.5	0.5	>8
Sau-09	4	1	>64	0.5	0.25	>8	0.5	0.5	>8
Sau-11^a^	2	0.5	>64	0.5	0.25	>8	0.5	0.25	0.25
Sau-12	2	0.5	>64	1.0	0.25	>8	0.5	0.25	>8
Sau-17	4	1	>64	0.5	0.25	>8	0.5	0.5	>8
Sau-19	2	0.5	>64	0.5	0.25	>8	0.5	0.25	8
Sau-25^a^	4	1	>64	0.5	0.25	>8	0.5	0.25	0.125
Sau-27^a^	4	1	>64	0.5	0.25	>8	1	0.25	0.25
Sau-39^a^	4	1	>64	0.5	0.25	>8	0.5	0.25	0.25
Sau-44^a^	4	0.5	>64	0.5	0.25	>8	0.5	0.25	0.25

a Meticillin-resistant *S. aureus*.

From the needle-shaped crystals from fraction 34S-F11, three coumarins were isolated and evaluated. MaBBA was the most active with a MIC_90_ between 0.5 and 1 μg/ml against ATCC reference and the clinical strains (Table 2), while mammea B/BC was active at 16 μg/ml only in two methicillin-resistant strains. The third coumarin isolated from the crystalline fraction was not active at a concentration of 16 μg/ml. The coumarins mammea A/AA cycle D and Mammea A/A cycle F and the mixture of the coumarins mammea B/BA cycle F and mammea B/BD cycle F were also not active up to a concentration of 16 μg/ml. The activity of the extract and the fractions against Gram-negative bacteria was nil, up to a concentration of 32 μg/ml (Supplementary Table S2).

**FIGURE 3:**
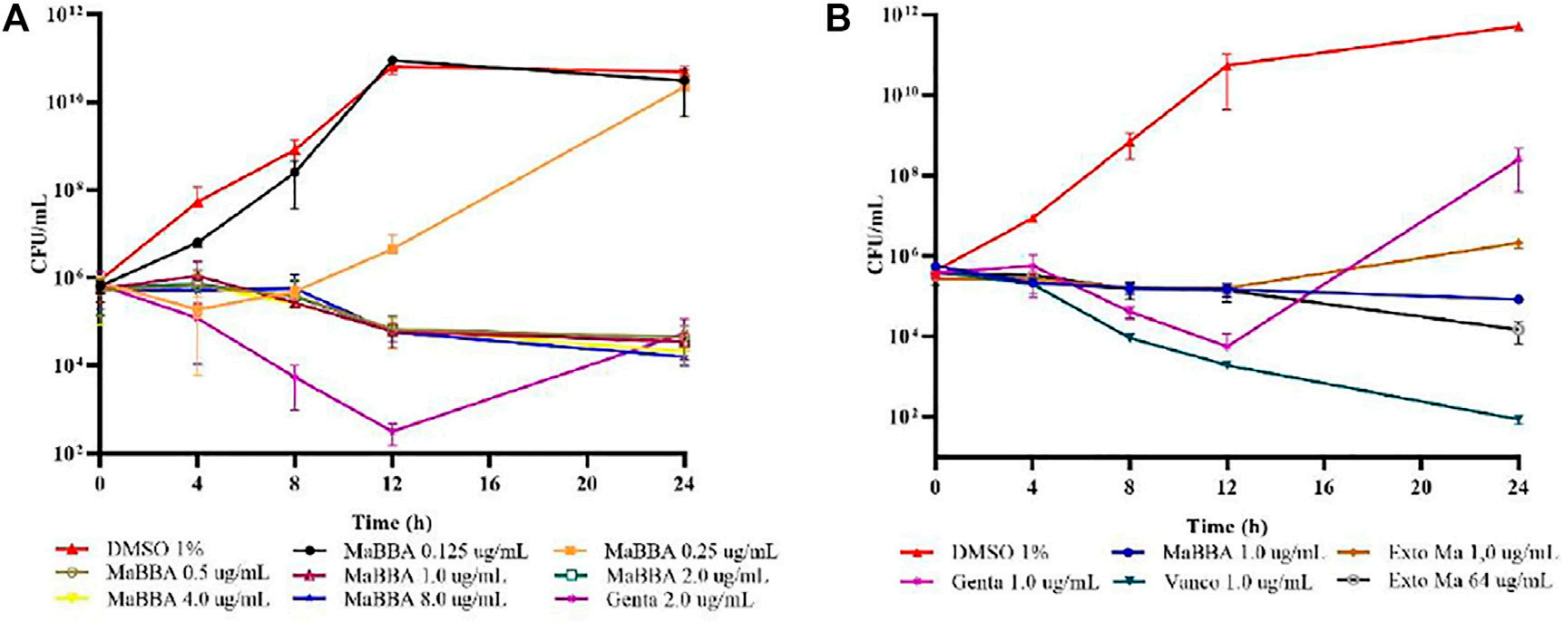
Growth curves of *S. aureus* ATCC 33591: **(A)** treatment with inhibitory and subinhibitory concentrations of mammea B/BA (MaBBA); **(B)** treatment with gentamicin (Genta), vancomycin (Vanco), MaBBA at a concentration of 1 μg/ml and ethanolic extract of *M. americana* at a concentration of 1 and 64 μg/ml. Each value represents the mean ± standard deviation of CFU/ml of bacteria.

The evaluation of the minimum bactericidal concentration (MBC) of the extract of *M. americana* (tested up to a concentration of 64 μg/ml) and of the active compound MaBBA (tested up to a concentration of 8 μg/ml), against *S. aureus* ATCC 33591, indicated that these did not cause a reduction in the CFU/ml count greater than 3log10, and consequently, they are considered to have a bacteriostatic effect; this effect is also observed in the growth curves shown in Figures 3A,B. Therefore, the MBC is reported in the present study as a value above 64 μg/ml (extract) and 8 μg/ml (MaBBA), as shown in Table 2.

### Biofilm Inhibition

When evaluating the inhibitory activity of film formation by *S. aureus*, of the extract of *M. americana*, it was found that at a concentration of 0.5 μg/ml (concentration at which it showed less than 50% inhibition of growth), it only produced an inhibitory effect of 21.92%. Regarding the inhibitory activity of coumarin MaBBA, it was found that it inhibited biofilm formation by 43.01%, a concentration of 0.25 μg/ml, but with a growth reduction of 34.44%. Below concentrations of 0.5 μg/ml for the extract and 0.25 μg/ml for MaBBA, both were found to inhibit biofilm between 0 and 4% (Supplementary Table S3). Because at the concentrations where there was biofilm inhibition there was also a reduction in bacterial growth, we consider that neither the extract nor MaBBA had an inhibitory effect on biofilm formation.

### Toxicity *in vivo* on *C. elegans* and *in vitro* on MRC-5 Fibroblasts

At the concentrations evaluated (2, 4, 8, 16, 32, 64, 128, and 256 μg/ml), the extract (FD-I-34S) and the fractions active as antistaphylococcals (34S-F05 and 34S-F11) did not produce mortality in the nematode *C. elegans*. On the other hand, FD-I-34S affects the growth of normal fibroblasts at higher concentrations (IC_50_: 48.98) than those that inhibit *S. aureus* strains growth (Supplementary Figure S20). The selectivity indexes (IS) calculated for some *S. aureus* strains included in this study are shown in Table 3.

**TABLE 3:** Cytotoxicity of FD-I-34S on MRC-5 cells and selective indexes against *S. aureus* strains.

	Selectivity index			
IC_50_/LC_50_	ATCC^®^ 29213	ATCC^®^ 33591	ATCC^®^ 43300	USA300-0,114
48.98 (68.55–35.81)^a^	12.25	24.49	24.49	12.25
>256^b^	64	128	64	128

a Inhibitory Concentration 50 (IC_50_) MRC-5 (95% CI): Results are presented as the mean and confidence intervals (CI) of triplicate samples from three independent experiments. Selectivity index (SI) = IC_50_ Fibroblast/MIC_90_ *S. aureus* strain.

b Lethal Concentration 50 (LC_50_) *C. elegans*. Selectivity index (SI) = LC_50_ *C. elegans*/MIC_90_ *S. aureus* strain.

## Discussion

The use of *M. americana* as an anti-infective is not widely cited in the literature, however, it is mentioned that its seeds without the embryo are used to prepare an anti-helmintic infusion and it is believed that the infusion of fresh or dried leaves helps in cases of intermittent fever; leaf extracts are effective against *Mycobacterium tuberculosis* and, in addition, antimalarial properties have been suggested (Herrera Herrera et al., 2014).

The antibacterial activity of mamey seed extract has been previously demonstrated in some studies against Gram-positive bacteria; in two of these investigations, some MIC values are reported. Herrera et al. reported that the oily and ethanolic fractions obtained from the extract of mamey seeds, inhibited the growth of *Streptococcus mutans* (ATCC 25175) and *Porphyromonas gingivalis* (ATCC 33277); the oily phase was the most active against *S. mutans* (MIC: 15.6 μg/ml), showing bactericidal effect at concentrations greater than 125 μg/ml (Herrera Herrera et al., 2014). Yasunaka et al., in 2005 reported that the MIC of *M. americana* seed extract against *S. aureus* was 2 μg/ml for a methicillin-sensitive strain (209P) and 4–8 μg/ml for methicillin resistant strain (Yasunaka et al., 2005). The results presented in this article are in agreement with the results of Yasunaka et al., since the extract of *M. americana* seeds in our study reached a MIC_90_ of 2–4 μg/ml in both MRSA and MSSA. Although, the MIC_90_ value is the one considered clinically relevant, the MIC_50_ value of 0.5–1.0 μg/ml of mamey extract is interesting due to a 50% inhibition achieved at low concentrations is important in the pharmacodynamics of some antibiotics to define dosage regimens, which should be corroborated at the clinical level. *M. americana* is one of the most active plant against *S. aureus*, according to a recently published review by Quave et al., 2008), of 739 species of plants with antibacterial activity, it was found that the species *Sambucus nigra* L. (Adoxaceae), *Echinops kebericho* Mesfin (Asteraceae), *Mikania glomerata* Spreng. (Asteraceae), *Curcuma longa* L. (Zingiberaceae), and *Combretum album* Pers (Combretaceae) show the best average MIC values (3.5–16 μg/ml) (Chassagne et al., 2021), which are above the MIC of *M. americana* reported in this article (MIC_90_ of 2–4 μg/ml).

This article describes for the first time the bioguided fractionation of the extract of *M. americana* seeds against different sensitive and resistant strains of *S. aureus*. From the most active fraction of this extract, four mamey coumarin-type compounds were isolated, namely, mammea B/BA, mammea B/BC, mammea A/AA cycle D, mammea A/AA cycle F and a mixture of mammea B/BA cycle F + mammea B/BD cycle F. Evaluation of the antibacterial activity against *S. aureus* of these coumarins showed that mammea B/BA was the most active. Mammea B/BA was first isolated from *M. americana* by Morris M.P. and Pagán in 1952 from a sample called mamein, which also contained two other coumarins, mammea B/BB and mammea B/BC (Morris and Pagán, 1953; Djerassi et al., 1958, 1959, 1960; Crombie and Games, 1966). Mammea B/BA was also isolated from the bark of *Mammea africana*, by Ouahouo et al. (2004), who demonstrated its anti-staphylococcal effect using the disk diffusion method, presenting an inhibition halo of 18 mm (Ouahouo et al., 2004); it should be noted that at the time of writing this article, the MIC value for this compound was not reported in the literature reviewed, so it is considered that this is the first time that the MIC_90_ of 0.5–1 μg/ml has been reported and a MIC_50_ value of 0.25 μg/ml, in reference and clinical MSSA and MRSA.

Our results indicate that mammea B/BA has the same anti-staphylococcal potency as the antibiotic vancomycin (MIC: 0.5–1.0 μg/ml) in all strains evaluated in this study. Regarding the MIC_50_ mammea B/BA, it behaved better than vancomycin, since coumarin reached an inhibition of 57% ± 8 at 0.25 μg/ml among all the strains evaluated, while vancomycin only reached 23% ± 7 in the lowest concentration following the MIC_90_ value, therefore, a MIC_50_ value could not be defined for this antibiotic.

The MBC:MIC ratio defines whether an antibiotic is bactericidal or bacteriostatic; when the ratio is ≤ 4, the antibiotic is considered to have a bactericidal effect. In the case of the *M. americana* extract and the MaBBA compound, both showed a bacteriostatic effect, since the MBC:MIC ratio is greater than 16. The growth curves of the methicillin-resistant *S. aureus* strain ATCC 33591 in the presence of the extract and MaBBA, confirmed a bacteriostatic effect for both (Figures 3A,B). As can be seen, up to a concentration of 8 μg/ml of MaBBA, this compound reduced the CFU/ml count only by 1 Log10 at 24 h of treatment, and no significant differences (*p* < 0.05) were found in the number of CFU/ml at each time, for the five concentrations evaluated above the MIC_90_. Figure 3B shows the differences in the growth curves of MaBBA, vancomycin and gentamicin at the same concentration (1 μg/ml) which showed the potential of active coumarin isolated from the seeds of *M. americana*, as a new natural antibiotic against methicillin resistant *S. aureus*. It is observed that the two antibiotics used as positive controls, and the coumarin, inhibited growth up to 12 h of treatment; MaBBA behaved as bacteriostatic, while gentamicin and vancomycin showed a bactericidal tendency; however, at the end of 24 h of treatment, only vancomycin behaved as bactericidal. Gentamicin did not inhibit the growth of *S. aureus* after 12 h, reaching an increase in CFU/ml by two logarithmic units above MaBBA and six logarithmic units above vancomycin. This study demonstrates the *in vitro* efficacy of MaBBA, being superior to gentamicin and similar to vancomycin in inhibiting bacterial growth. Vancomycin is one of the first antibiotics to consider in deep or life-threatening MRSA infections, such as staphylococcal endocarditis (Liu et al., 2011). However, in some patients there is not a sufficient response due to the relatively low bactericidal activity of this antibiotic and also due to the surge of resistance (Maraolo et al., 2021). One of the strategies used is the combination of vancomycin with an aminoglycoside; however, the clinical utility of this combination has not yet been demonstrated and the risk of nephrotoxicity and ototoxicity is imminent (Mulazimoglu et al., 1996). MaBBA could replace gentamicin in combination with vancomycin, for which the synergy should be tested in future research.

MaBBA (MIC 0.5–1 μg/ml) turned out to be the most active of all coumarins with anti-staphylococcal activity isolated from the genera *Mammea*, *Calophyllum* and *Mesua*, published in the literature reviewed at the time of preparation of this article. Of the 16 coumarins isolated by Verotta et al., from *Mesua ferrea* L. flowers and evaluated against strains of *S. aureus* sensitive and resistant to methicillin, only four of them, mammea A/BB, mesuol, mammea A/AB, mammea A/AA, were active with MIC values between 2 and 64 μg/ml (12). Yasunaka et al. (2005), reported the anti-staphylococcal activity of mammea A/AA (MIC of 8 μg/ml) isolated from the fruit peels of *M. americana* and mammea A/BA (MIC of 1–2 μg/ml), isolated from *Calophyllum brasiliense* Cambess. leaves (Yasunaka et al., 2005).

The present work also shows the results of the antibacterial activity of the seed extract of *M. americana* and the compound MaBBA, against *E. coli*, *K. pneumoniae* and *P. aeurginosa* up to a concentration of 32 μg/ml, without present activity in none of the cases. According to Verotta et al. (2004), coumarins isolated from *Mesua ferrea*, evaluated against Gram-negative bacteria *E. coli*, *E. cloacae*, *S. marcescens*, *P. aeruginosa*, and *Acinetobacter* spp., were inactive up to a concentration 128 μg/ml; however, against the Gram-positive bacteria *Enterococcus faecalis* and *Enterococcus faecium*, the compounds mammea A/BB, mesuol, mammea A/AB, mammea A/AA showed activity in the MIC range of 8–16 μg/ml. Canning et al. (2013), reported that mammea A/AA coumarin isolated from *Mammea africana* G. Don, was active against two Gram-positive bacteria, *Clostridium difficile* (MIC: 0.25 μg/ml) and *Streptococcus pneumoniae* (MIC: 0.25 μg/ml), and against a Gram-negative bacterium, *Campylobacter jejuni* with a MIC of 0.25 μg/ml (Canning et al., 2013). Apparently the mammea coumarins have a spectrum restricted only to Gram-positive bacteria, with the exception found for *Campylobacter jejuni*, allowing to suggest that they could be showing some specificity for the latter Gram-negative bacteria.

Onefact that is important to highlight from the review of the most active compounds of plant origin against *S. aureus* is that, like MaBBA, several of them are prenylated. As an example of these prenylated compounds we can mention a derivative of salicylic acid called 3-farnesyl-2-hydroxybenzoic acid (Rüegg et al., 2006), isolated and characterized from the plant species *Piper multiplinervium* C. DC. (Piperaceae; MIC: 6.25–12.5 μg/ml). On the other hand, the prenylated derivatives of benzophenone garcinol, isogarcinol and xanthozimol isolated from *Garcinia indica* (Thouars) Choisy and *Garcinia subelliptica* Merr., Clusiaceae, presented good anti-staphylococcal activity with a MIC range between 3.13 and 25 μg/ml (Iinuma et al., 1996). Similarly, the prenylated flavonoid soforaflavanona G (MIC 3.13–6.25 μg/ml) (Dastidar et al., 2001) and the acylphoroglucinol kielmeyerazine (MIC: 0.25–2 μg/ml) were also active against different *S. aureus* strains (Coqueiro et al., 2016). Lastly, rubraxanthone, isolated from the species *Garcinia dioica* Blume, Clusiaceae, presented MIC values between 0.313 and 1.25 μg/ml for MRSA and MSSA strains (Fukai et al., 2002), being one of the few plant metabolites that exceeds the anti-staphylococcal effect of mammea B/BA.

Regarding the structure of mammea coumarins and their antibacterial effect, we can say that the acyl group at C-8 and the prenyl group at C-6, seem to be essential for anti-staphylococcal activity, as shown by the activities reported for MaBBA (MIC: 0.5–1 μg/ml), isolated from mamey seeds in the present work and from mammea A/BA (MIC: 1–2 μg/ml) isolated from *Calophyllum brasiliense* Cambess., Clusiaceae (Yasunaka et al., 2005). According to the results obtained in our study, the structure of the acyl group at C-8 could be important for the activity against *S. aureus*, because mammea B/BC (MIC of 16 μg/ml), with a butyryl group at the C-8 carbon, it was less active than MaBBA which possesses a 3-methyl-butyryl. This is in some way in agreement with what was reported by Ouahouo et al. (2004), because the presence of the 2-methyl-butyryl group in C-8 of mammea B/BB could also be affecting the anti-staphylococcal activity, based on the inhibition halo (15 mm) for this molecule, unlike of the value of 18 mm reported for mammea B/BA in the same study. Switching the position of the prenyl and acyl, that is, acyl at C-6 and prenyl at C-8, also appears to affect antibacterial activity, as can be seen with the MIC value reported by (Yazunaka et al., 2005) for mammea A/AA (MIC: 8 μg/ml) and by Verotta et al. (2004) for the coumarins of the genus *Mesua*, which presented a MIC range of 2–64 μg/ml (Verotta et al., 2004; Yasunaka et al., 2005). The presence of the propyl and phenyl groups in the C-4 position of the mammea coumarins does not seem to affect the activity. Finally, the cyclization that involves the prenyl group at carbons C-6 and C-8 also causes a loss of activity, as could be observed in the case of cyclized coumarins (mammea A/AA cycle D, mammea A/AA cycle F, mammea B/BA cycle F and mammea B/BD cycle F), evaluated in our study, which presented a MIC value above 16 μg/ml.

In order to evaluate the safety of the mamey seed extract, the toxicity was evaluated *in vivo* on the nematode *C. elegans* (eukaryotic organism) and *in vitro* on human fibroblasts. Since the extract was innocuous when evaluated against *C. elegans* up to a concentration of 256 μg/ml and that the highest MIC_90_ value against *S. aureus* was 4 μg/ml, the IS in relation to the nematode should have a value higher than 64; likewise, the IS in relation to MRC-5 cells would correspond to a value greater than 12. The IS value is very important to select the promising species that can be used as a source of bioactive metabolites to develop new pharmaceutical products and we consider that the *M. americana* extract meets this criterion. The 34S-F11 fraction, where the compound MaBBA is present, was also innocuous against *C. elegans* at a concentration of 256 μg/ml; these results indicate that both, the mamey seed extract and the coumarin MaBBA could be considered relatively safe.

## Conclusion

The biodirected fractionation of the ethanolic extract of the seeds of *M. americana* against strains of *S. aureus* sensitive and resistant to methicillin, led to the isolation of four coumarins identified as mammea B/BA, mammea B/BC, mammea A/AA cyclo D and mammea A/AA cyclo F and a mixture of mammea B/BA cyclo F plus mammea B/BD cyclo F. Mammea B/BA was shown to be a very active compound with a MIC between 0.5 and 1.0 μg/ml, comparable to vancomycin (1.0 μg/ml), an antibiotic used clinically for the treatment of methicillin-resistant *S. aureus*, which allows proposing mammea BB/A as a natural alternative of plant origin for staphylococcal infections.

## Data Availability

The original contributions presented in the study are included in the article/Supplementary Material, further inquiries can be directed to the corresponding authors.
