# Supplementary figures and images for: Mammea B/BA Isolated From the Seeds of Mammea americana L. (Calophyllaceae) is a Potent Inhibitor of Methicillin-Resistant Staphylococcus aureus
Source: Front Pharmacol. 2022 Mar 11;13:826404. doi: 10.3389/fphar.2022.826404 (PMC8961693; doi:10.3389/fphar.2022.826404)

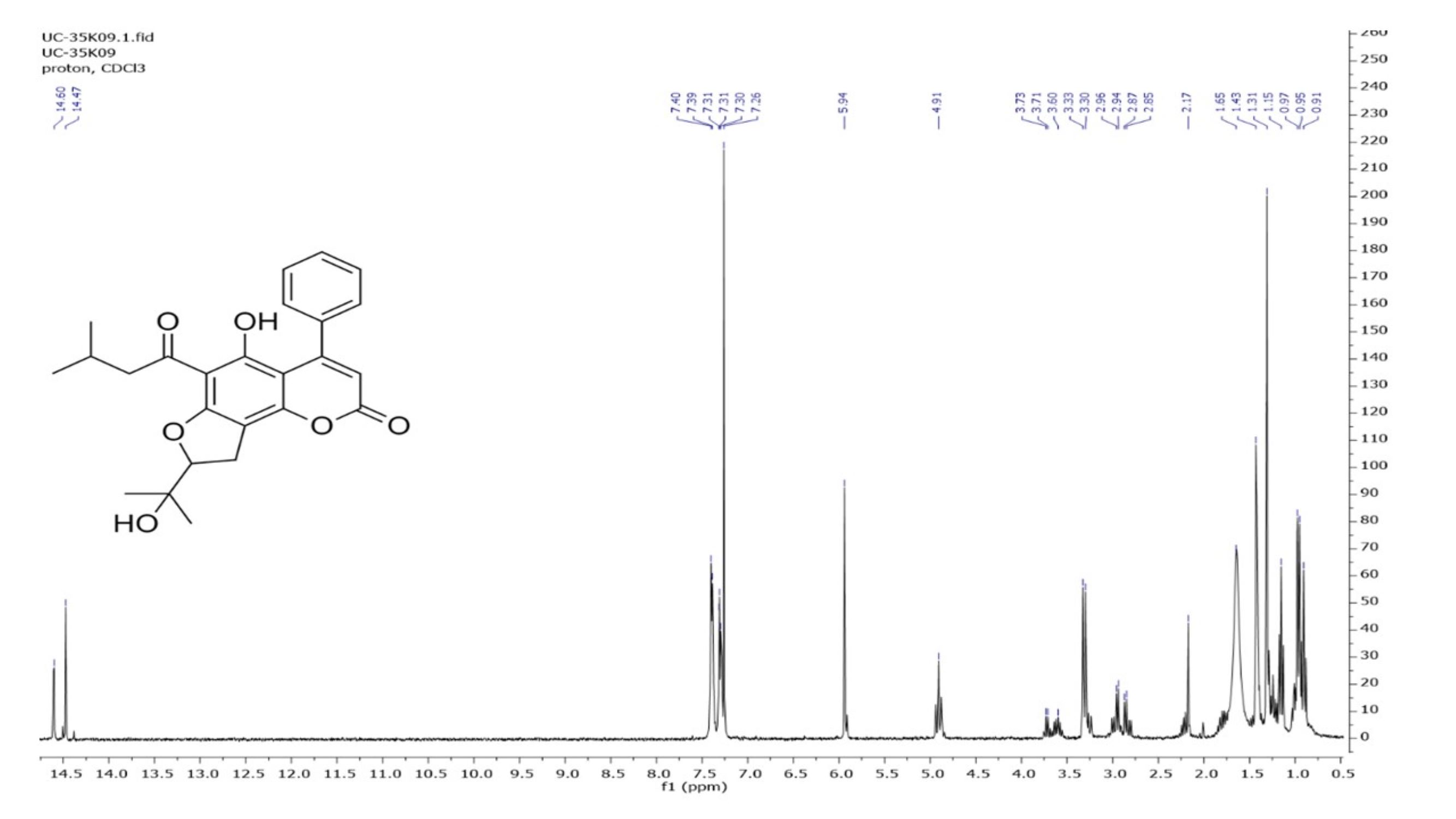

Supplement: Supplementary file 1 [file Image3.JPEG]

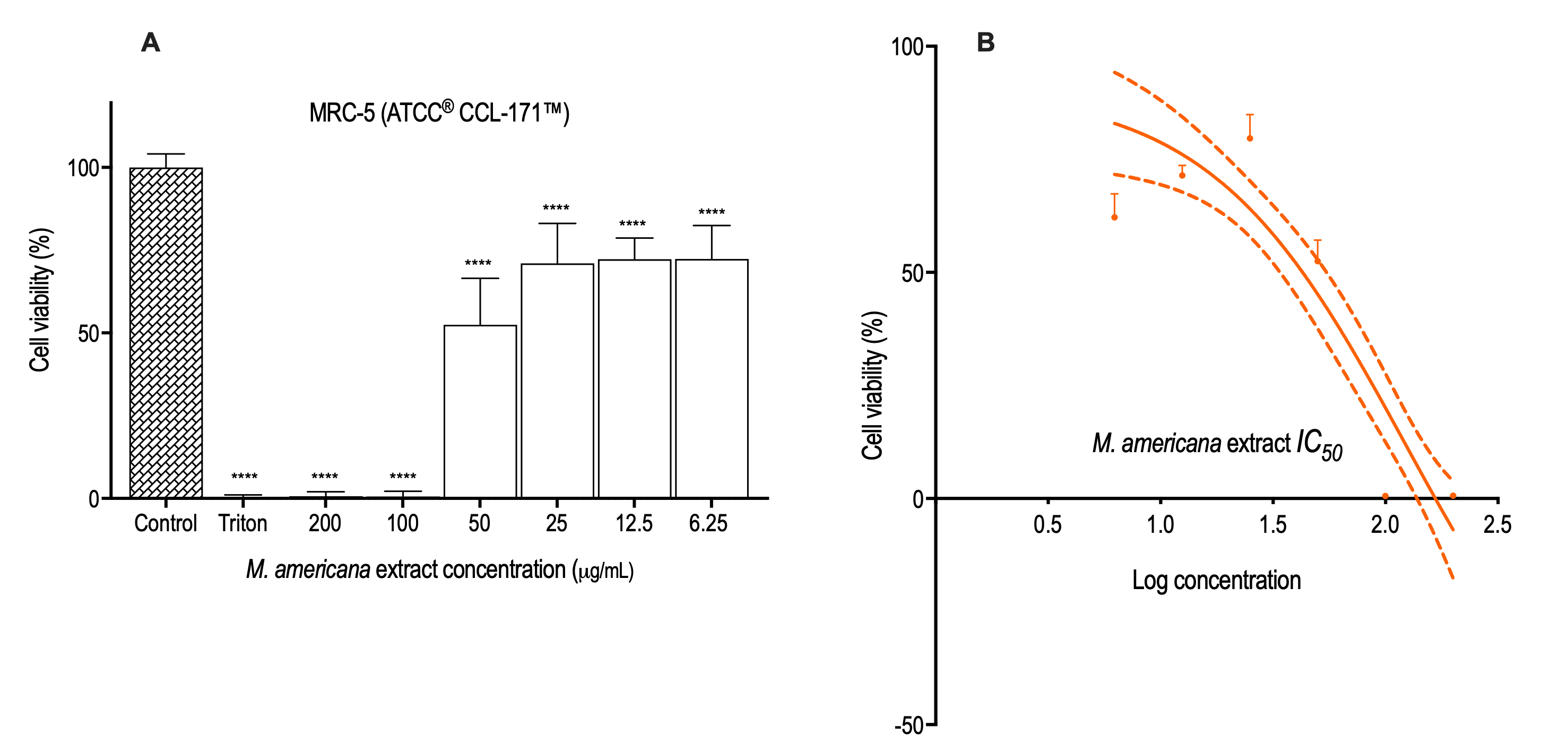

Supplement: Supplementary file 2 [file Image9.TIFF]

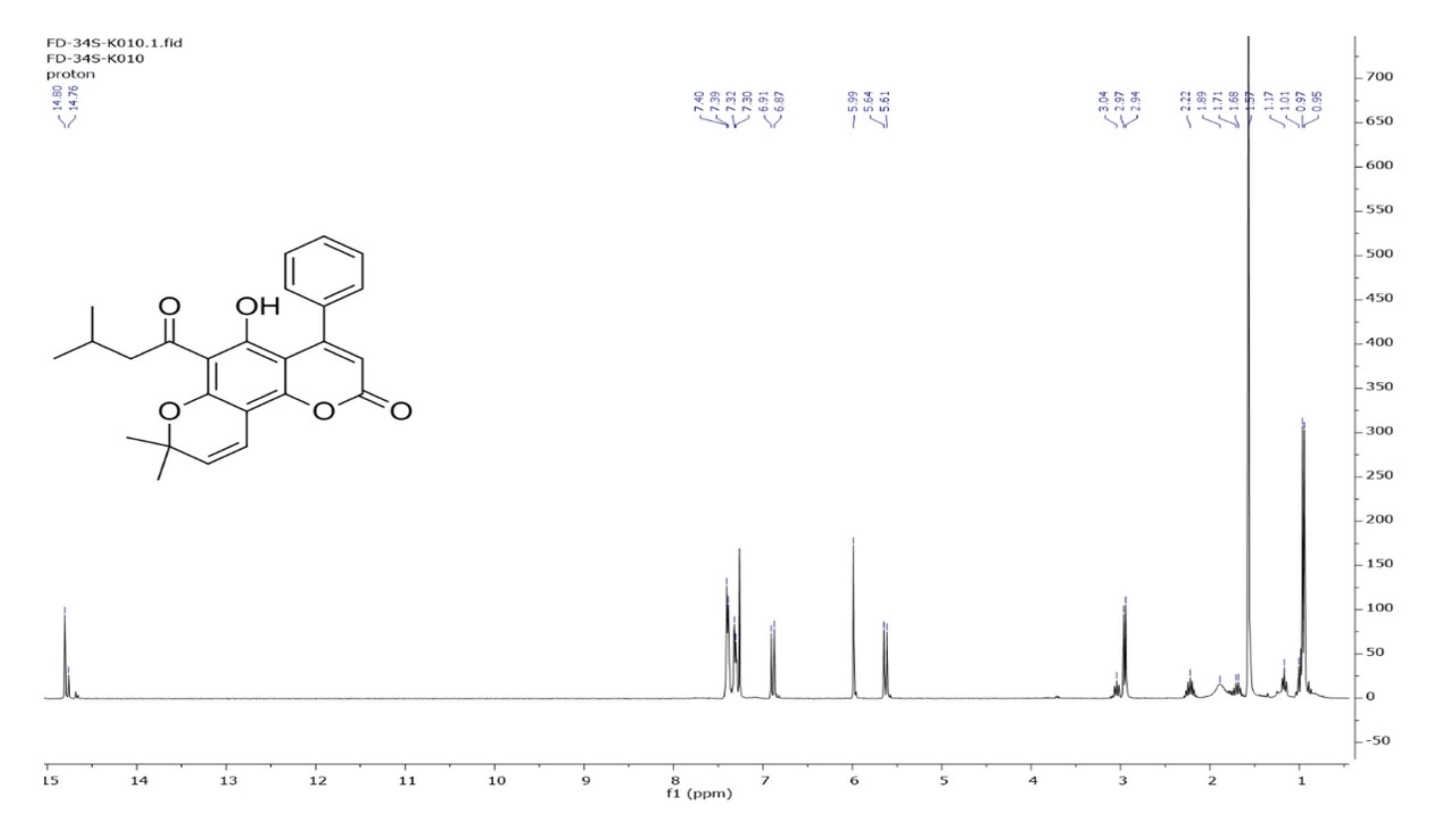

Supplement: Supplementary file 4 [file Image1.JPEG]

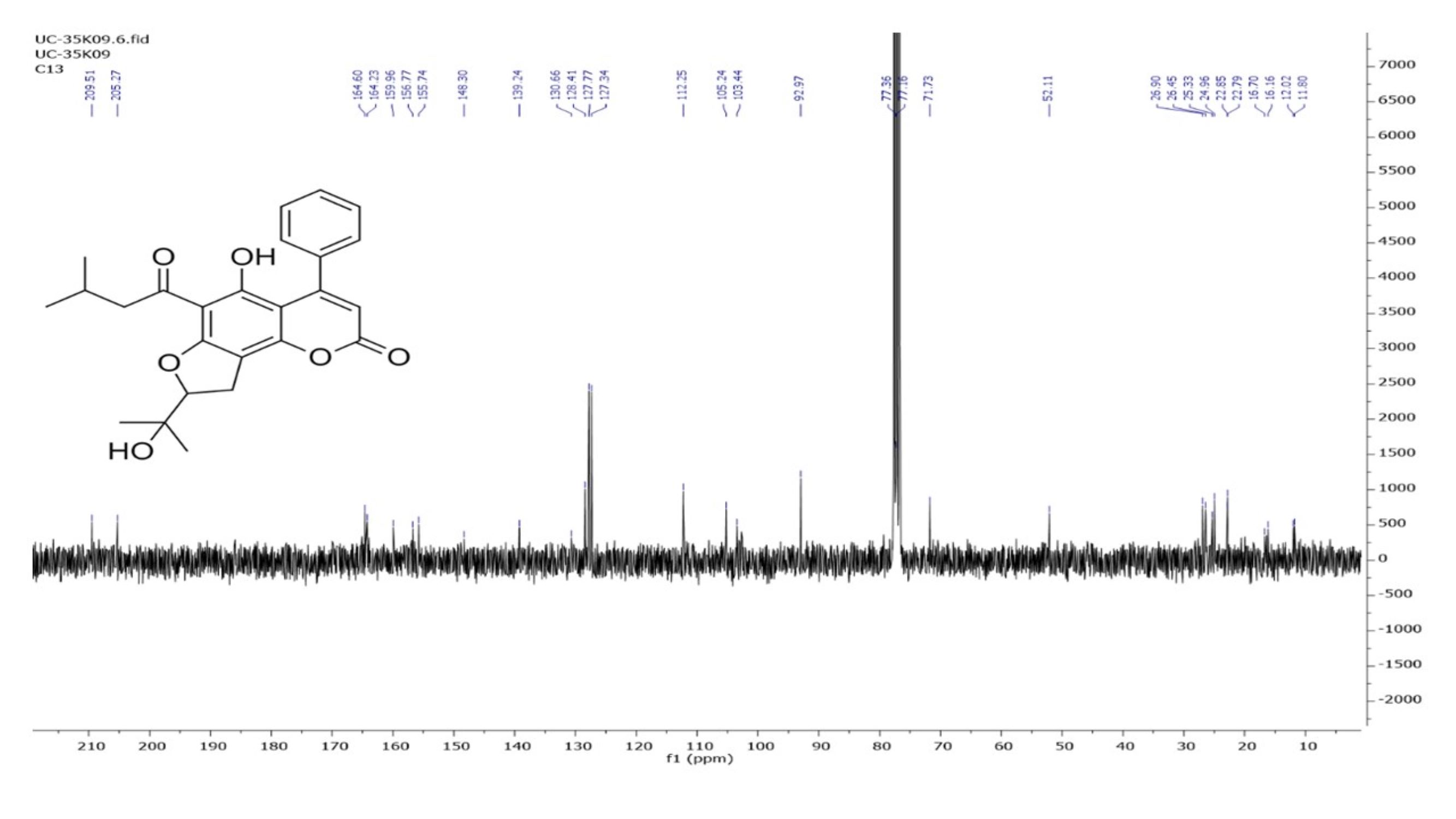

Supplement: Supplementary file 5 [file Image4.JPEG]

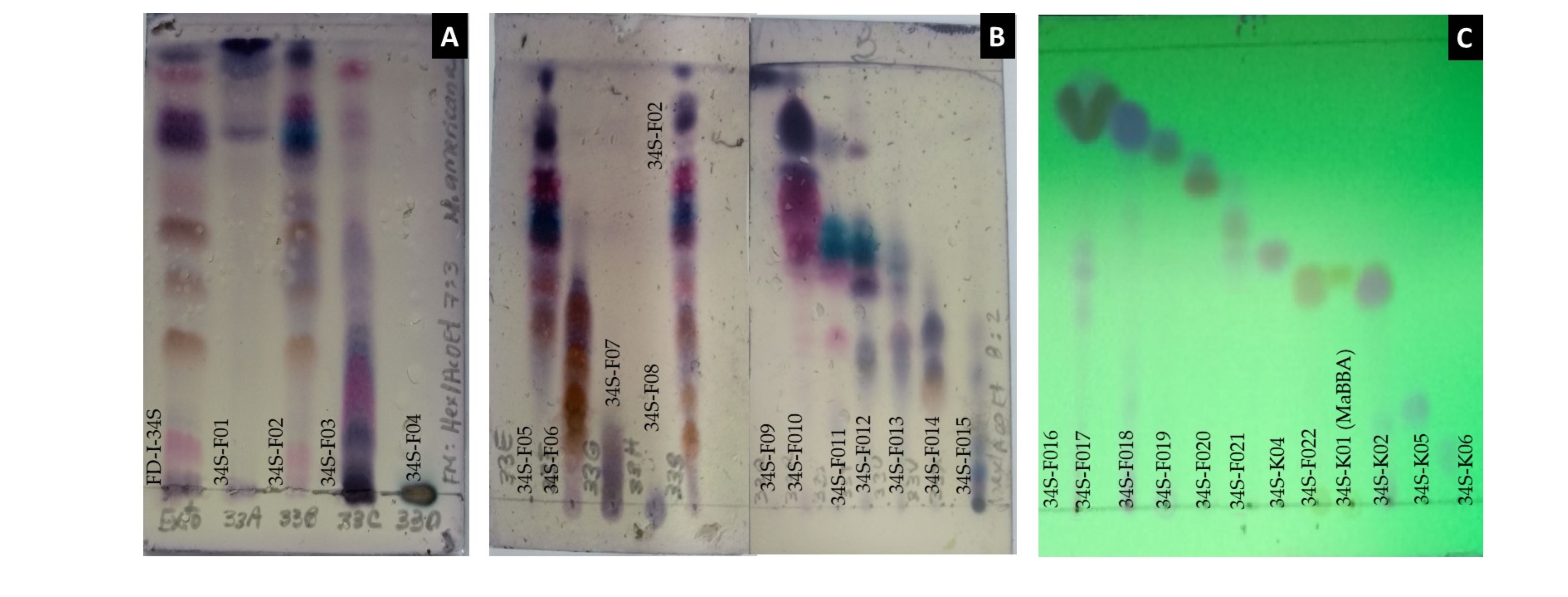

Supplement: Supplementary file 6 [file DataSheet1.zip › FIGURAS FRONTIERS/Figure S1.JPEG]

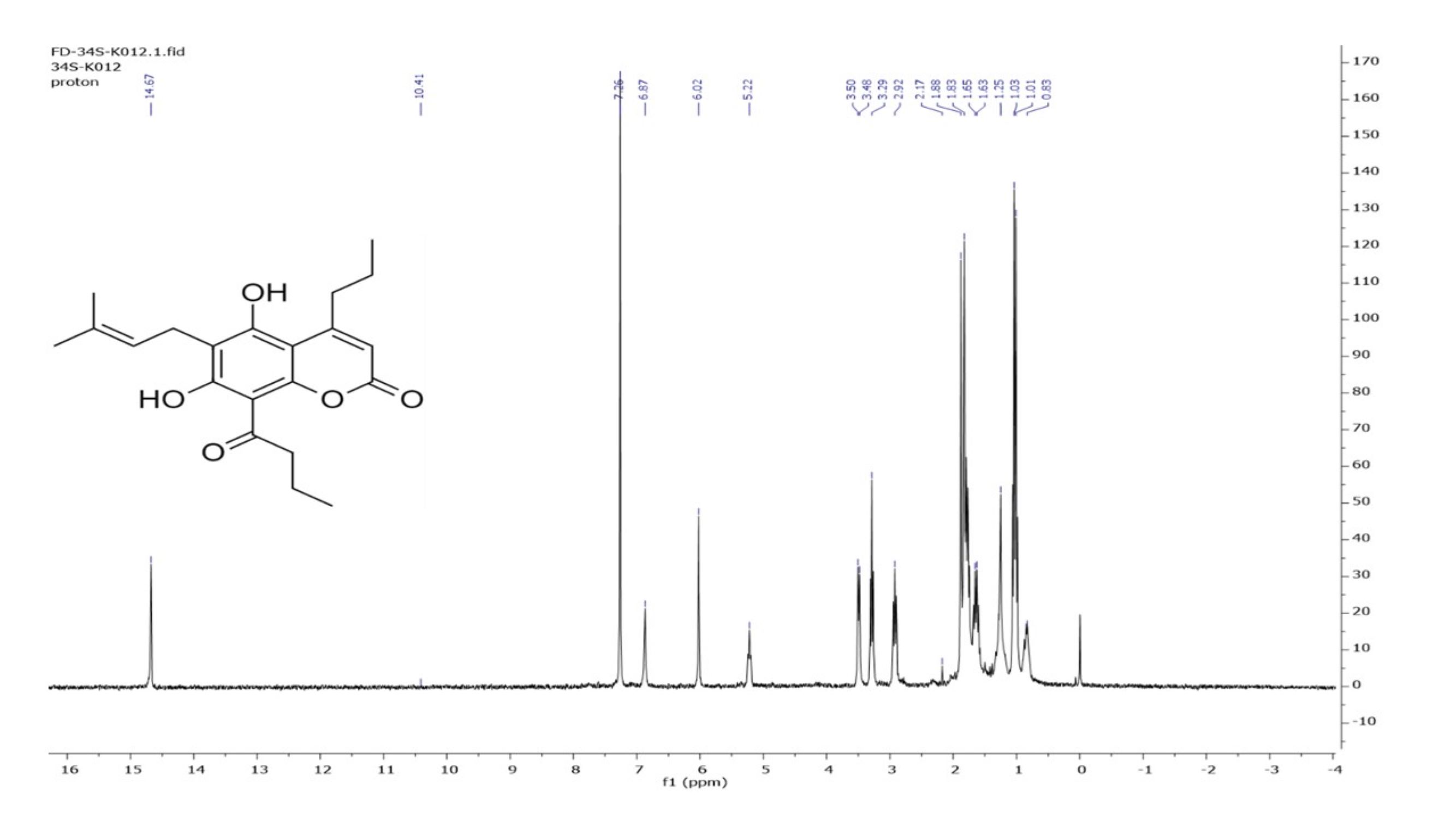

Supplement: Supplementary file 6 [file DataSheet1.zip › FIGURAS FRONTIERS/Figure S10.JPEG]

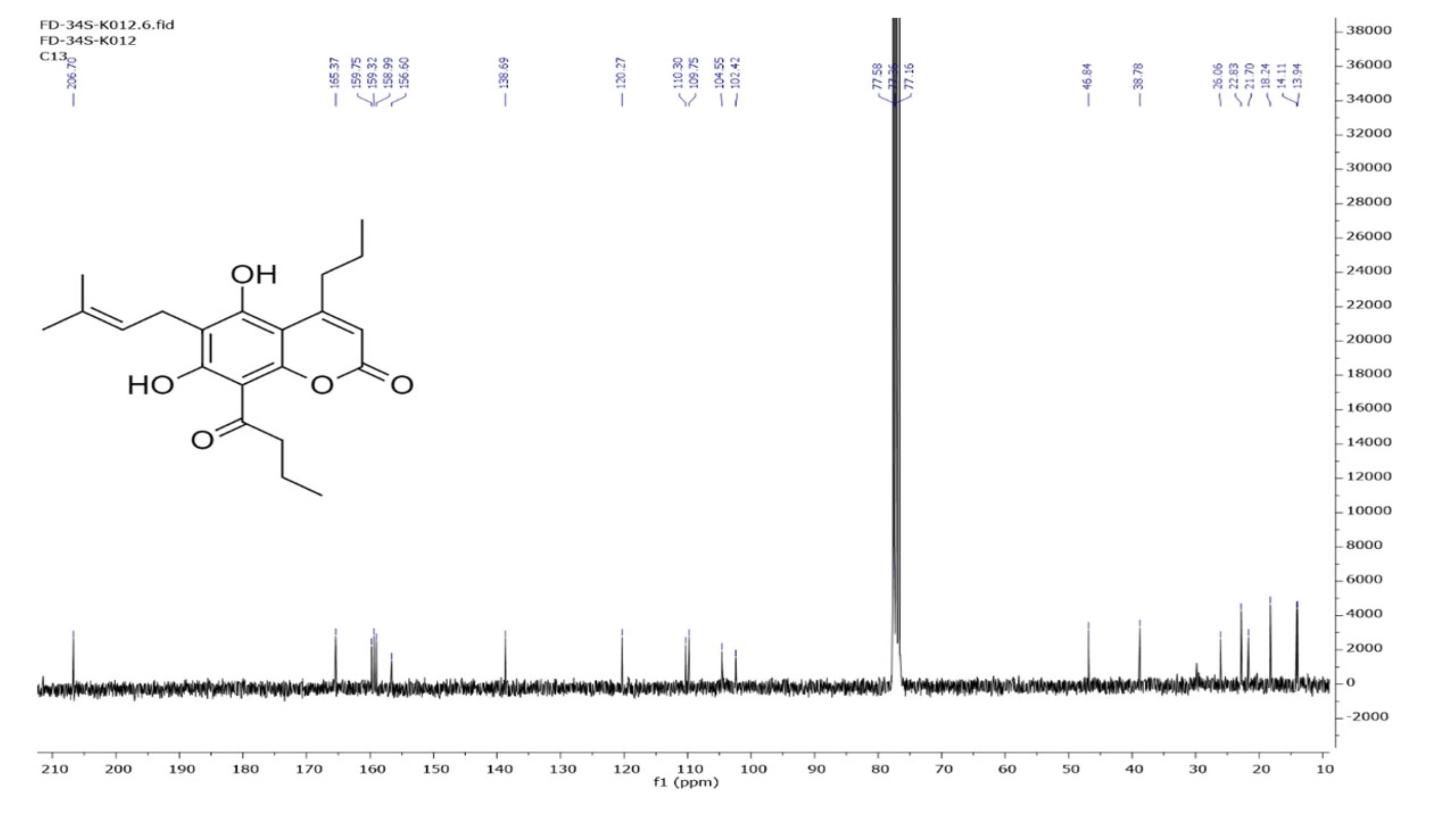

Supplement: Supplementary file 6 [file DataSheet1.zip › FIGURAS FRONTIERS/Figure S11.JPEG]

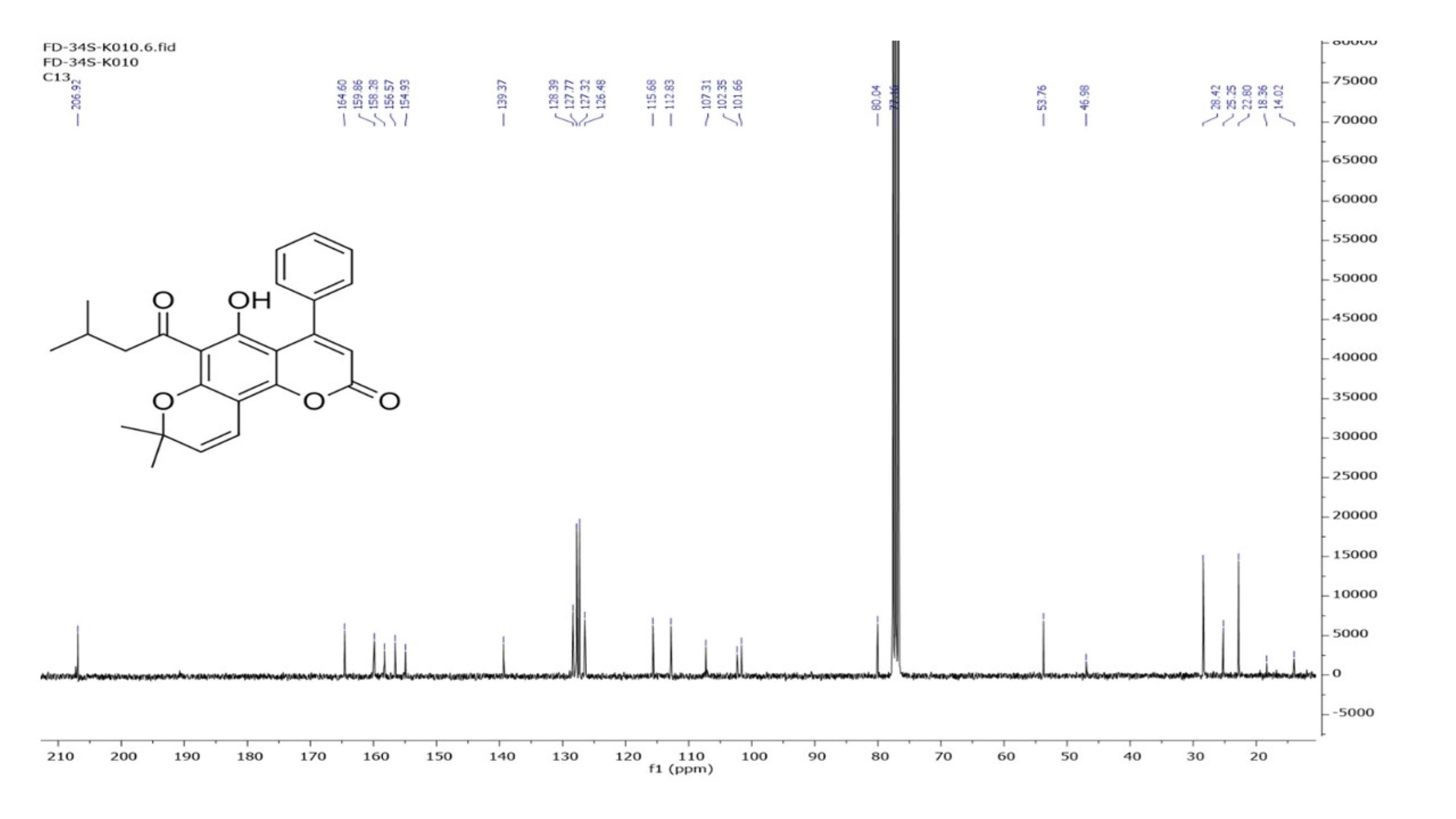

Supplement: Supplementary file 6 [file DataSheet1.zip › FIGURAS FRONTIERS/Figure S13.JPEG]

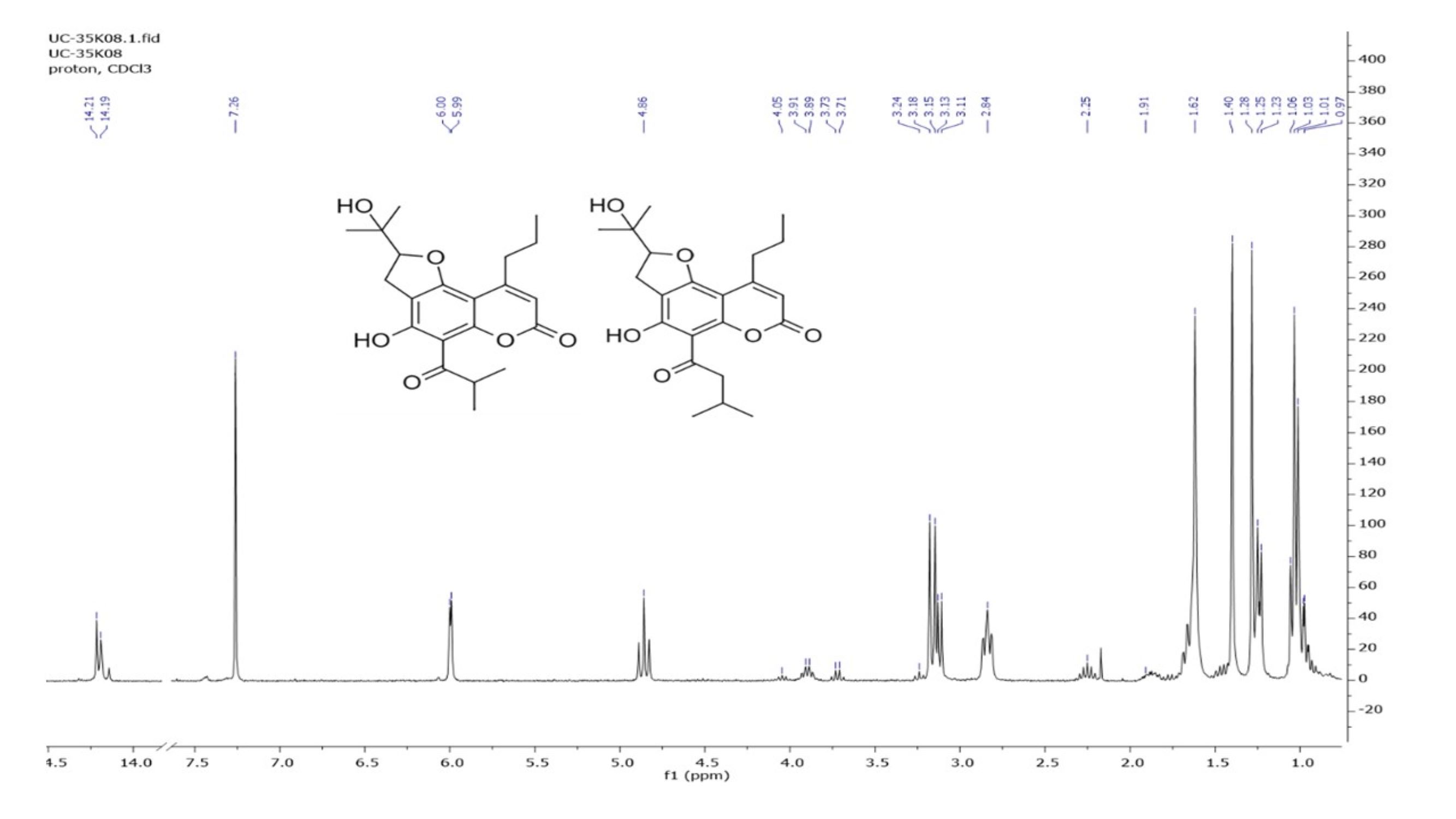

Supplement: Supplementary file 6 [file DataSheet1.zip › FIGURAS FRONTIERS/Figure S16.JPEG]

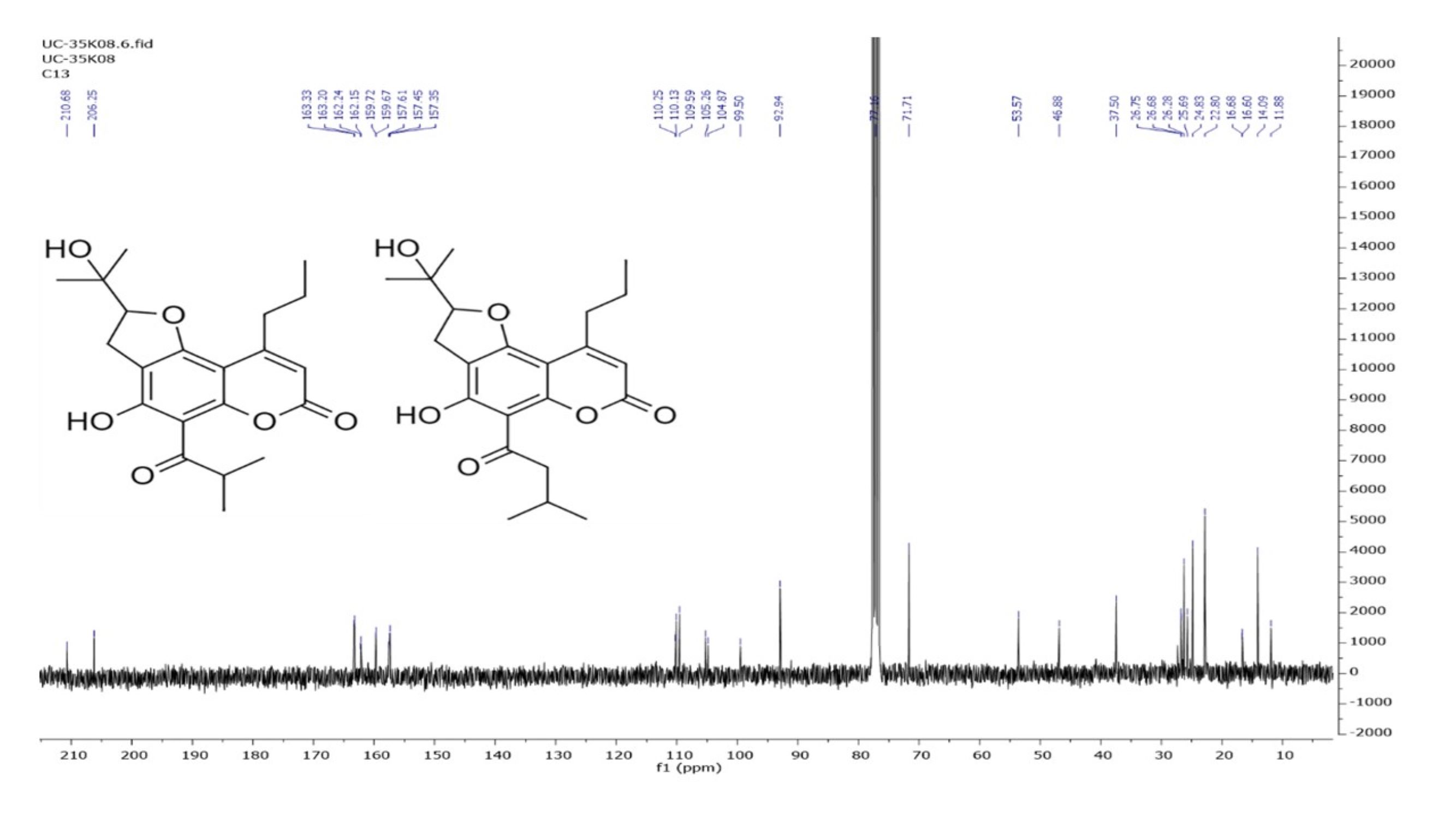

Supplement: Supplementary file 6 [file DataSheet1.zip › FIGURAS FRONTIERS/Figure S17.JPEG]

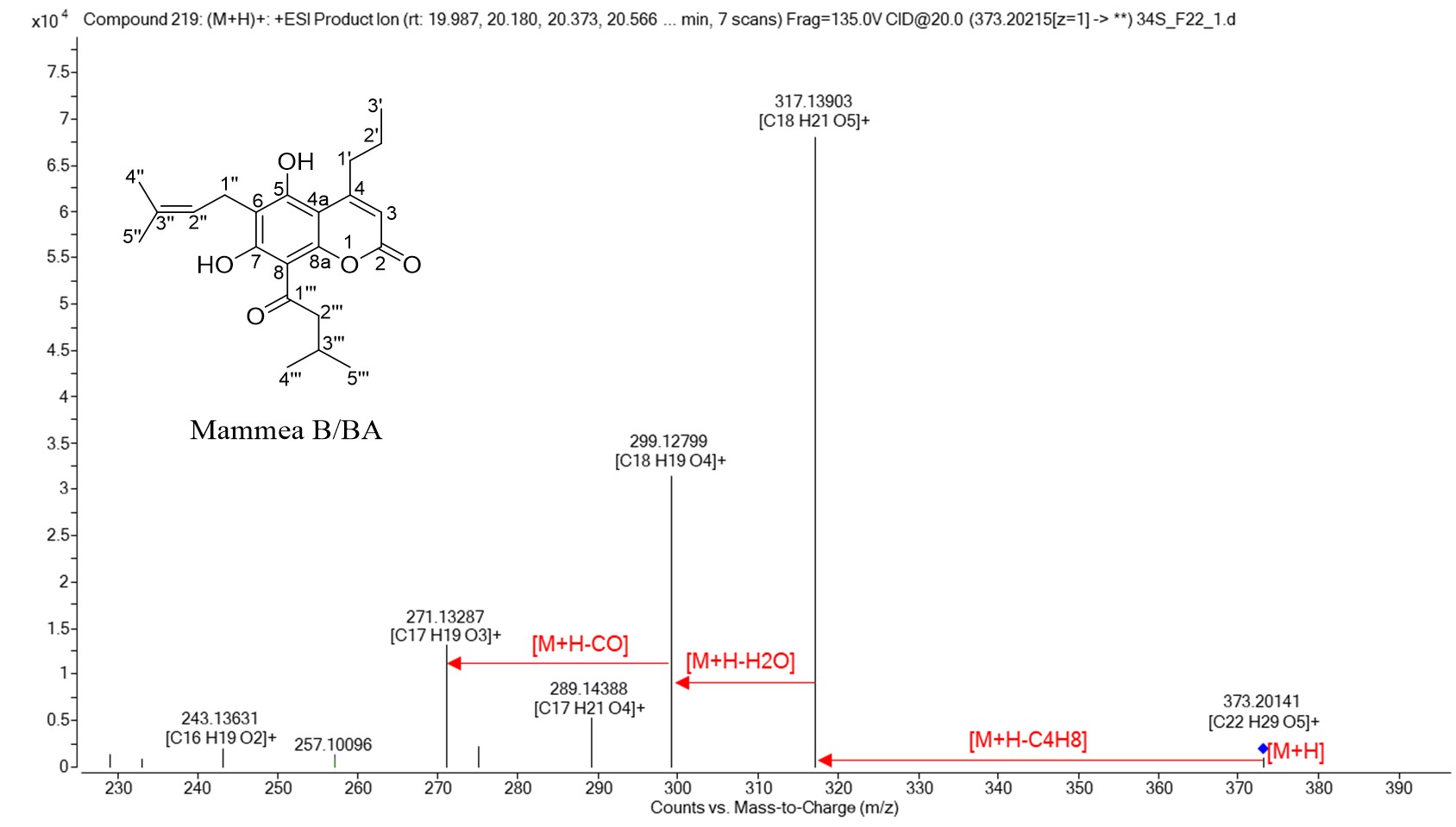

Supplement: Supplementary file 6 [file DataSheet1.zip › FIGURAS FRONTIERS/Figure S18.JPEG]

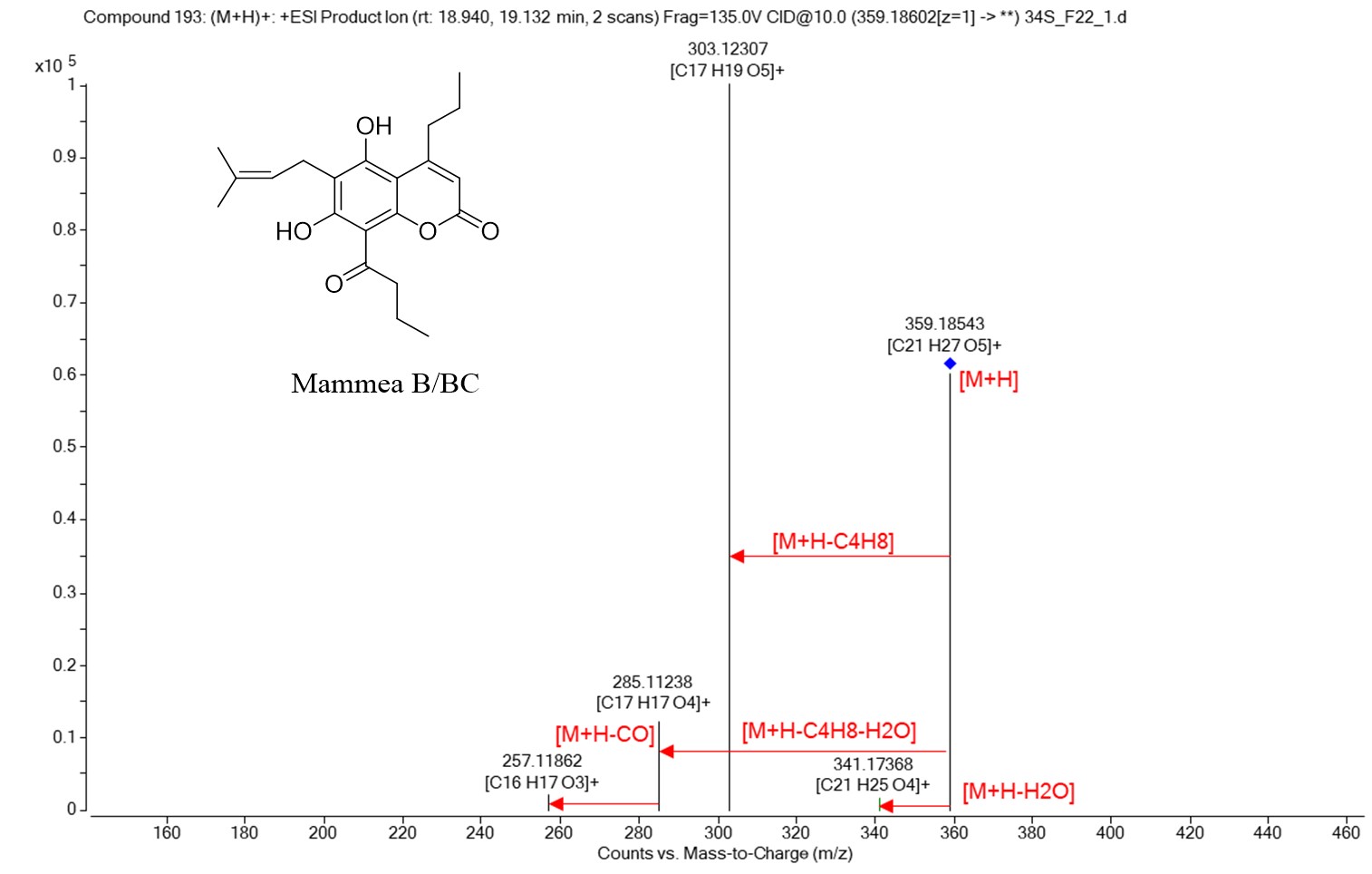

Supplement: Supplementary file 6 [file DataSheet1.zip › FIGURAS FRONTIERS/Figure s19.JPEG]

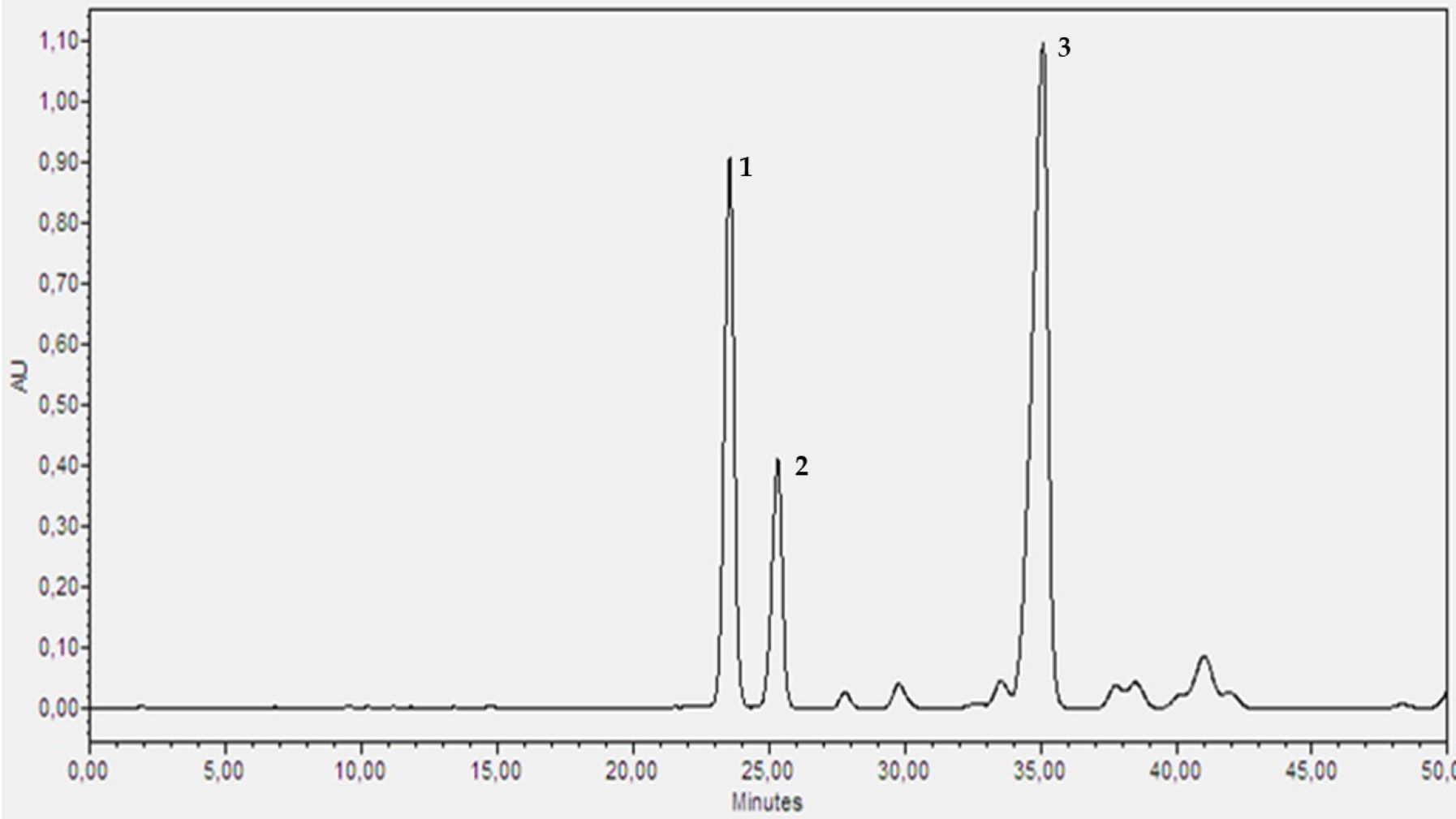

Supplement: Supplementary file 6 [file DataSheet1.zip › FIGURAS FRONTIERS/Figure S2.JPEG]

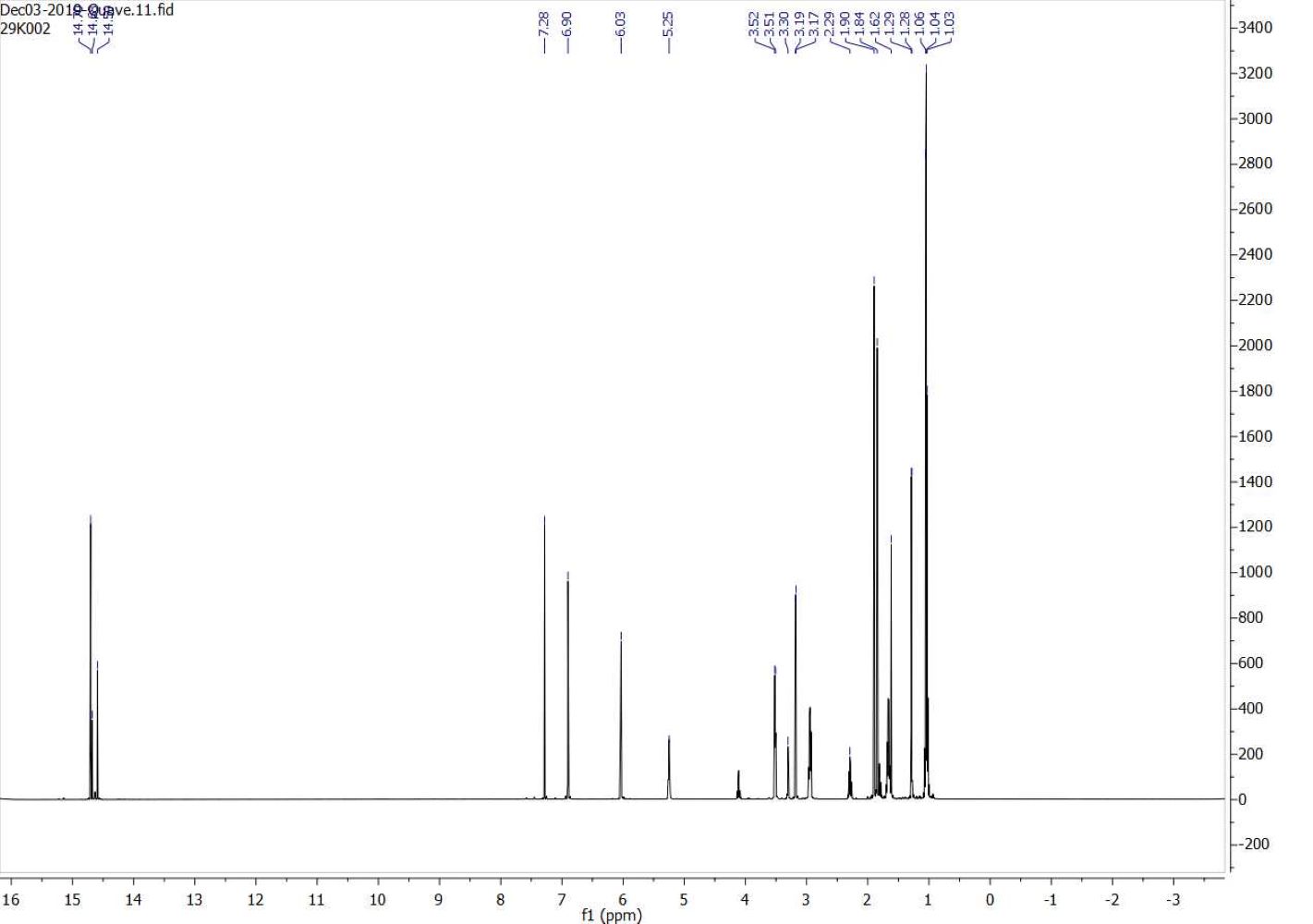

Supplement: Supplementary file 6 [file DataSheet1.zip › FIGURAS FRONTIERS/Figure S3.JPEG]

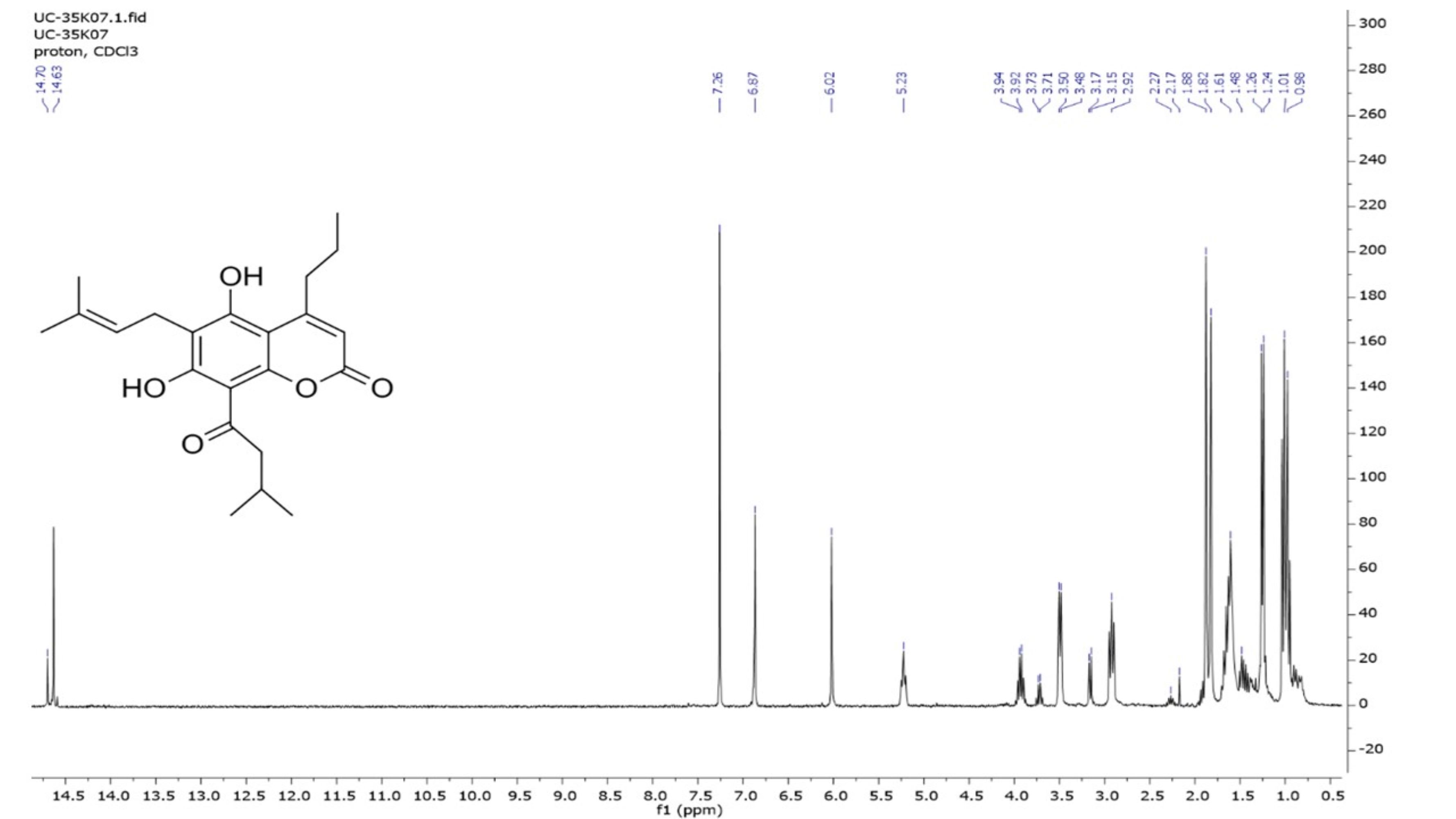

Supplement: Supplementary file 6 [file DataSheet1.zip › FIGURAS FRONTIERS/Figure S4.JPEG]

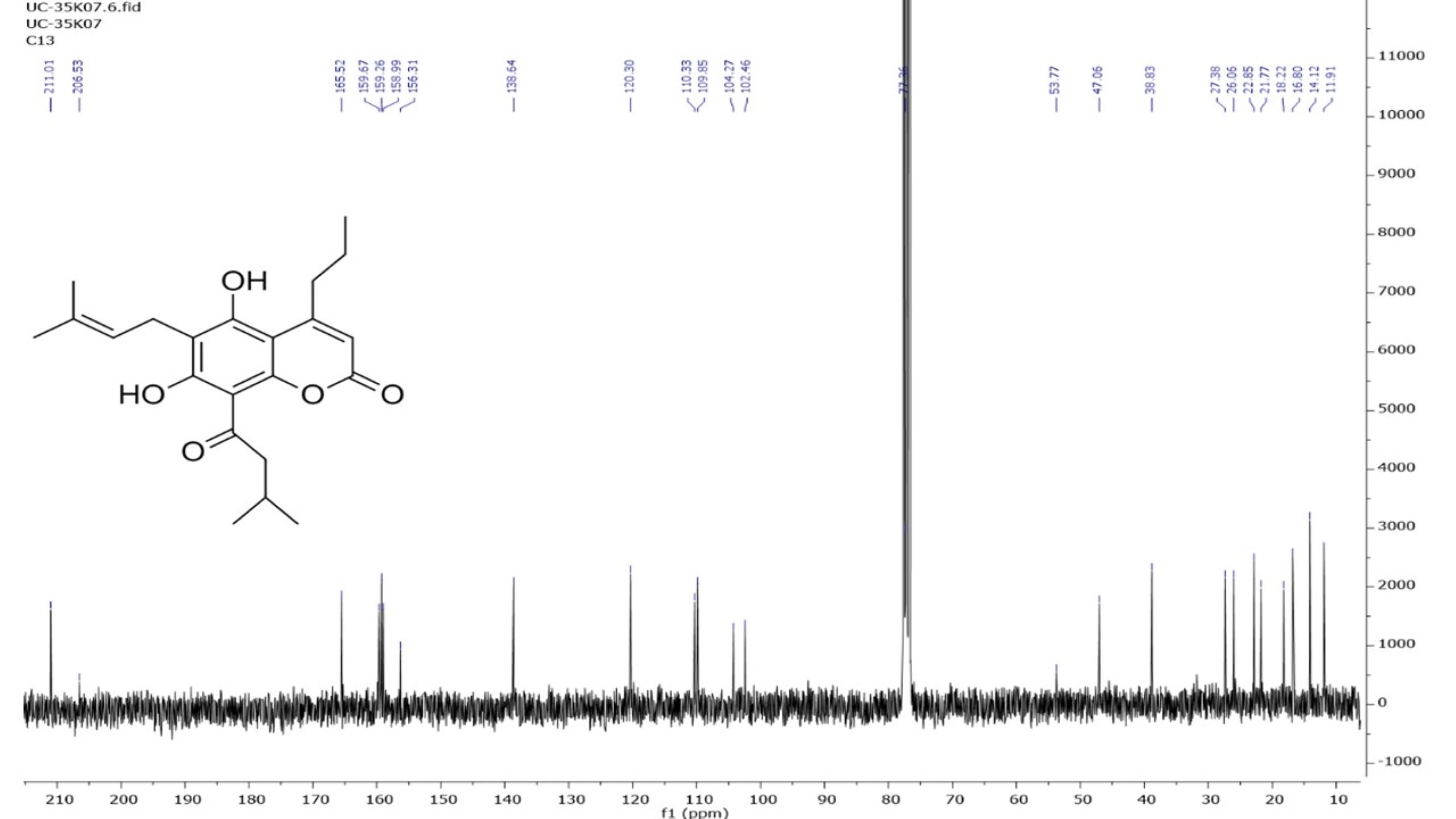

Supplement: Supplementary file 6 [file DataSheet1.zip › FIGURAS FRONTIERS/Figure S5.JPEG]

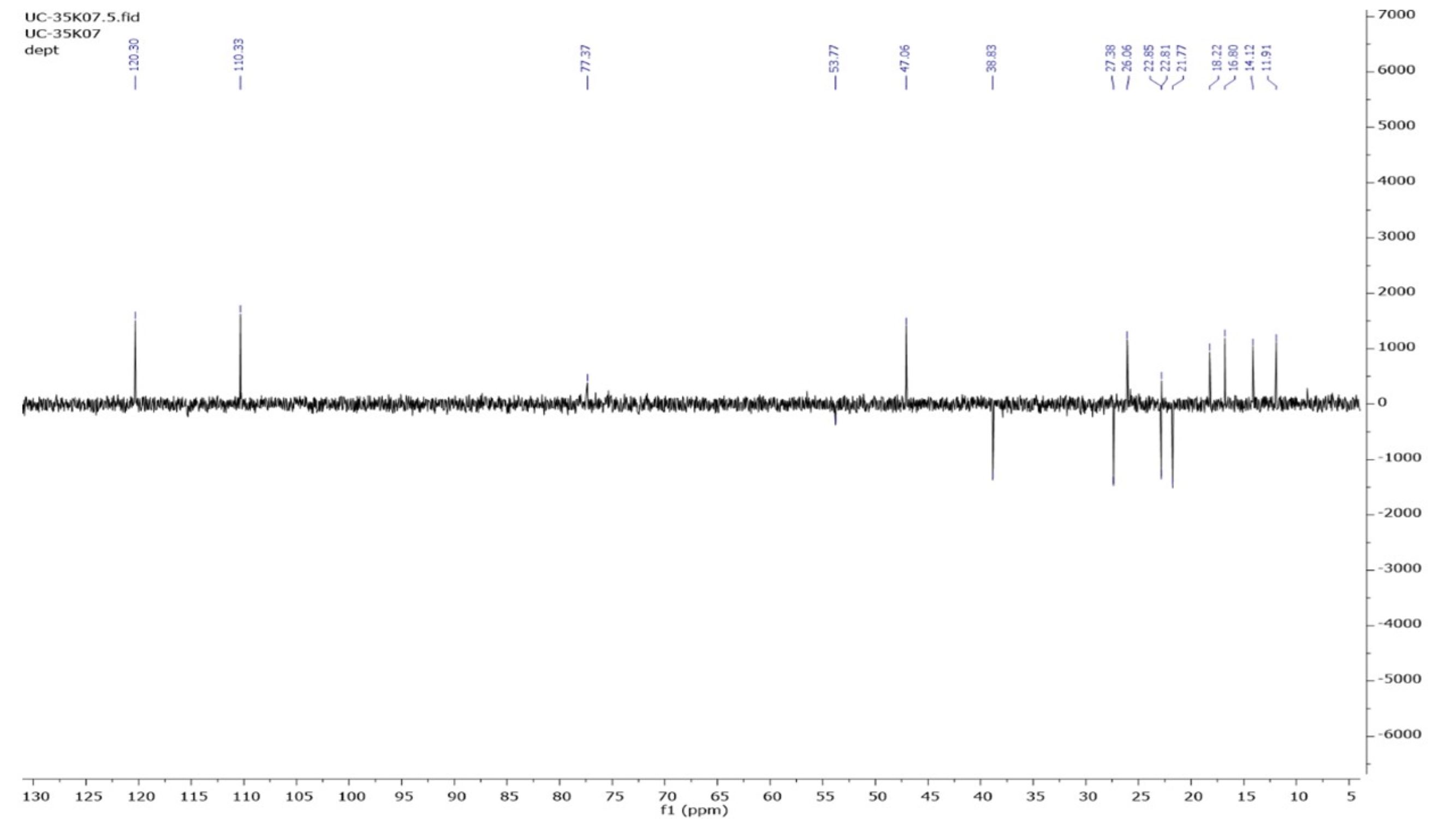

Supplement: Supplementary file 6 [file DataSheet1.zip › FIGURAS FRONTIERS/Figure S6.JPEG]

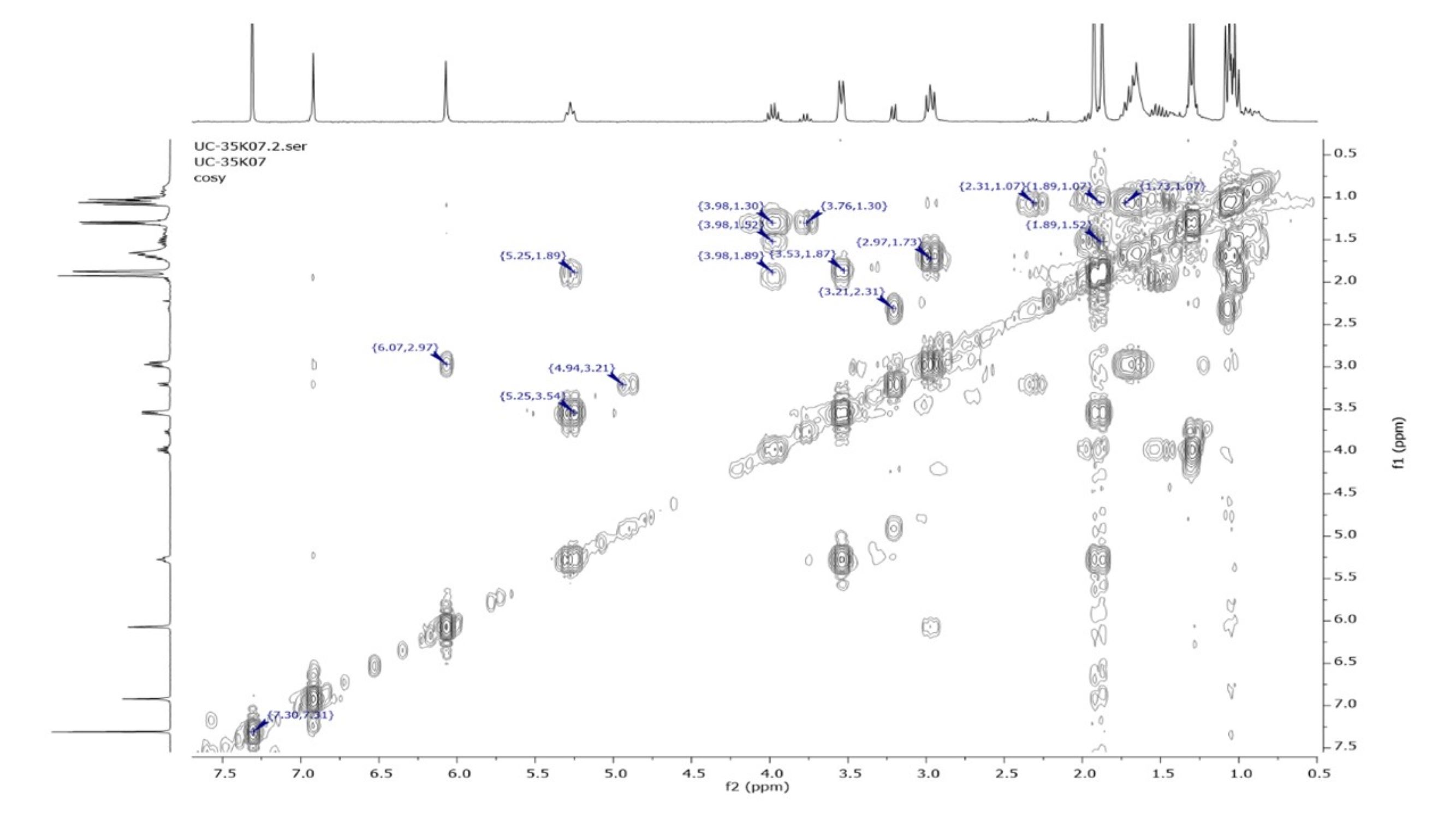

Supplement: Supplementary file 6 [file DataSheet1.zip › FIGURAS FRONTIERS/Figure S7.JPEG]

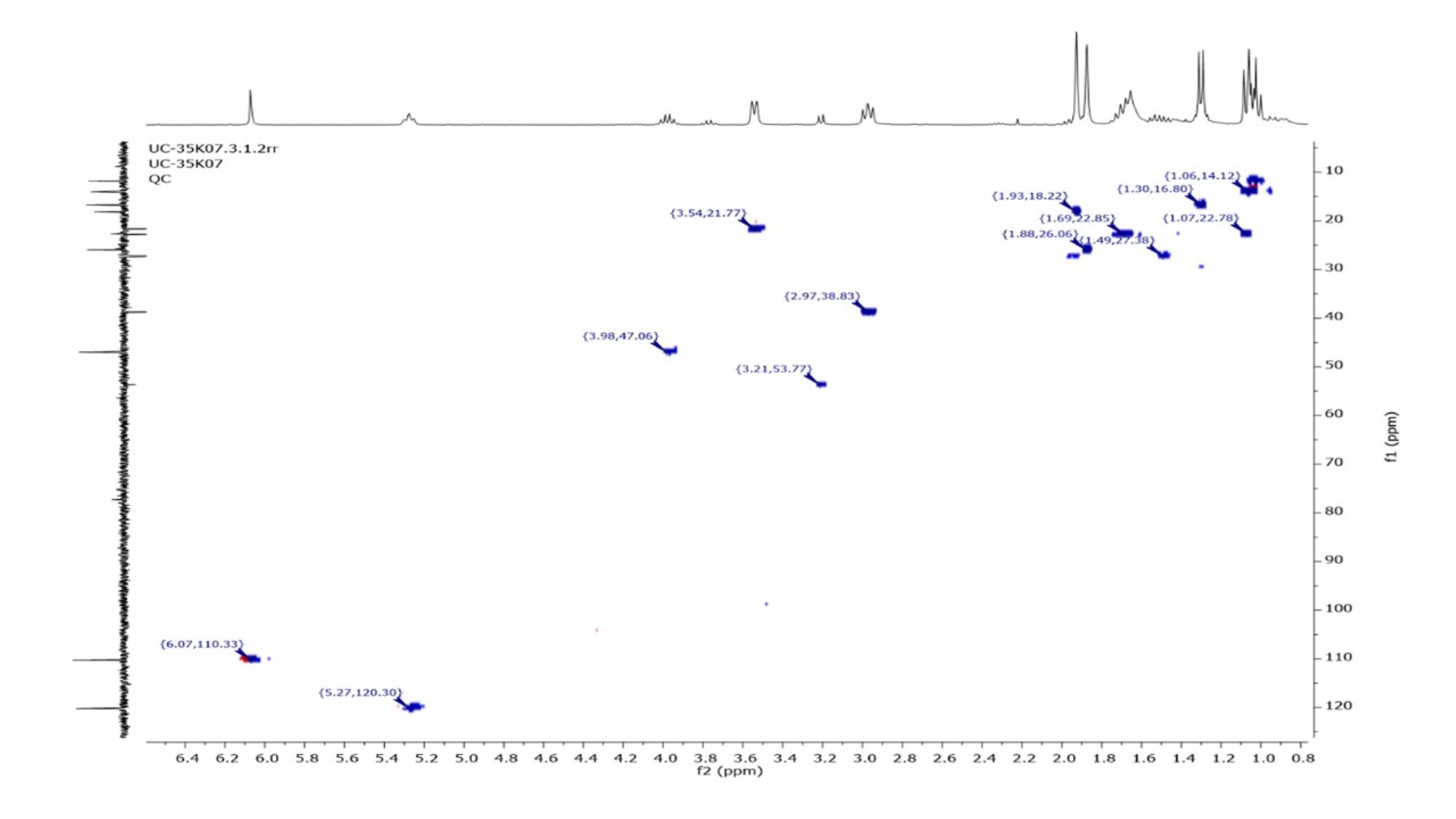

Supplement: Supplementary file 6 [file DataSheet1.zip › FIGURAS FRONTIERS/Figure S8.JPEG]

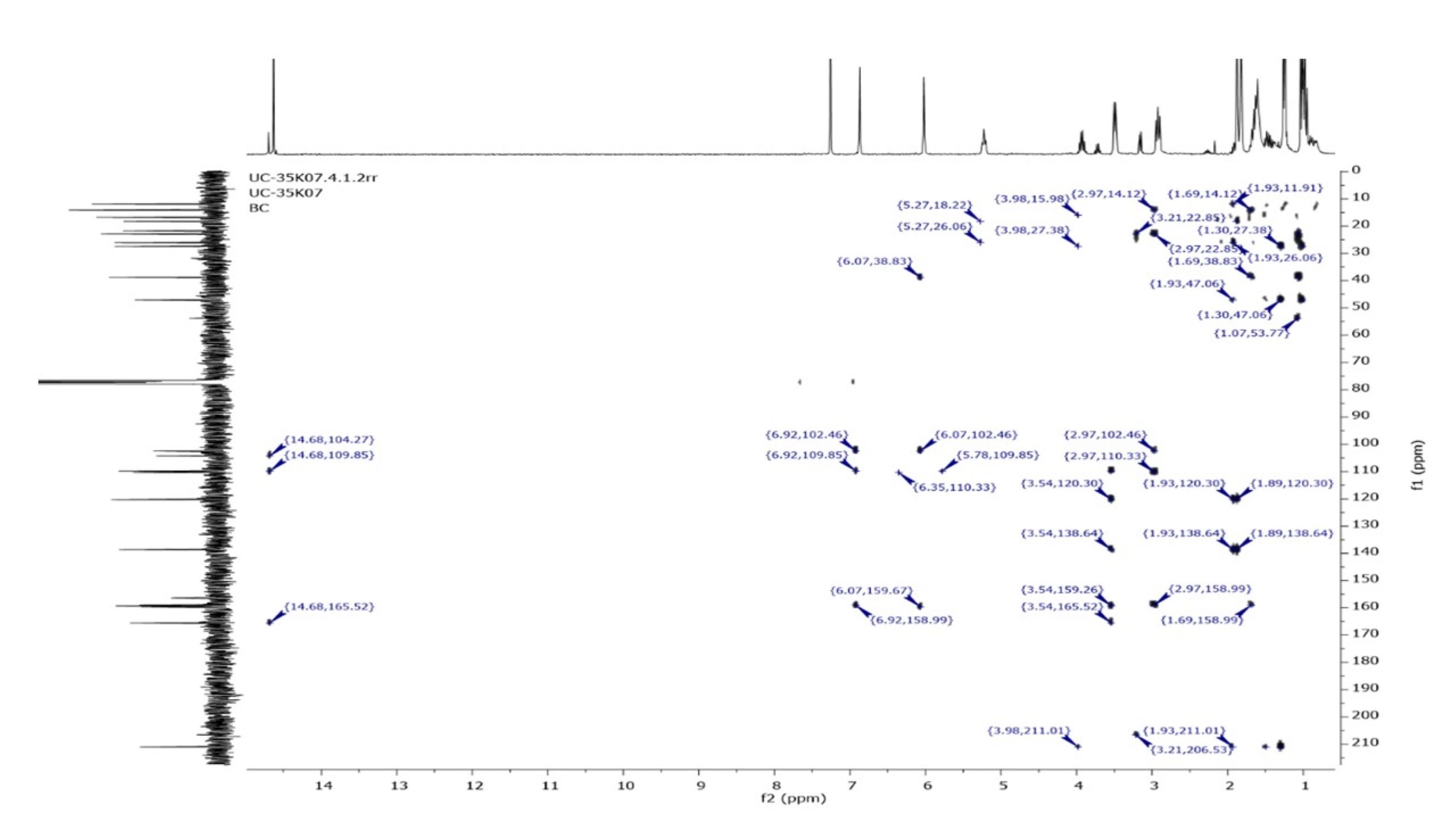

Supplement: Supplementary file 6 [file DataSheet1.zip › FIGURAS FRONTIERS/Figure S9.JPEG]
