# Supplementary material for: Mammea B/BA Isolated From the Seeds of Mammea americana L. (Calophyllaceae) is a Potent Inhibitor of Methicillin-Resistant Staphylococcus aureus
Source: Front Pharmacol. 2022 Mar 11;13:826404. doi: 10.3389/fphar.2022.826404 (PMC8961693; doi:10.3389/fphar.2022.826404)
Supplement: Supplementary file 3 [file Presentation1.pdf]

Supplementary Materials

Table S1: Characteristics of *S. aureus* strains isolated in a pediatric hospital in the city of Cartagena.

Code	Sensitive (S)/ Resistant (R)	Sampling site	Infection	Gen *mec*	Gen *pvl*	Gen *nuc*
Sau-2	R	Blood culture	Neonatal late sepsis	+	+	+
Sau-9	R	Blood culture	Staphylococcemia	+	+	+
Sau-11	S	Grow common germs	Sepsis of pulmonary origin	-	+	+
Sau-12	R	Blood culture	MRSA sepsis	+	+	+
Sau-17	R	Blood culture	Complicated staphylococcal disease	+	+	+
Sau-19	R	Blood culture	Skin abscess	+	+	+
Sau-25	S	Blood culture	Basal pneumonia	-	+	+
Sau-27	S	Foot discharge	Skin abscess	-	+	+
Sau-39	S	Abscess	Skin abscess	-	+	+
Sau-44	S	Blood culture	Unspecified pneumonia	-	+	+

Table S2: Minimum inhibitory concentration of the extract and fractions of the seeds of *Mammea americana* against reference strains of *Escherichia coli*, *Klebsiella pneumoniae* and *Pseudomonas aeruginosa*.

Extract /Fraction	*E. coli* ATCC 700603 (μg/mL)	*K. pneumonia* ATCC 27853 (μg/mL)	*P. aeruginosa* ATCC 25922 (μg/mL)
FD.I.34S	>32	>32	>32
34S.F01	>32	>32	>32
34S.F02	>32	>32	>32
34S.F05	>32	>32	>32
34S.F09	>32	>32	>32
34S.F10	>32	>32	>32
34S.F11	>32	>32	>32
34S.F12	>32	>32	>32
34S.F13	>32	>32	>32
34S.F14	>32	>32	>32
34S.F15	>32	>32	>32
34S.F06	>32	>32	>32
34S.F07	>32	>32	>32
34S.F08	>32	>32	>32
34S.F03	>32	>32	>32
34S.F04	>32	>32	>32

Table S3: Effect of *Mammea americana* ethanolic extract and MaBBA on biofilm formation of S. aureus USA300-0114

	***Mammea americana* ethanolic extract**	
**Concentration**	**% Biofilm inhibition±SD**	**% Growth inhibition±SD**
**1 (MIC/4)**	46.56±7.7	63.71±1.4
**0.5 (MIC/8)**	21.92±3.8	46.64±2.2
**0.25 (MIC/16)**	-0.35±1.5	25.03±3.4
**0.125 (MIC/32)**	4.5±3.2	21.1±3.9
**0.0625 (MIC/64)**	2.7±3.9	17.77±2.1
	**MaBBA**	
**Concentration**	**% Biofilm inhibition±SD**	**% Growth inhibition±SD**
**0.5 (MIC/2)**	55.46±6.6	58.49±0.9
**0.25 (MIC/4)**	43.01±1.9	34.44±1.4
**0.125 (MIC/8)**	6.17±1.3	26.68±2.4
**0.0625 (MIC/16)**	4.62±1.2	19.25±2.3
**0.0312 (MIC/32)**	4.21±3.1	4.86±7.2

Results are presented as mean±SD of triplicate samples from three independent assays (n=9).

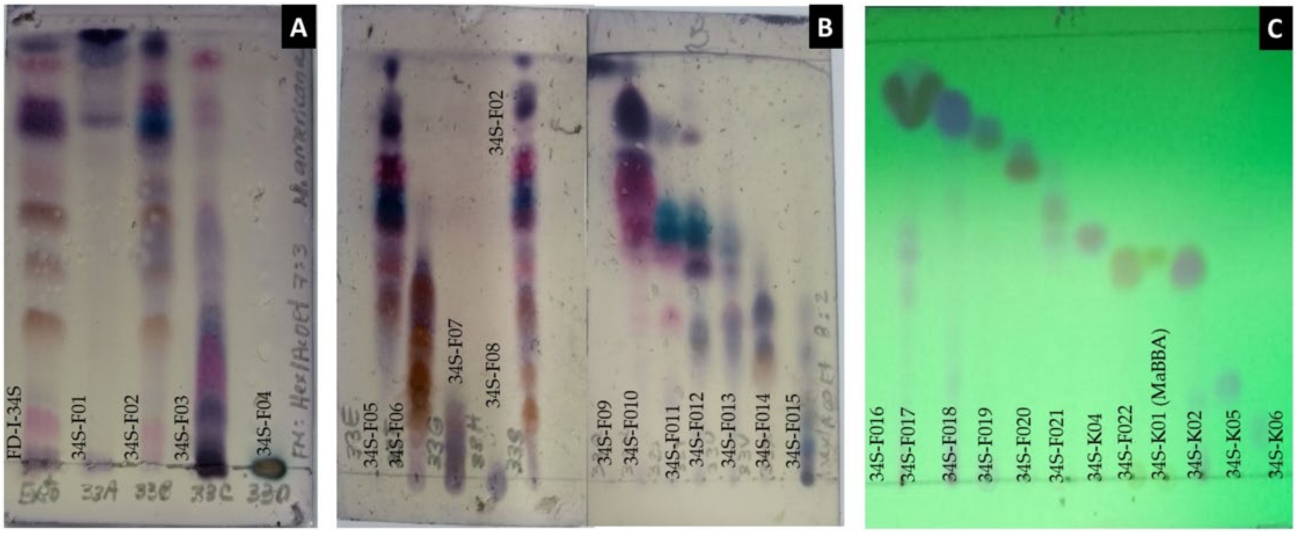

Figure S1: A. TLC of *M. americana* seed extract fractions (TLC: Merck Silica gel 60 F_{254}, Mobile phase: hexane / ethyl acetate 7: 3, Development: 1% vanillin); **B.** Subfractions (TLC: Merck Silica gel 60 F_{254}, Mobile phase: hexane/ethyl acetate 8:2, Development: 1% vanillin); **C.** Subfractions and compounds (TLC: Silica gel 60 F_{254} Merck, Mobile phase: hexane/ethyl acetate 8:2, Development: UV light 254 nm).

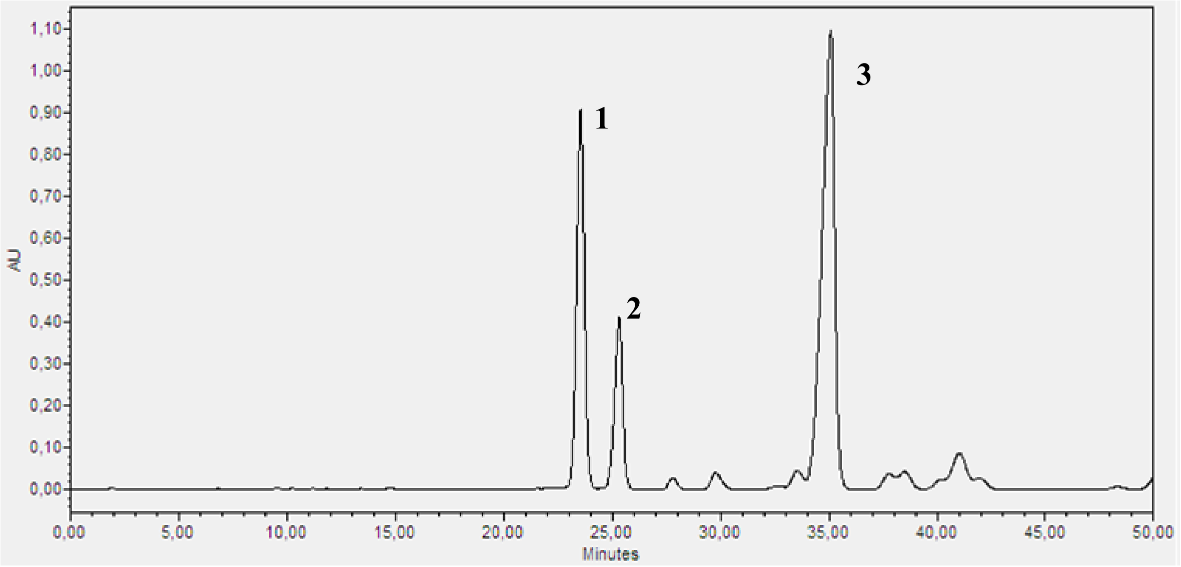

Figure S2: Reverse phase HPLC chromatogram of crystalline fraction (34S-F22). Conditions: acetonitrile/acetic acid 0.1% (7:3), flow 1.0 mL/min, 254 nm. **1:** Mammea B/BC, **2:** Not identified, **3:** Mammea B/BA.

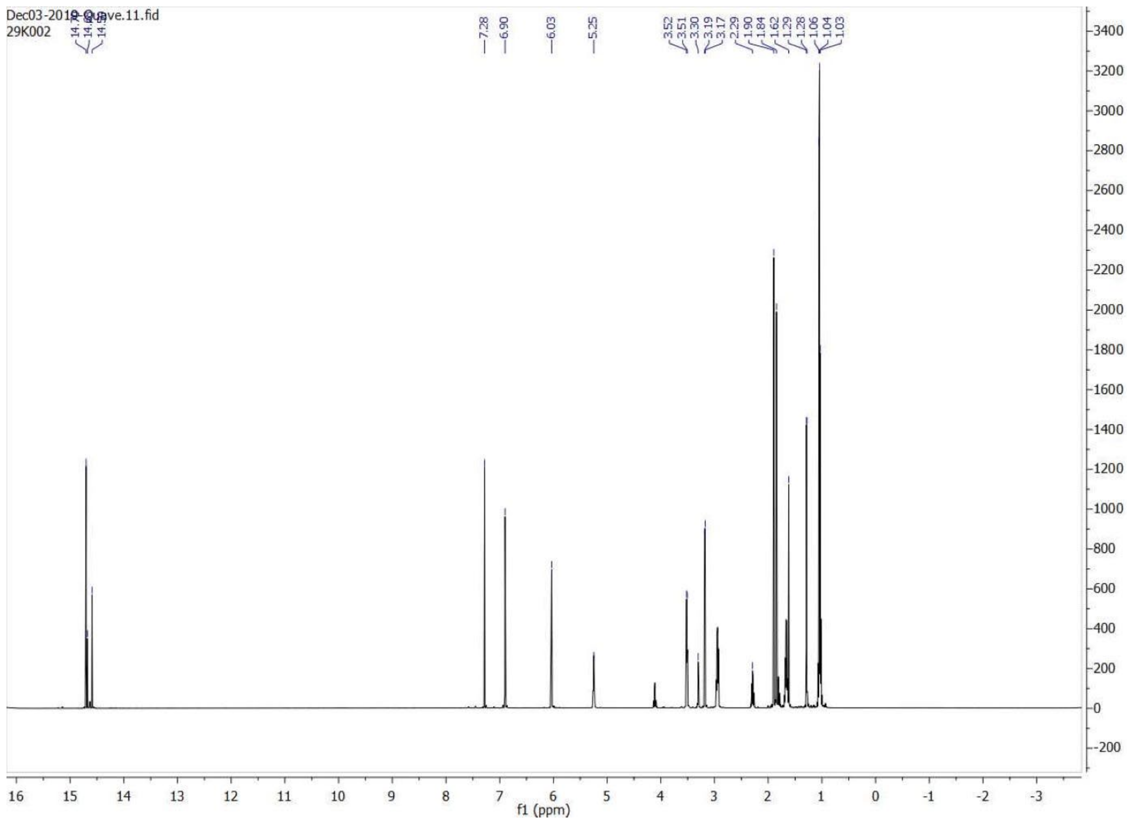

Figure S3: ^{1}H-NMR spectrum of the crystalline fraction (34S-F22).

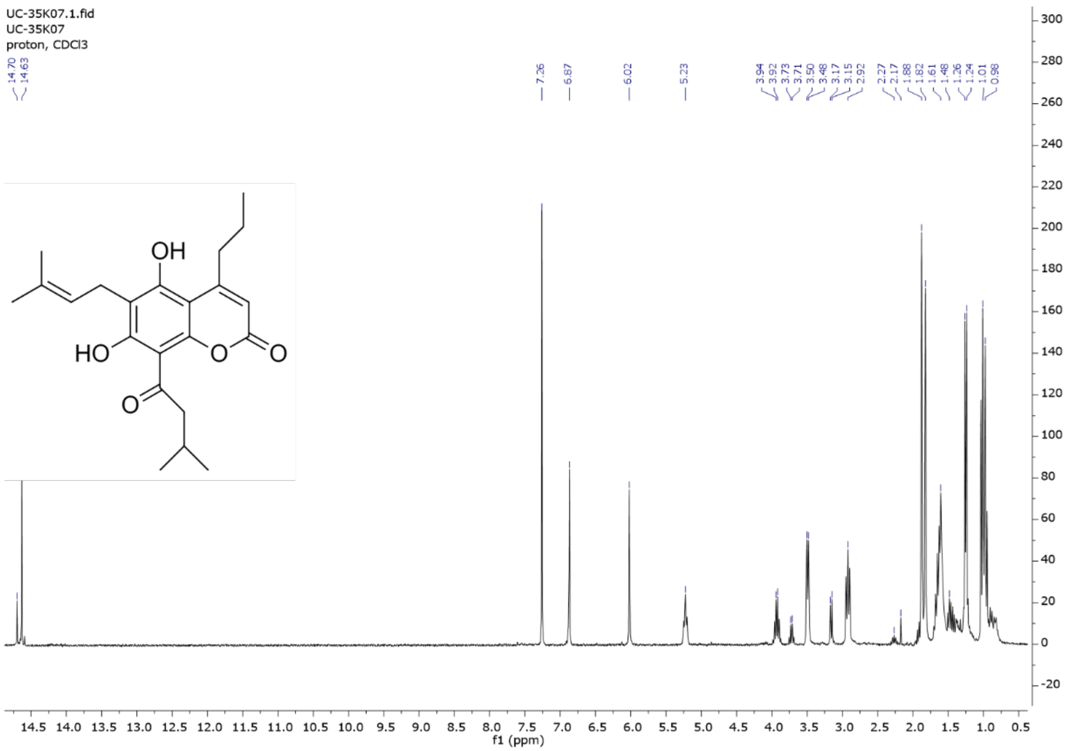

Figure S4: ^{1}H spectrum of Mammea B/BA.

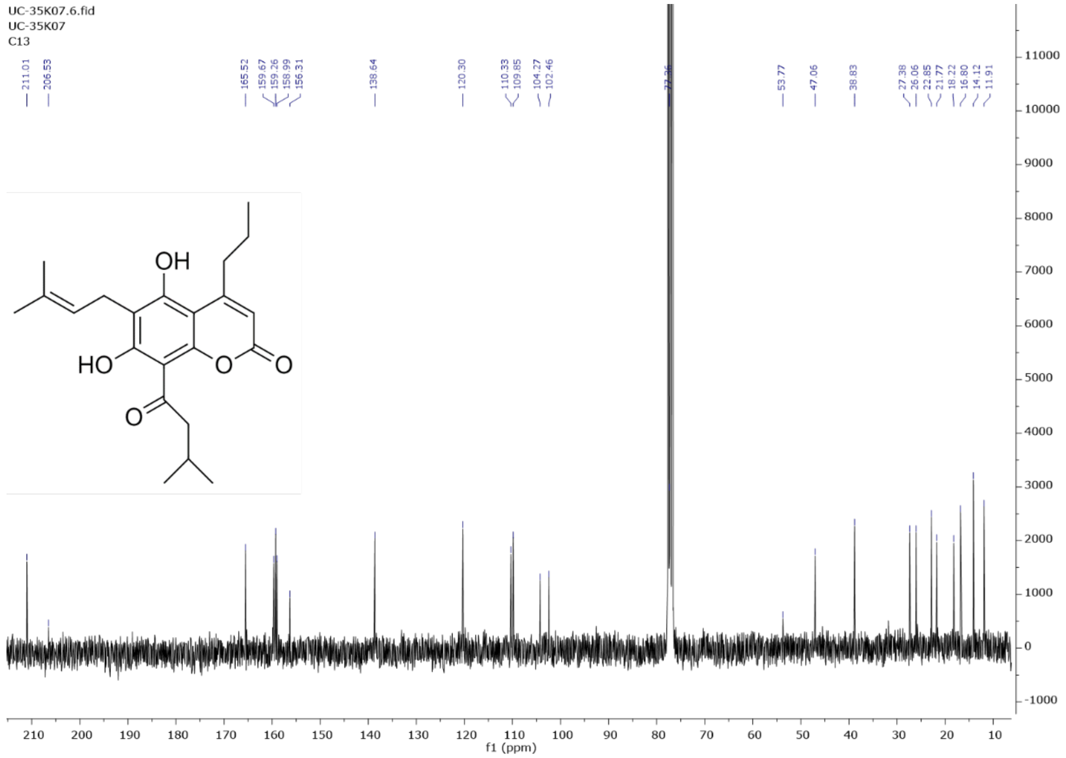

Figure S5: ^{13}C spectrum of Mammea B/BA.

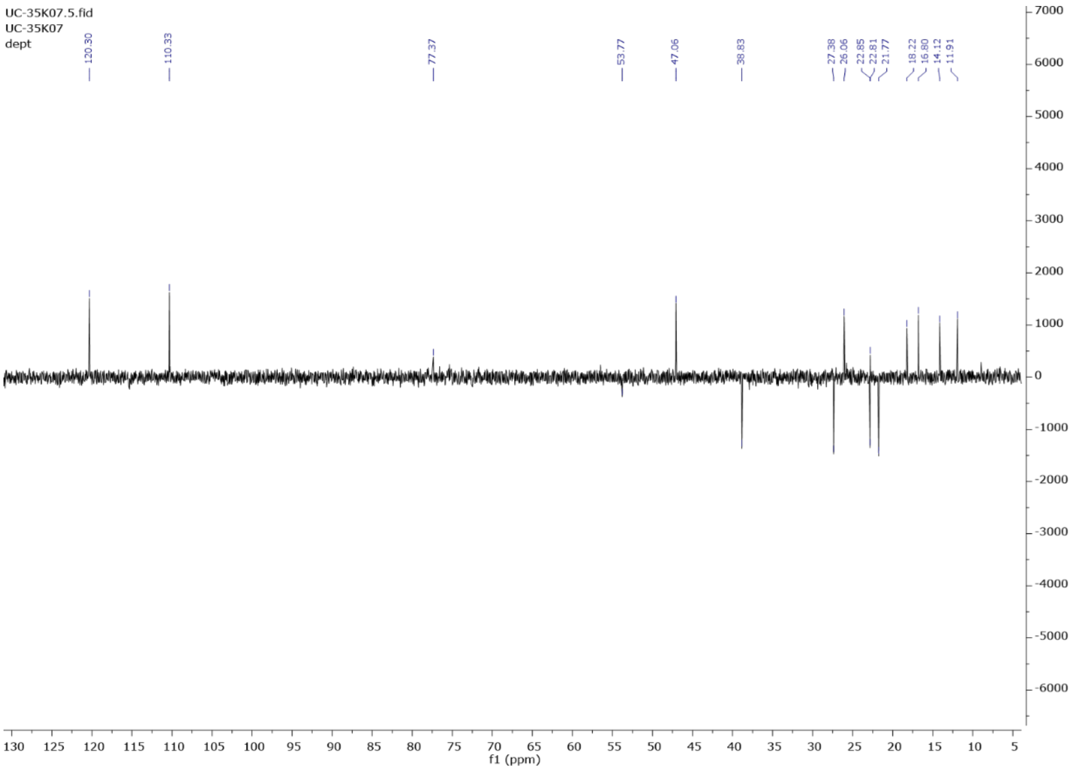

Figure S6: DEPT spectrum of Mammea B/BA.

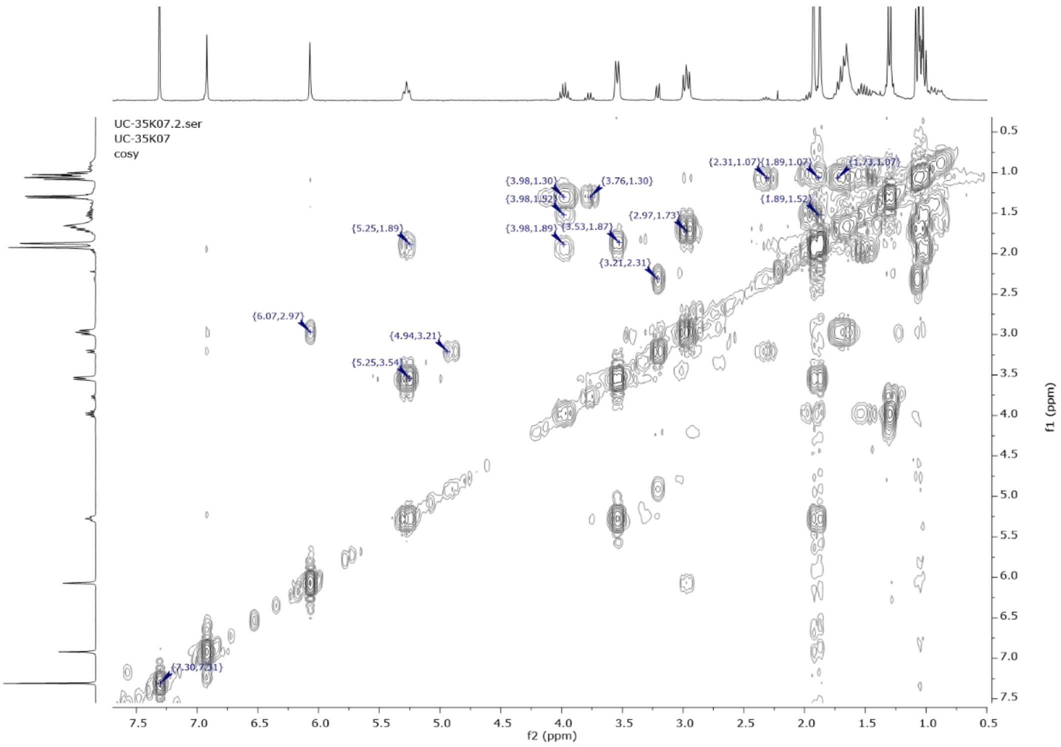

Figure S7: COSY spectrum of Mammea B/BA.

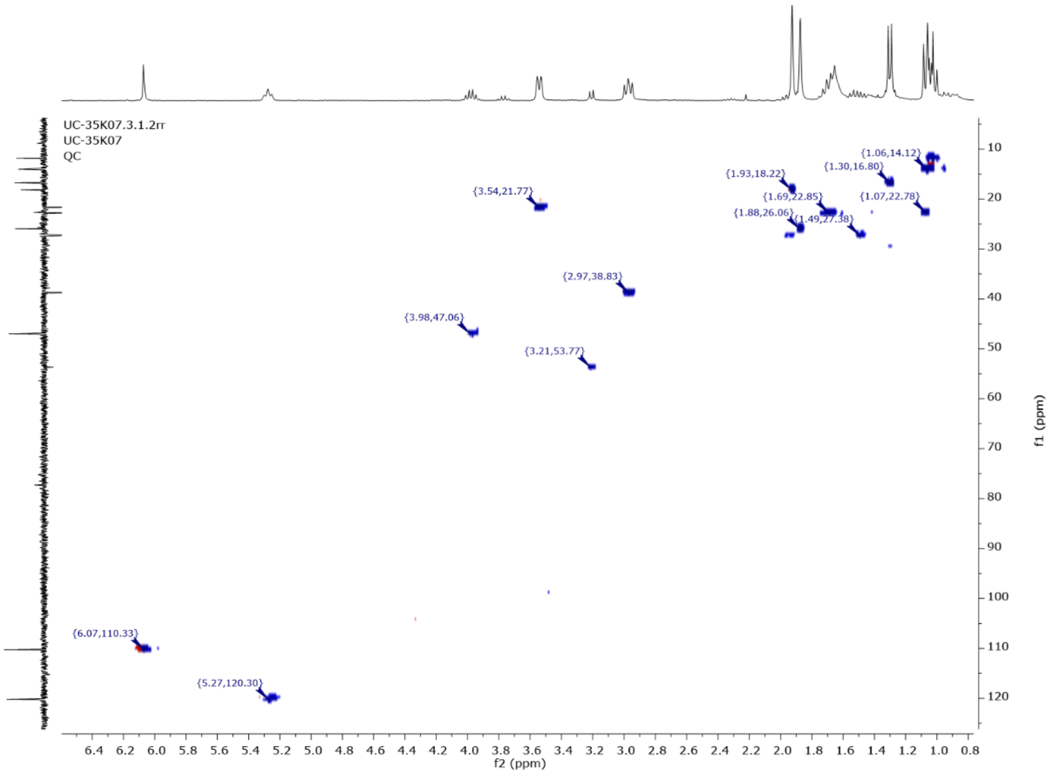

Figure S8: HSQC spectrum of Mammea B/BA.

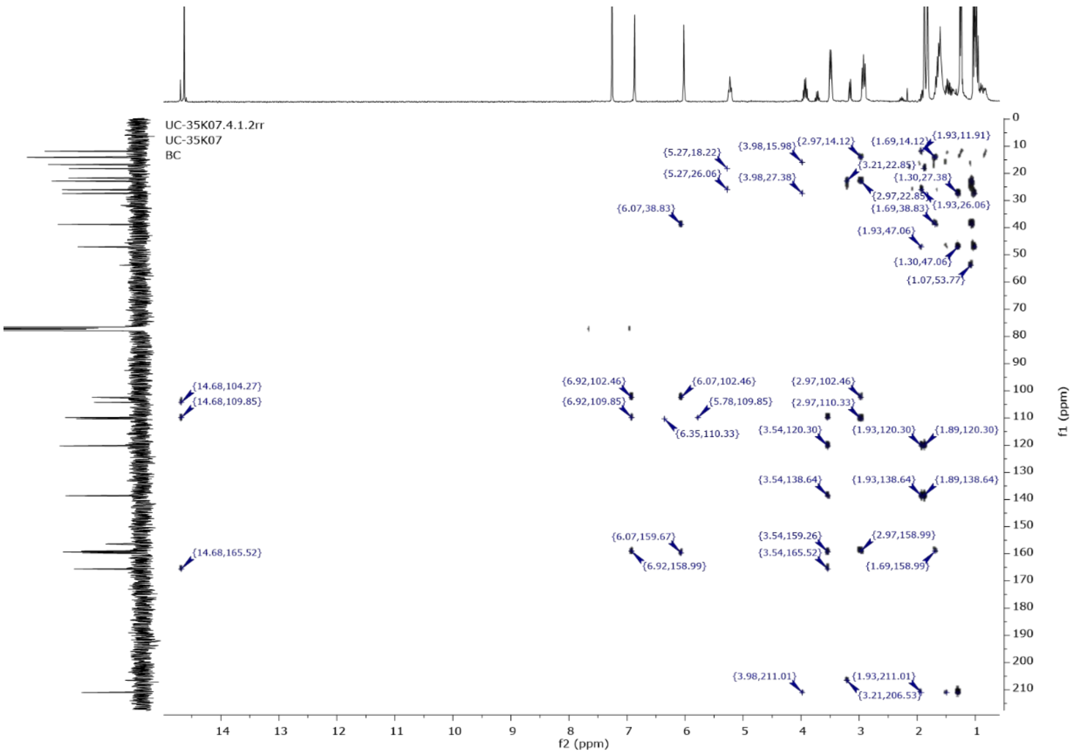

Figure S9: HMBC spectrum of Mammea B/BA.

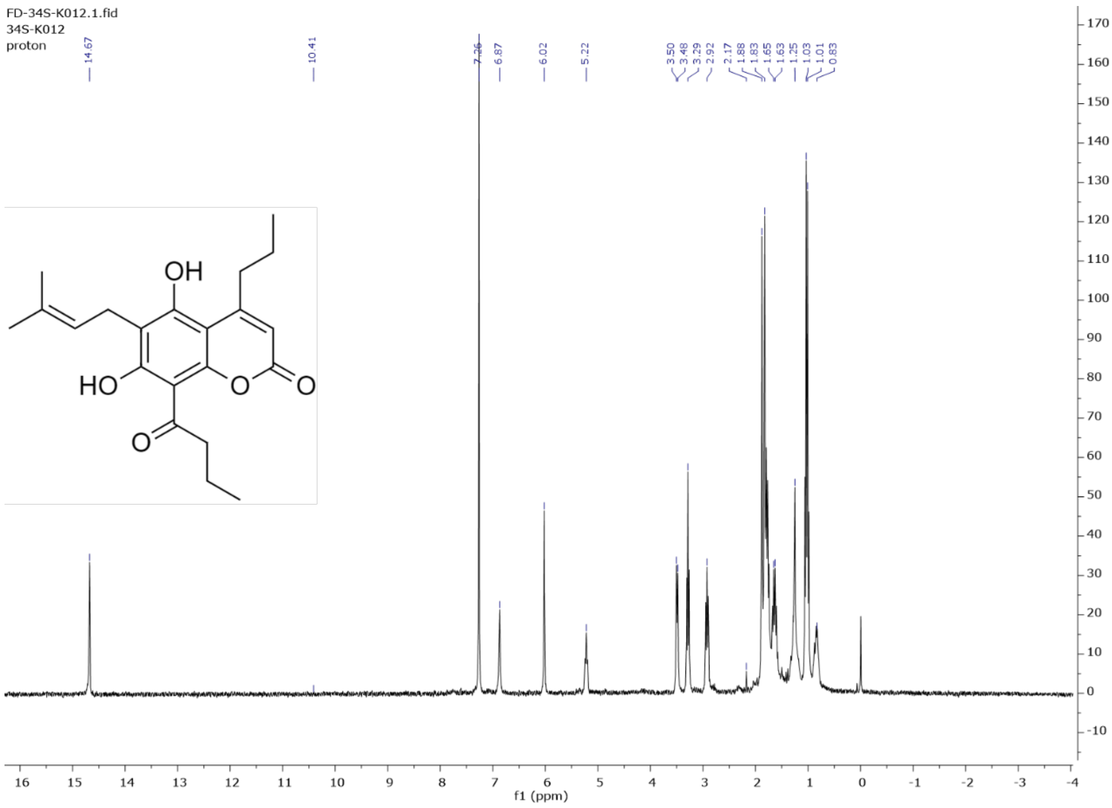

Figure S10: ^{1}H spectrum of Mammea B/BC.

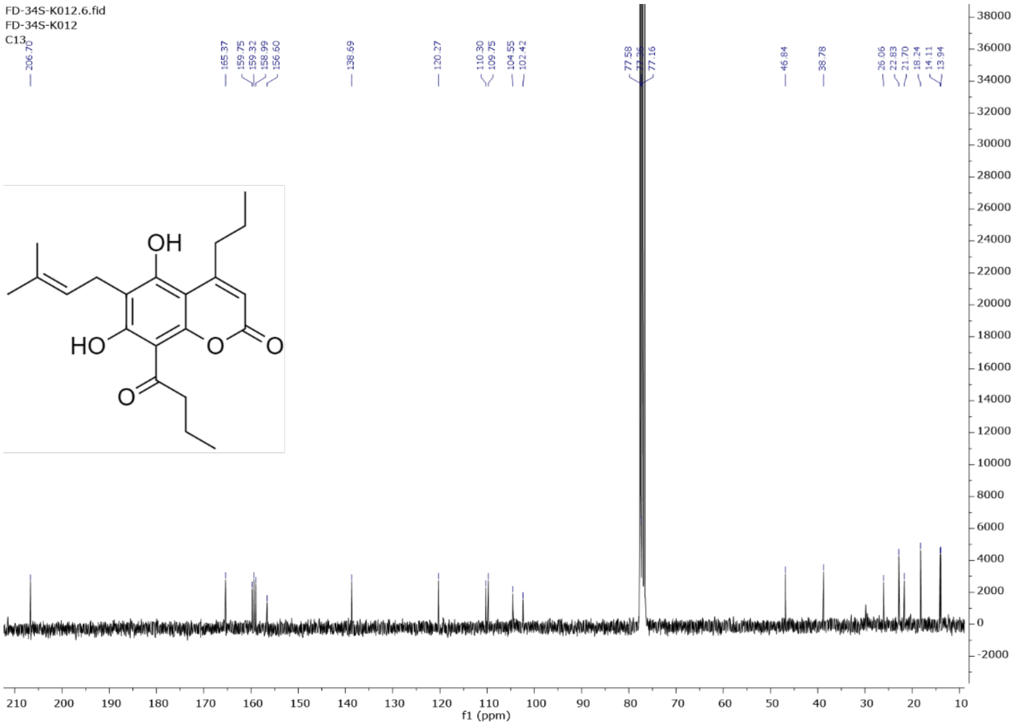

Figure S11: ^{13}C spectrum of Mammea B/BC.

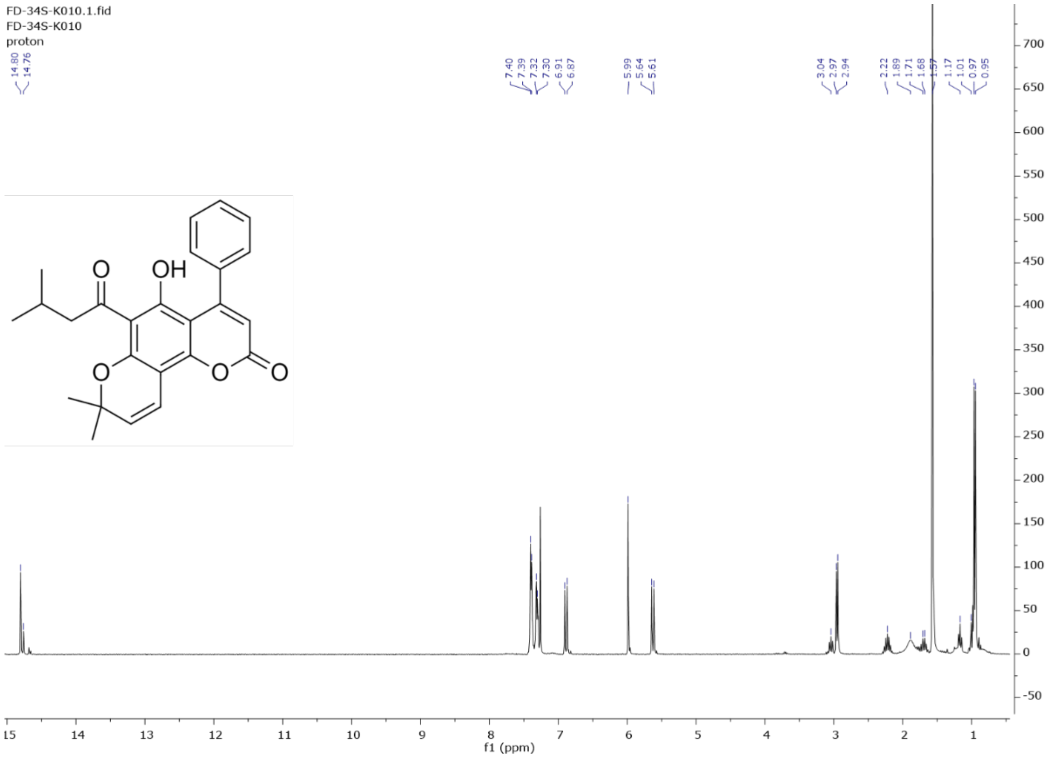

Figure S12: ^{1}H spectrum of Mammea A/AA cycle D.

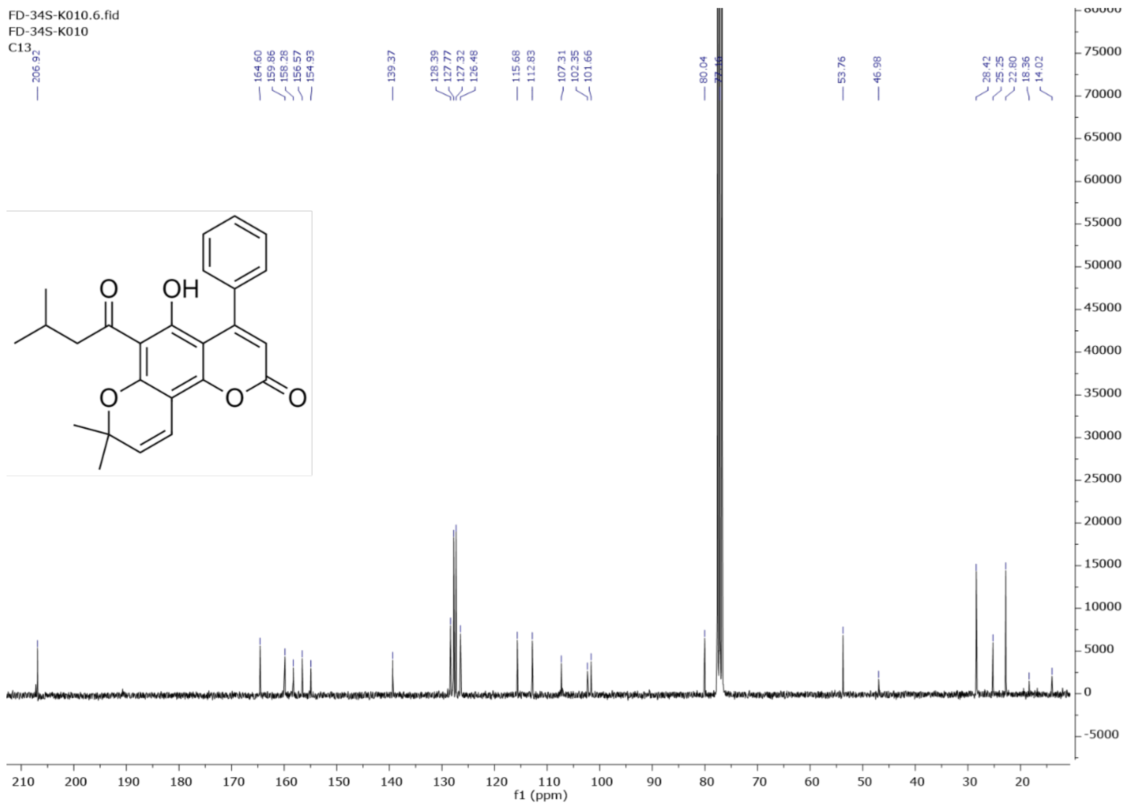

Figure S13: ^{13}C spectrum of Mammea A/AA cycle D.

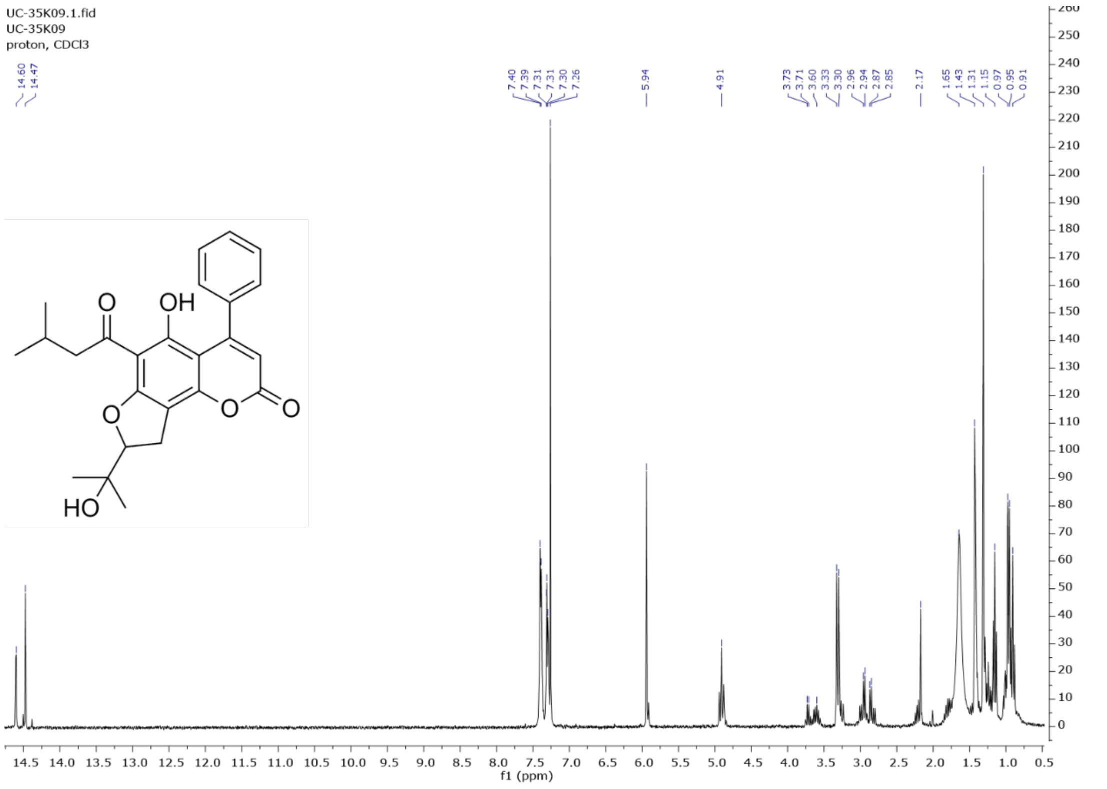

Figure S14: ^{1}H spectrum of Mammea A/AA cycle F.

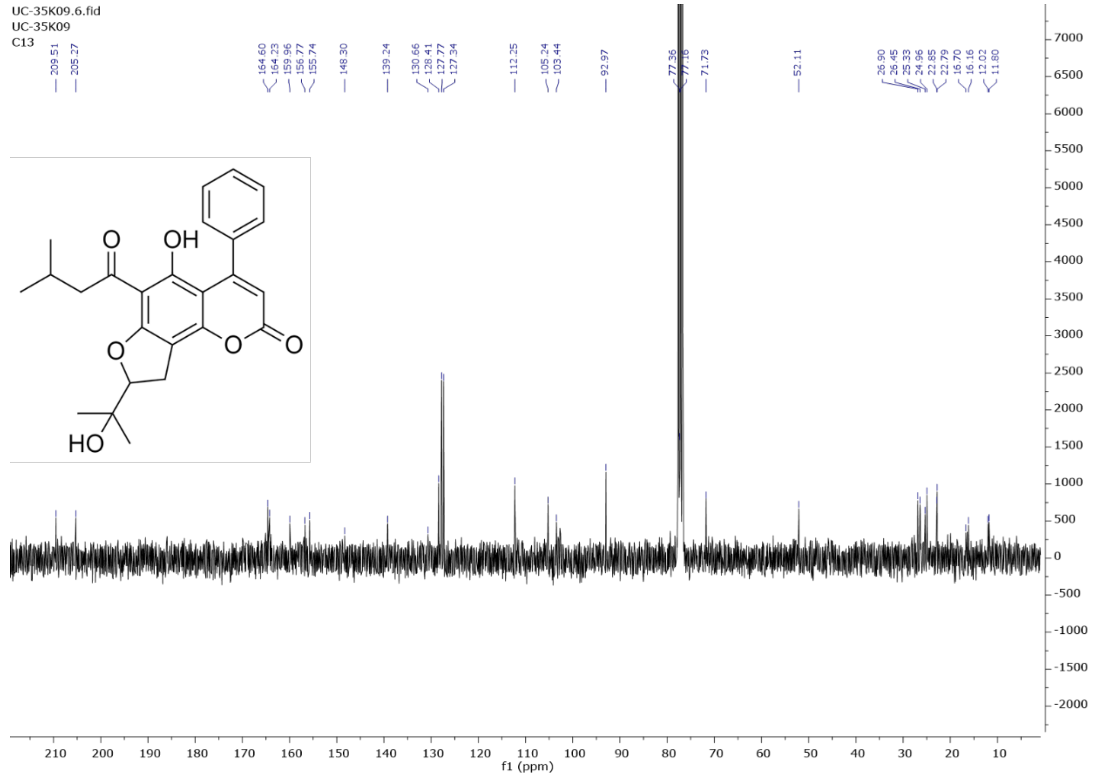

Figure S15: ^{13}C spectrum of Mammea A/AA cycle F.

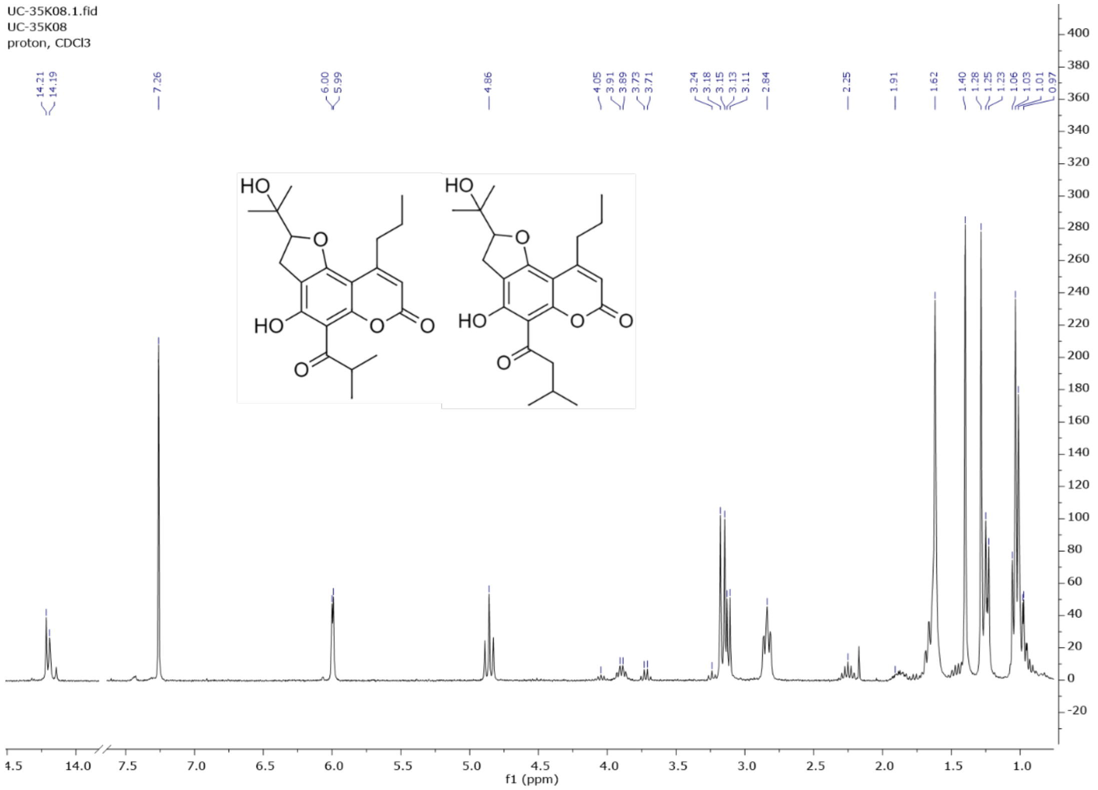

Figure S16: ^{1}H spectrum of mixture of Mammea B/BA cycle F and Mammea B/BD cycle F.

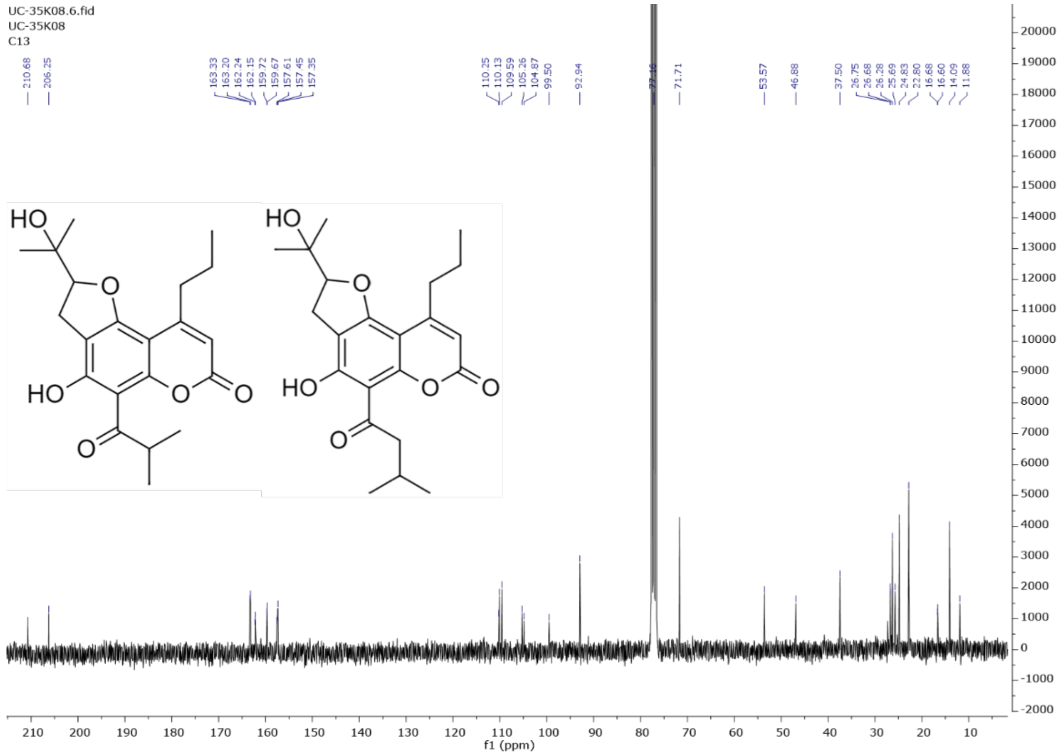

Figure S17: ^{13}C spectrum of mixture of Mammea B/BA cycle F and Mammea B/BD cycle F.

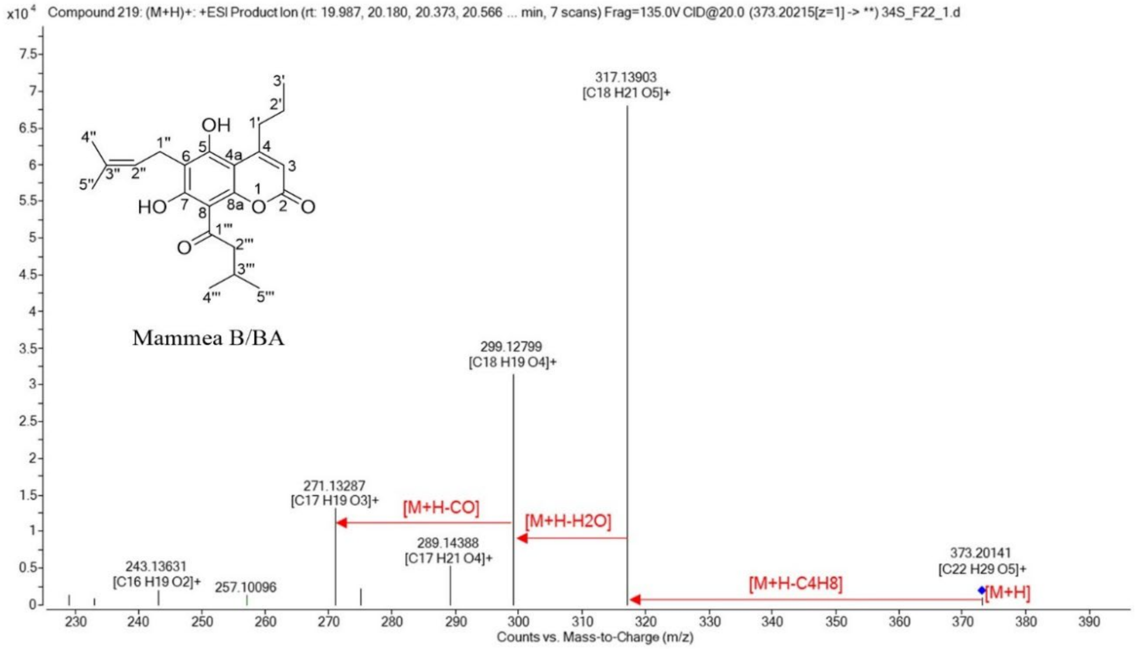

Figure S18. ESI-MS spectrum of Mammea B/BA corresponding to the molecular formula: $C_{22}H_{28}O_5$, with a protonated molecular ion at *m/z* 373,20141 [M+H] and a Retention Time (RT) of 20.34 minutes.

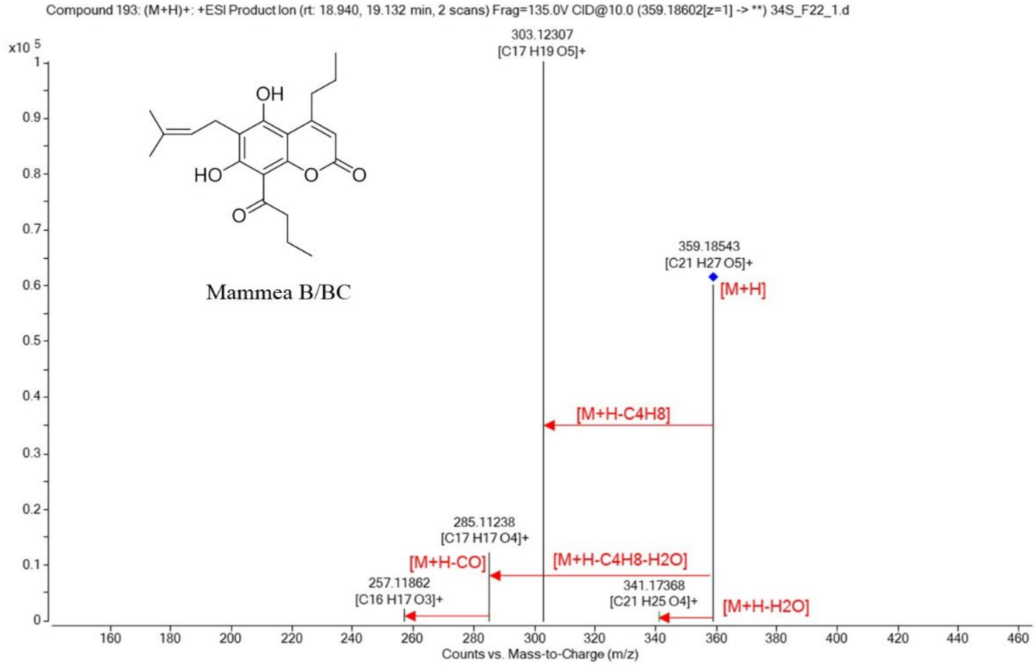

Figure S19. ESI-MS spectrum of Mammea B/BC corresponding to the molecular formula: $C_{21}H_{26}O_5$, with a protonated molecular ion at *m/z* 359,18543 [M+H] and a Retention Time (RT) of 19.06 minutes.

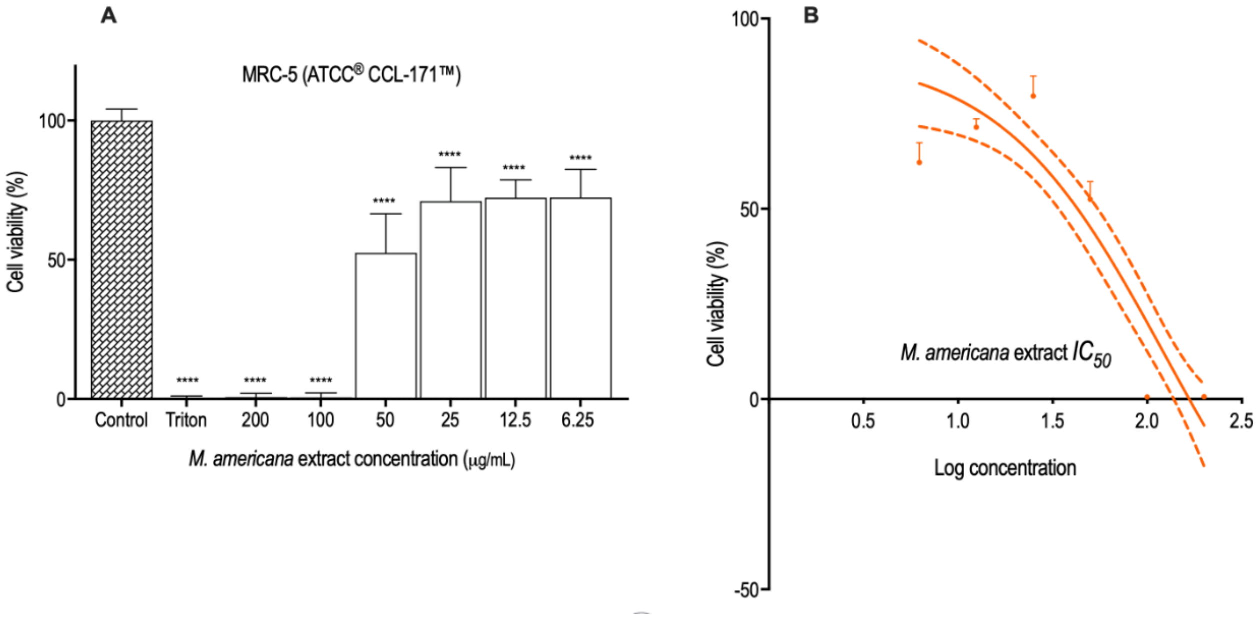

Figure S20. Cytotoxicity of *M. americana* total ethanolic extract against MRC-5 cell line. (A) Effect of different concentrations of the extract on cell growth. (b) Non-linear regression and IC_{50} calculate. Results are presented as mean± SEM of triplicate samples from three independent assays (n=9). Significant differences from growth control are indicated by ****P<0.0001.
