# Supplementary material for: Mammea B/BA Isolated From the Seeds of Mammea americana L. (Calophyllaceae) is a Potent Inhibitor of Methicillin-Resistant Staphylococcus aureus
Source: Front Pharmacol. 2022 Mar 11;13:826404. doi: 10.3389/fphar.2022.826404 (PMC8961693; doi:10.3389/fphar.2022.826404)
Supplement: Supplementary file 6 [file DataSheet1.zip › FIGURAS FRONTIERS/Table 1.DOCX]

**Supplementary Materials**

**Table S1:** Characteristics of *S. aureus* strains isolated in a pediatric hospital in the city of Cartagena.

| **Code** | **Sensitive (S)/**  **Resistant (R)** | **Sampling site** | **Infection** | **Gen *mec*** | **Gen *pvl*** | **Gen *nuc*** |
| --- | --- | --- | --- | --- | --- | --- |
| Sau-2 | R | Blood culture | Neonatal late sepsis | + | + | + |
| Sau-9 | R | Blood culture | Staphylococcemia | + | + | + |
| Sau-11 | S | Grow common germs | Sepsis of pulmonary origin | - | + | + |
| Sau-12 | R | Blood culture | MRSA sepsis | + | + | + |
| Sau-17 | R | Blood culture | Complicated staphylococcal disease | + | + | + |
| Sau-19 | R | Blood culture | Skin abscess | + | + | + |
| Sau-25 | S | Blood culture | Basal pneumonia | - | + | + |
| Sau-27 | S | Foot discharge | Skin abscess | - | + | + |
| Sau-39 | S | Abscess | Skin abscess | - | + | + |
| Sau-44 | S | Blood culture | Unspecified pneumonia | - | + | + |

**Table S2:** Minimum inhibitory concentration of the extract and fractions of the seeds of *Mammea americana* against reference strains of *Escherichia coli*, *Klebsiella pneumoniae* and *Pseudomonas aeruginosa*.

| **Extract /Fraction** | ***E. coli***  **ATCC 700603**  **(µg/mL)** | ***K. pneumonia***  **ATCC 27853**  **(µg/mL)** | ***P. aeruginosa***  **ATCC 25922**  **(µg/mL)** |
| --- | --- | --- | --- |
| FD.I.34S | >32 | >32 | >32 |
| 34S.F01 | >32 | >32 | >32 |
| 34S.F02 | >32 | >32 | >32 |
| 34S.F05 | >32 | >32 | >32 |
| 34S.F09 | >32 | >32 | >32 |
| 34S.F10 | >32 | >32 | >32 |
| 34S.F11 | >32 | >32 | >32 |
| 34S.F12 | >32 | >32 | >32 |
| 34S.F13 | >32 | >32 | >32 |
| 34S.F14 | >32 | >32 | >32 |
| 34S.F15 | >32 | >32 | >32 |
| 34S.F06 | >32 | >32 | >32 |
| 34S.F07 | >32 | >32 | >32 |
| 34S.F08 | >32 | >32 | >32 |
| 34S.F03 | >32 | >32 | >32 |
| 34S.F04 | >32 | >32 | >32 |

**Table S3:** Effect of *Mammea americana* ethanolic extract and MaBBA on biofilm formation of S. aureus USA300-0114

| ***Mammea americana* ethanolic extract** | | |
| --- | --- | --- |
| **Concentration** | **% Biofilm inhibition±SD** | **% Growth inhibition±SD** |
| **1 (MIC/4)** | 46.56±7.7 | 63.71±1.4 |
| **0.5 (MIC/8)** | 21.92±3.8 | 46.64±2.2 |
| **0.25 (MIC/16)** | -0.35±1.5 | 25.03±3.4 |
| **0.125 (MIC/32)** | 4.5±3.2 | 21.1±3.9 |
| **0.0625 (MIC/64)** | 2.7±3.9 | 17.77±2.1 |
| **MaBBA** | | |
| **Concentration** | **% Biofilm inhibition±SD** | **% Growth inhibition±SD** |
| **0.5 (MIC/2)** | 55.46±6.6 | 58.49±0.9 |
| **0.25 (MIC/4)** | 43.01±1.9 | 34.44±1.4 |
| **0.125 (MIC/8)** | 6.17±1.3 | 26.68±2.4 |
| **0.0625 (MIC/16)** | 4.62±1.2 | 19.25±2.3 |
| **0.0312 (MIC/32)** | 4.21±3.1 | 4.86±7.2 |

Results are presented as mean±SD of triplicate samples from three independent assays (n=9).


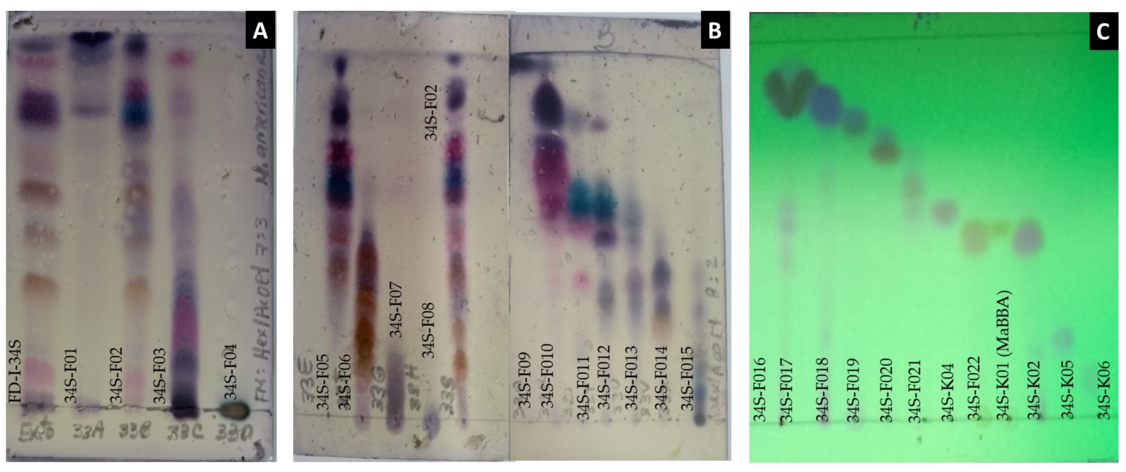


**Figure S1: A.** TLC of *M. americana* seed extract fractions (TLC: Merck Silica gel 60 F_254_, Mobile phase: hexane / ethyl acetate 7: 3, Development: 1% vanillin); **B.** Subfractions (TLC: Merck Silica gel 60 F_254_, Mobile phase: hexane/ethyl acetate 8:2, Development: 1% vanillin); **C.** Subfractions and compounds (TLC: Silica gel 60 F_254_ Merck, Mobile phase: hexane/ethyl acetate 8:2, Development: UV light 254 nm).


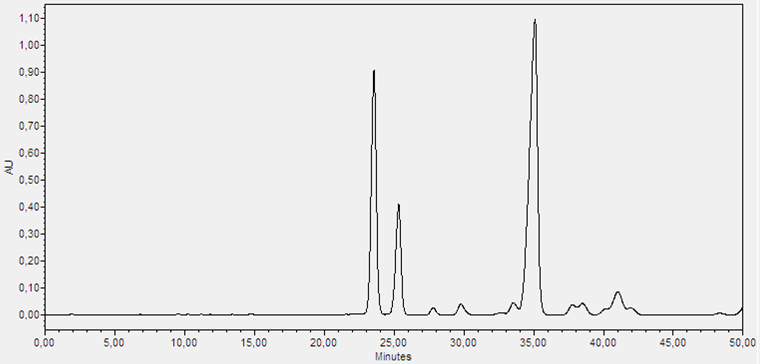


**3**

**2**

**1**

**Figure S2:** Reverse phase HPLC chromatogram of crystalline fraction (34S-F22). Conditions: acetonitrile/acetic acid 0.1% (7:3), flow 1.0 mL/min, 254 nm. **1:** Mammea B/BC, **2:** Not identified, **3:** Mammea B/BA.


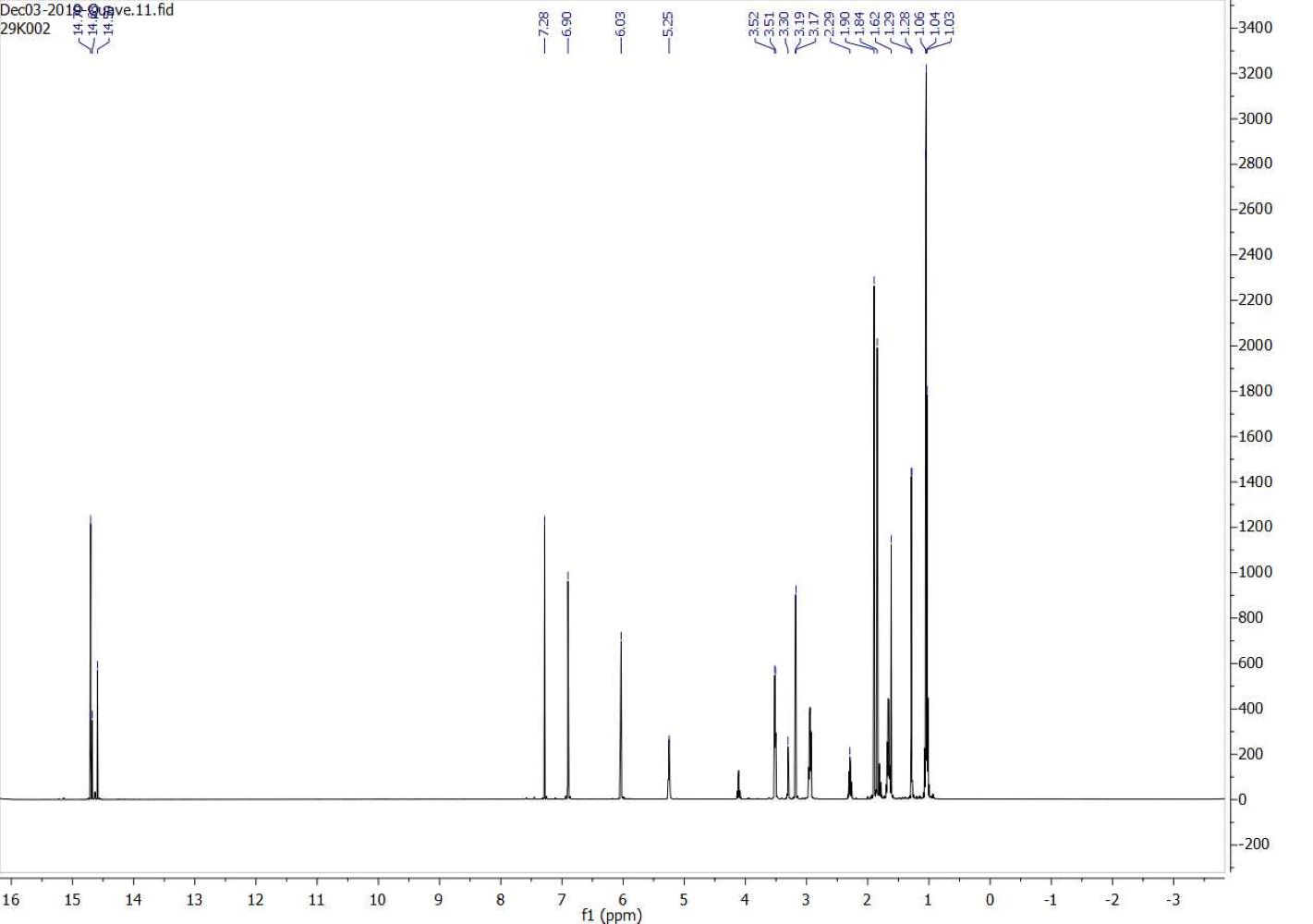


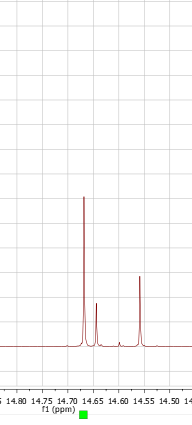
**Figure S3:** ^1^H-NMR spectrum of the crystalline fraction (34S-F22).


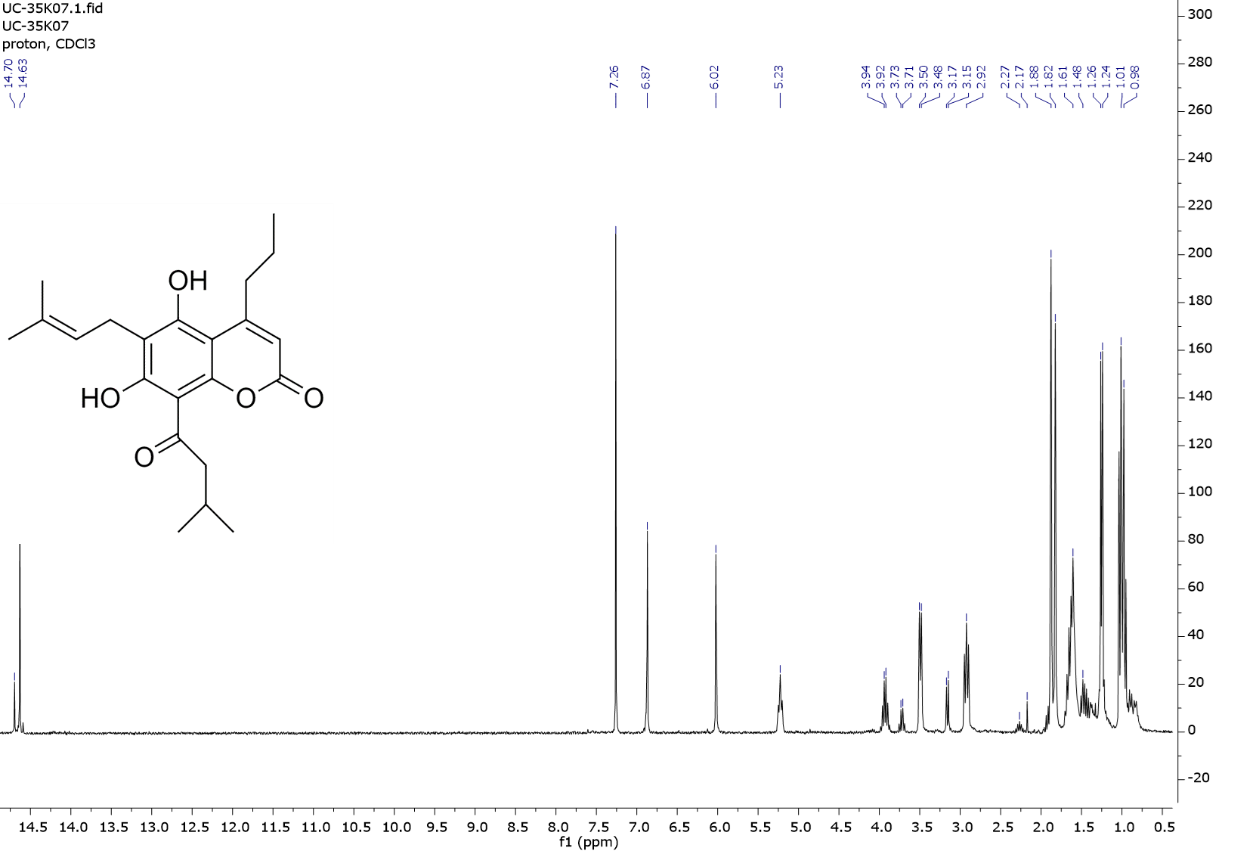


**Figure S4:** ^1^H spectrum of Mammea B/BA.


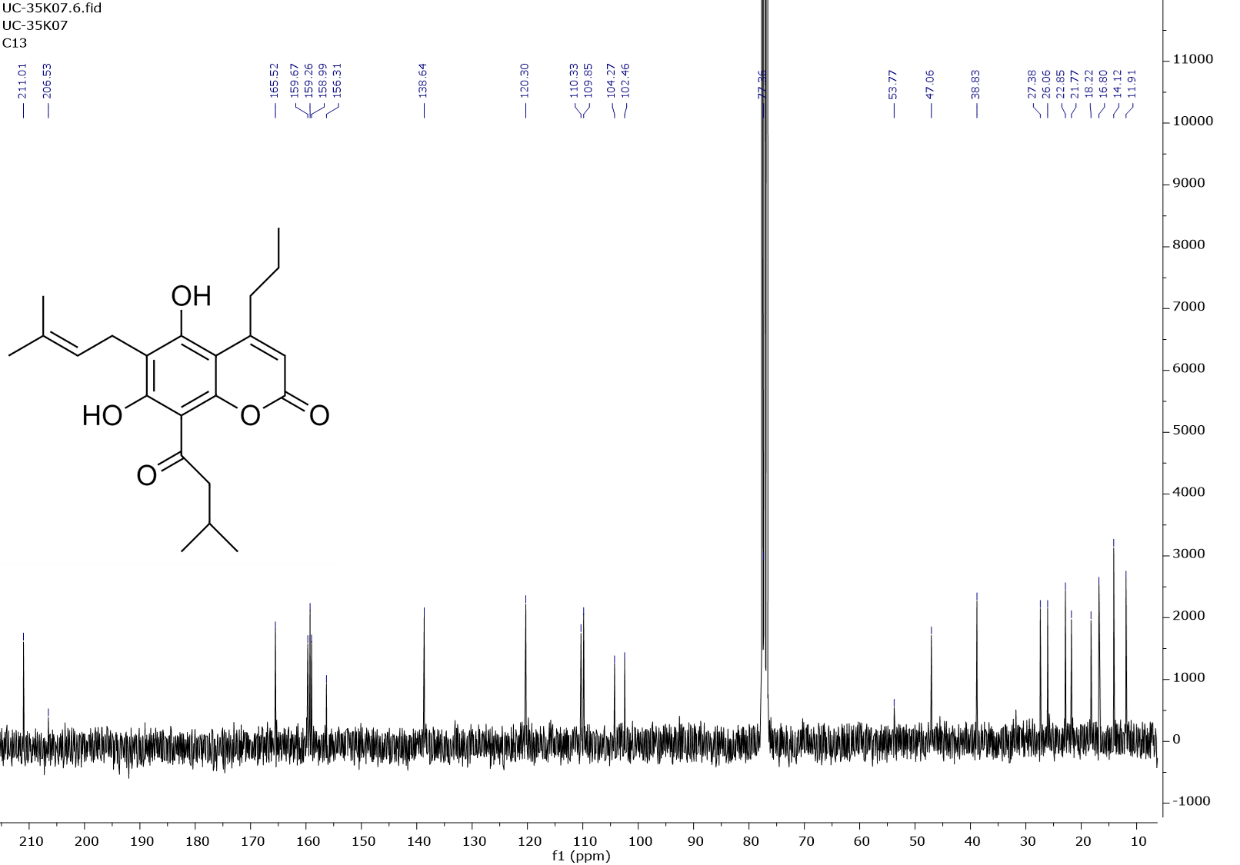


**Figure S5:** ^13^C spectrum of Mammea B/BA.


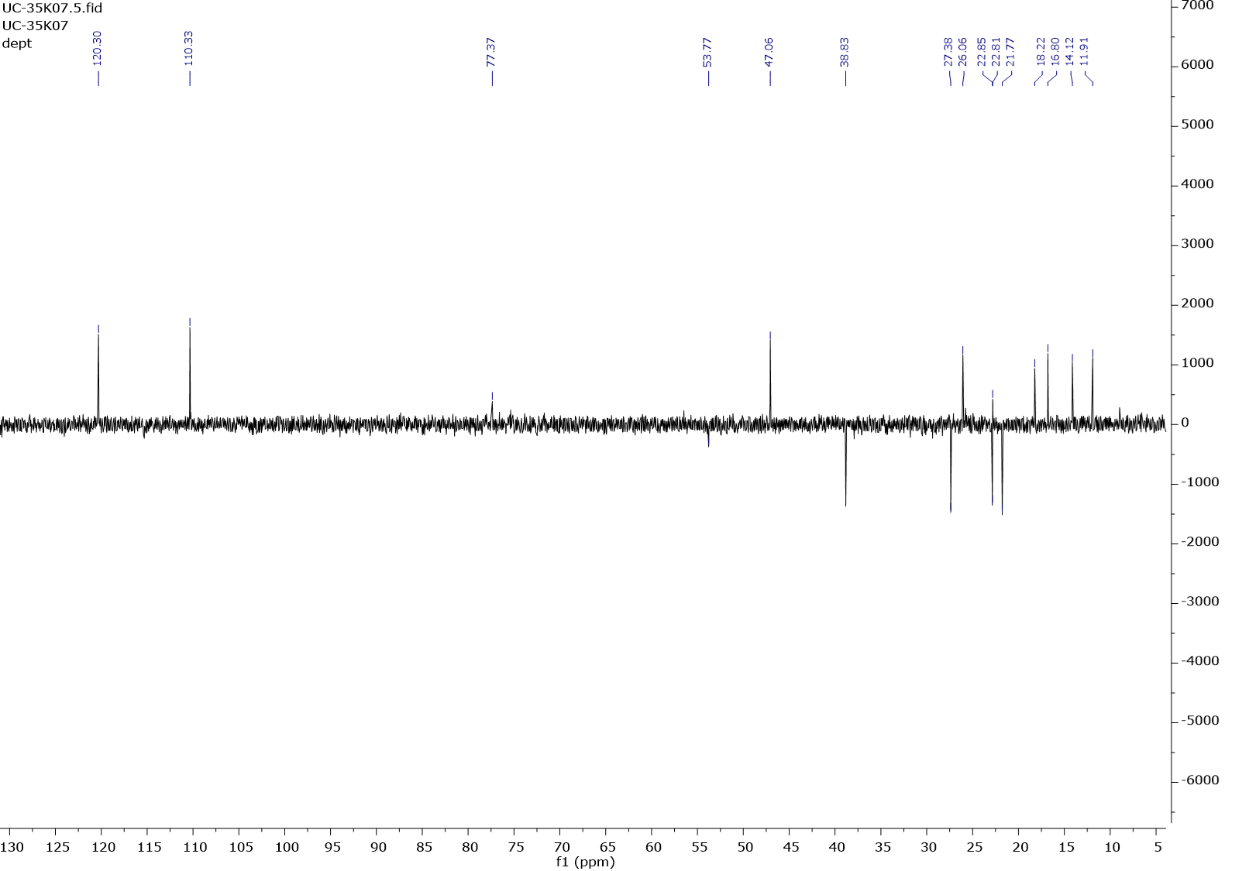


**Figure S6:** DEPT spectrum of Mammea B/BA.


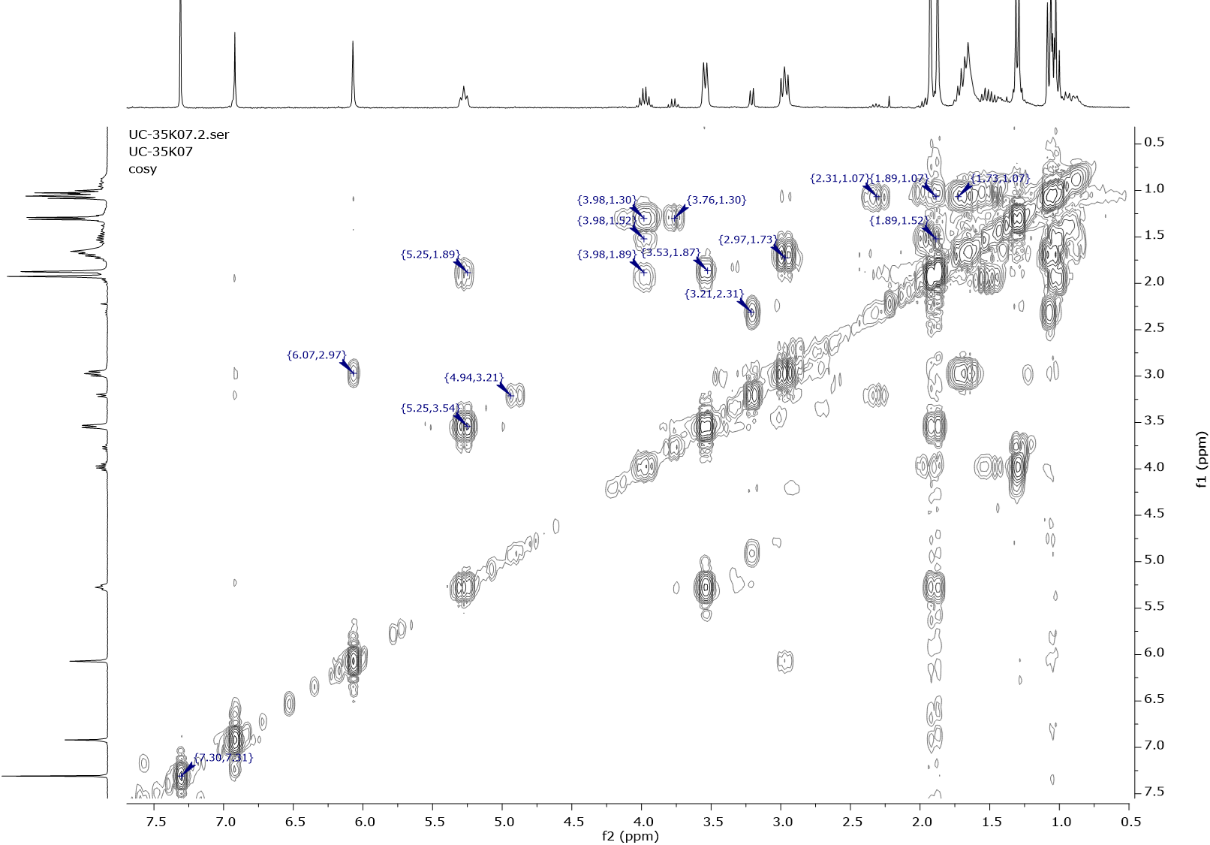


**Figure S7:** COSY spectrum of Mammea B/BA.


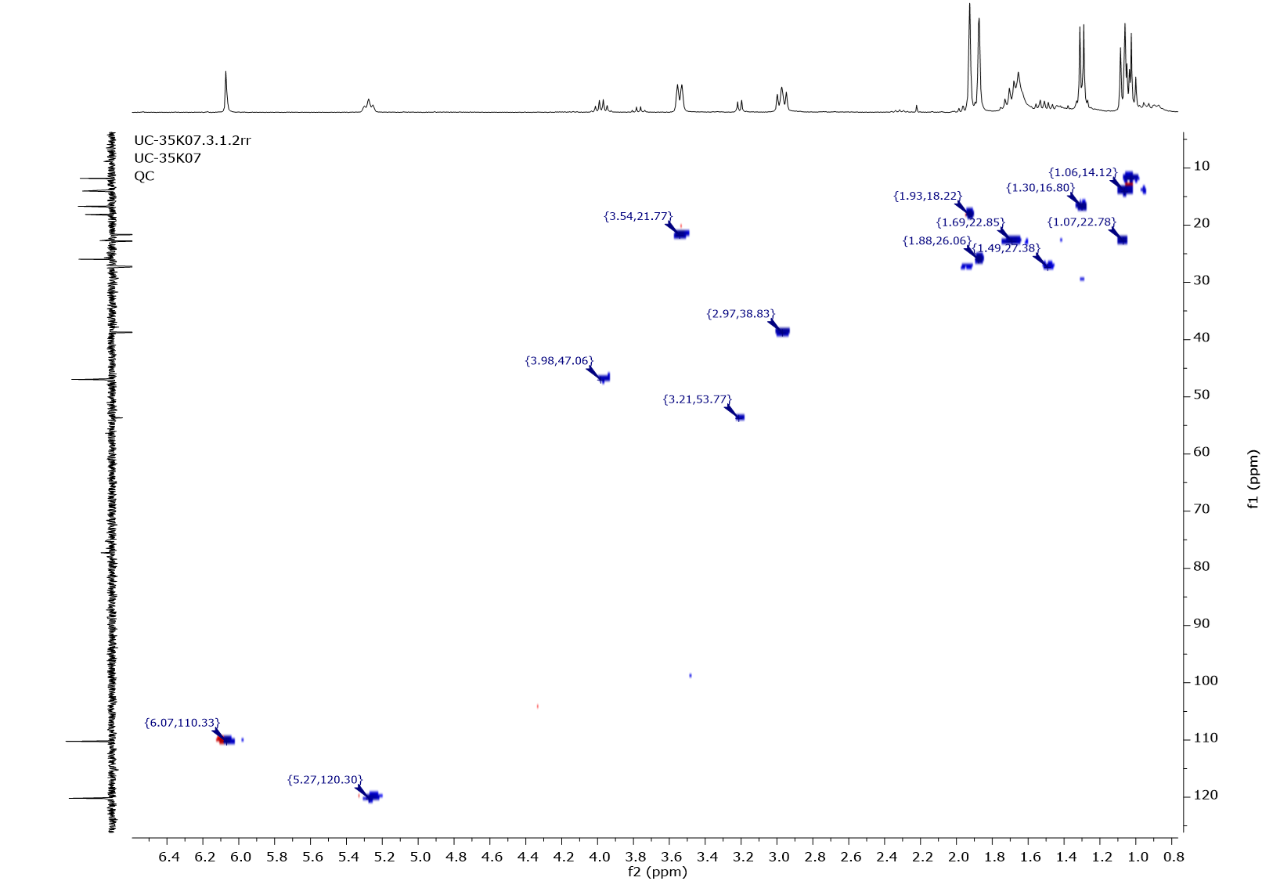


**Figure S8:** HSQC spectrum of Mammea B/BA.


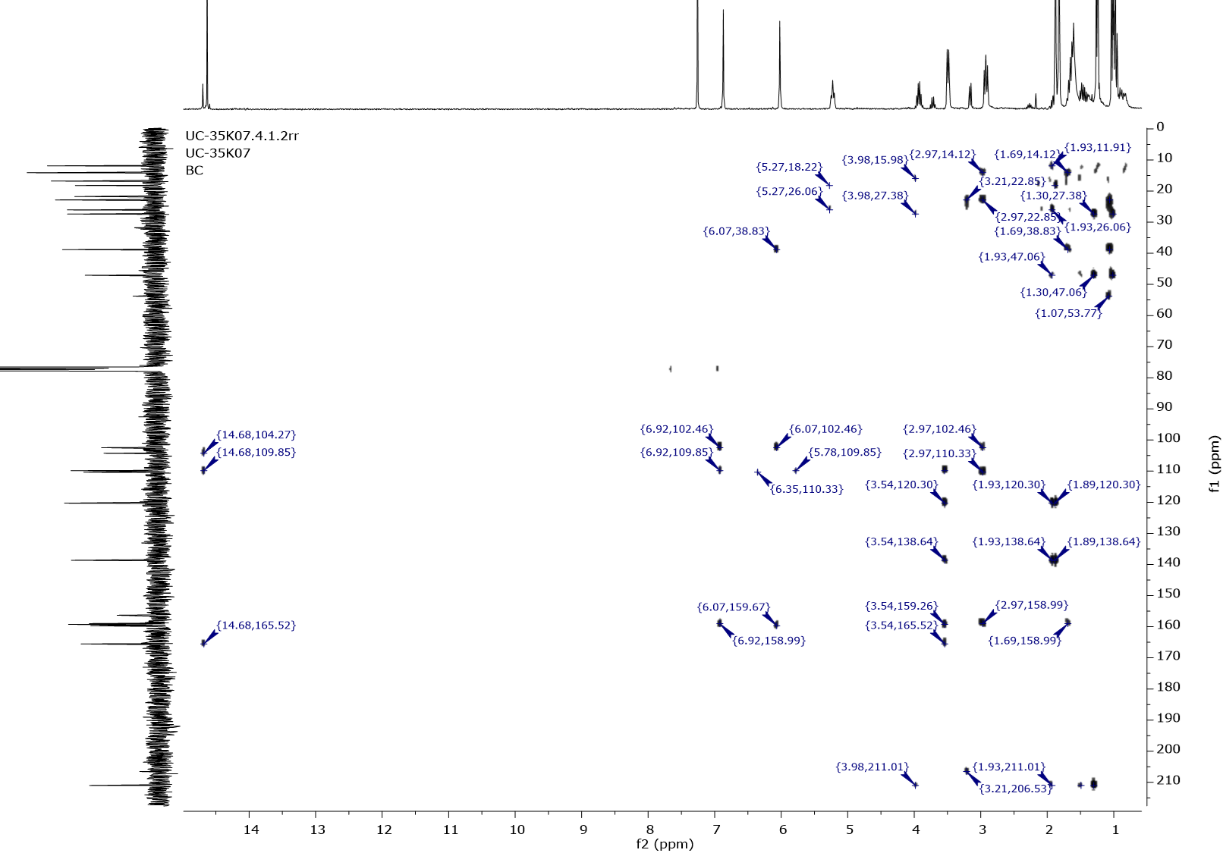


**Figure S9:** HMBC spectrum of Mammea B/BA.


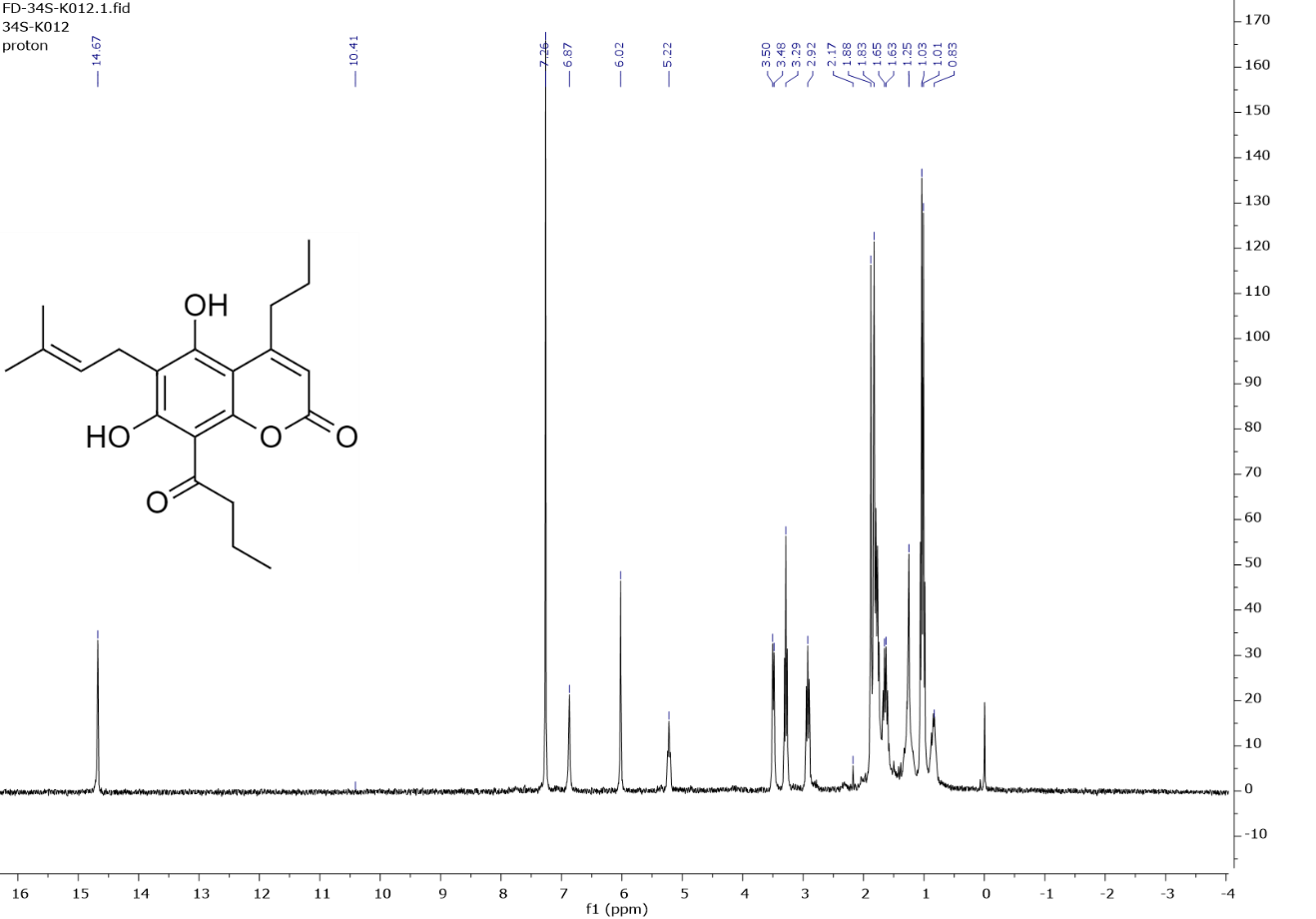


**Figure S10:** ^1^H spectrum of Mammea B/BC.


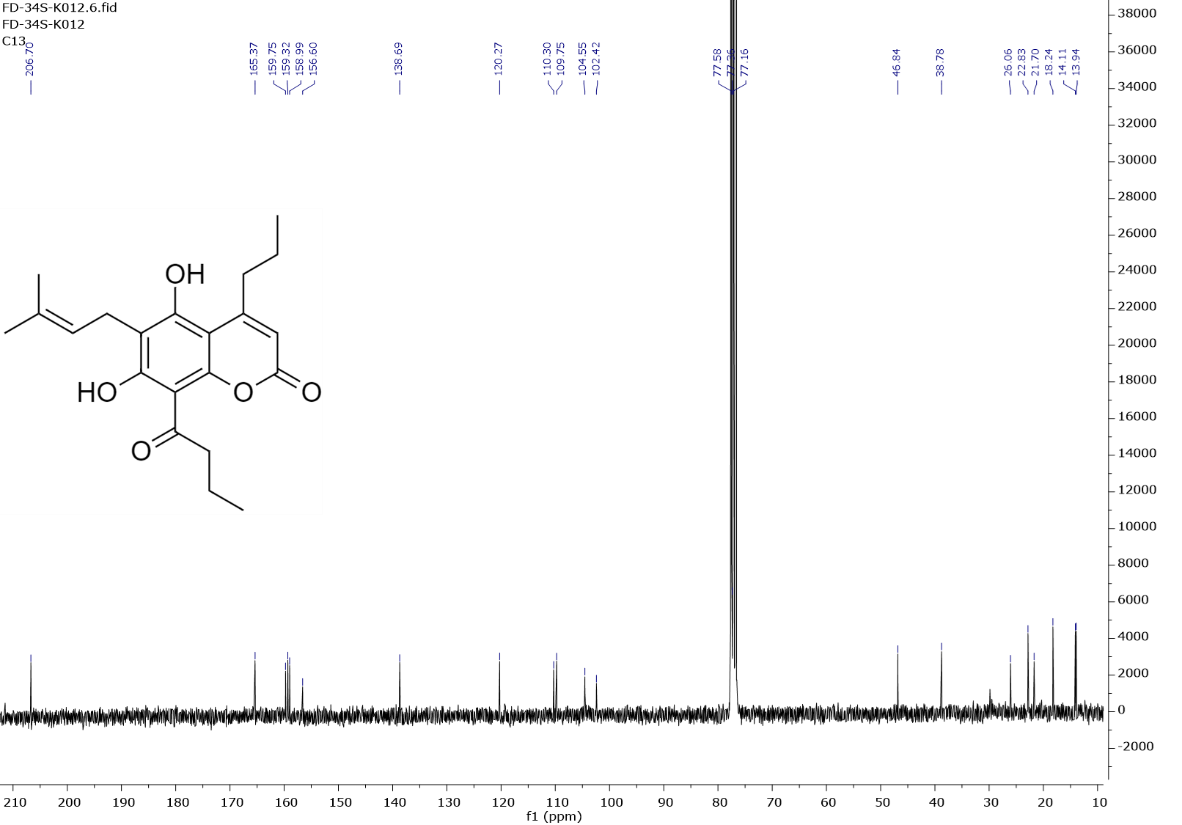


**Figure S11:** ^13^C spectrum of Mammea B/BC.


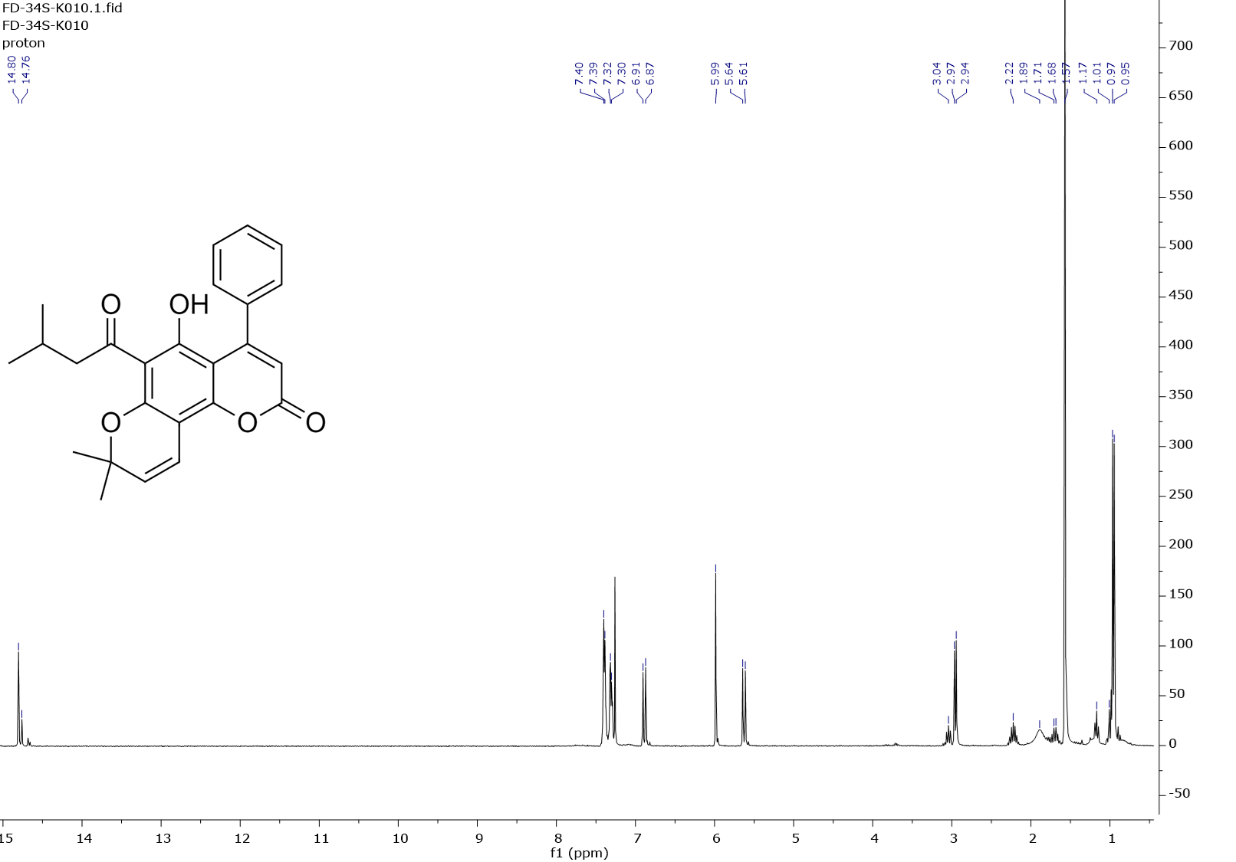


**Figure S12:** ^1^H spectrum of Mammea A/AA cycle D.


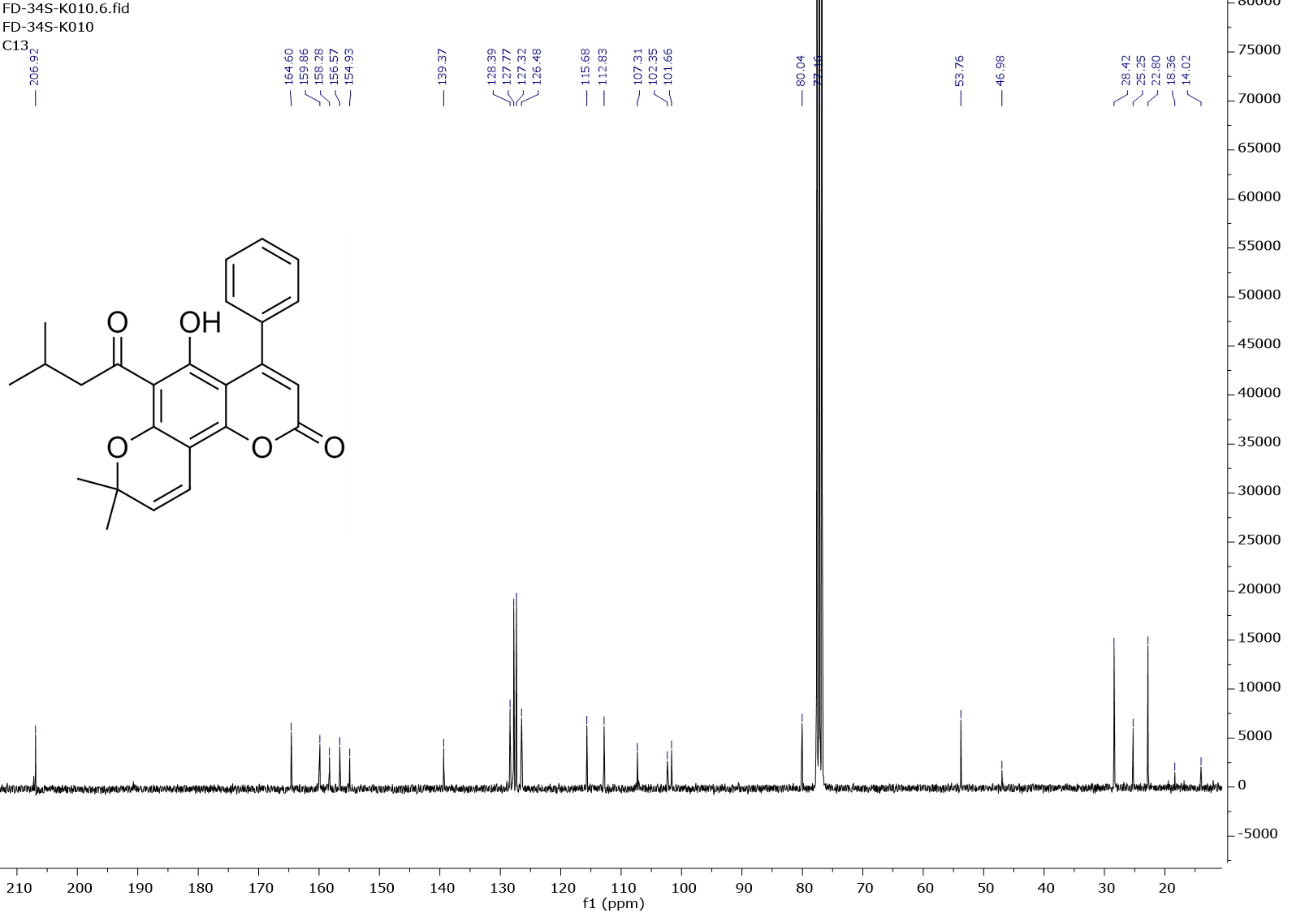


**Figure S13:** ^13^C spectrum of Mammea A/AA cycle D.


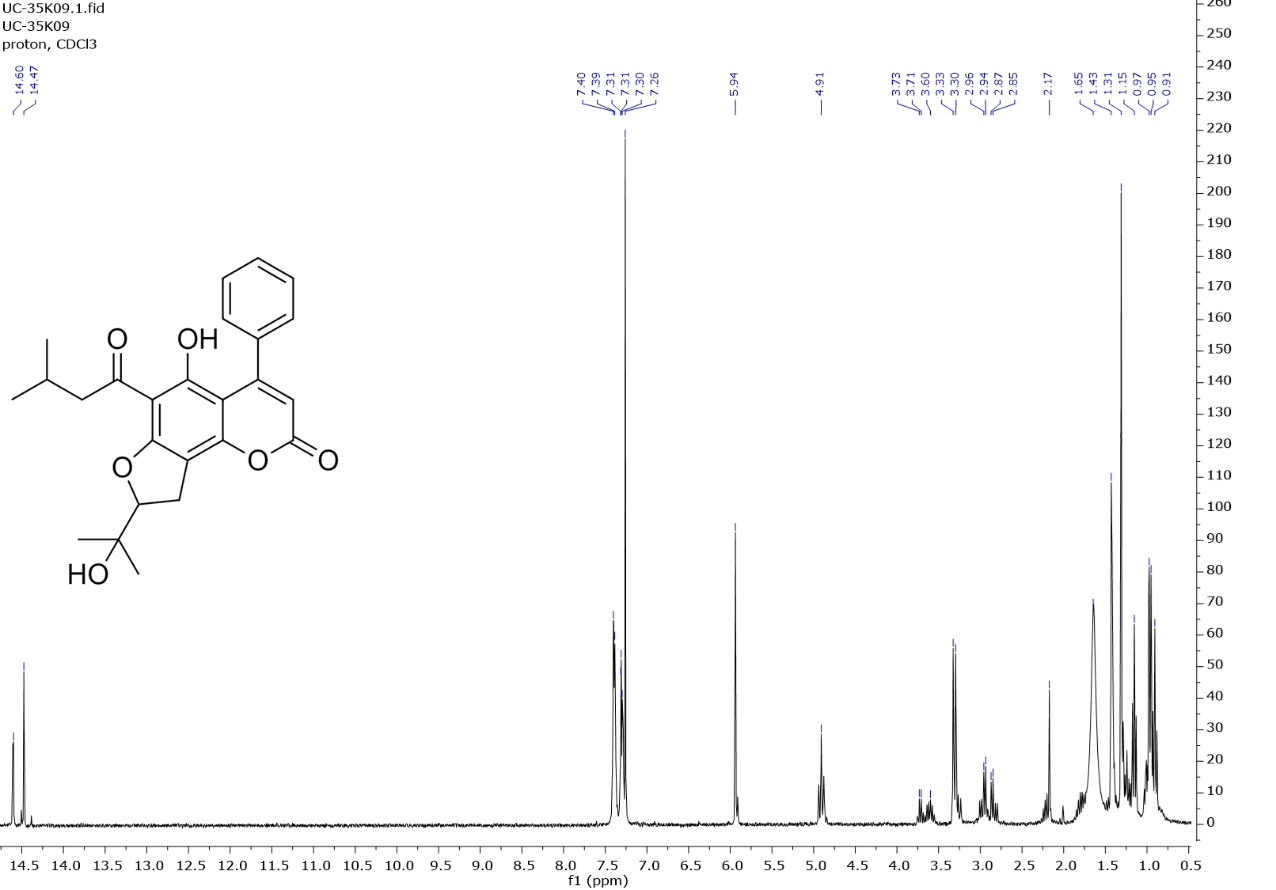


**Figure S14:** ^1^H spectrum of Mammea A/AA cycle F.


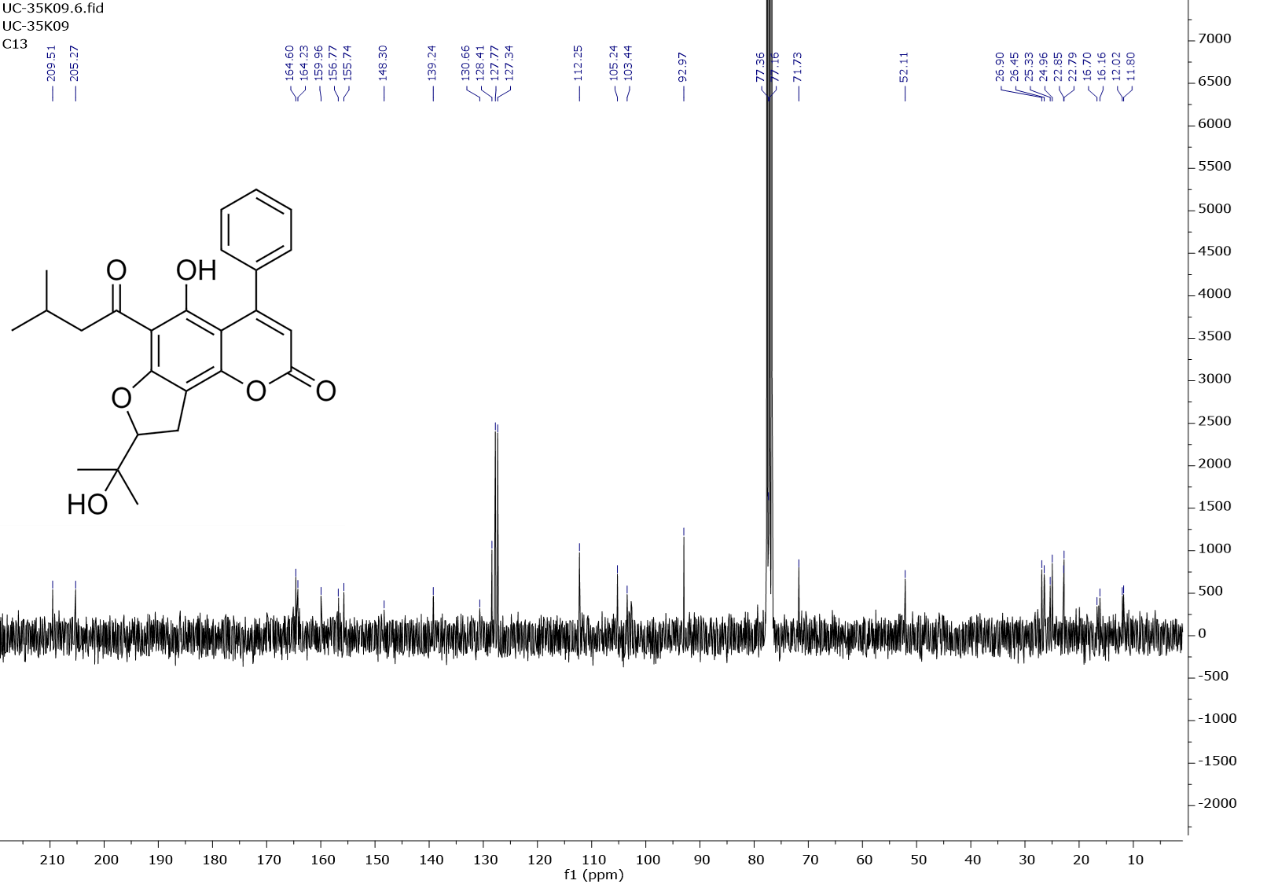


**Figure S15:** ^13^C spectrum of Mammea A/AA cycle F.


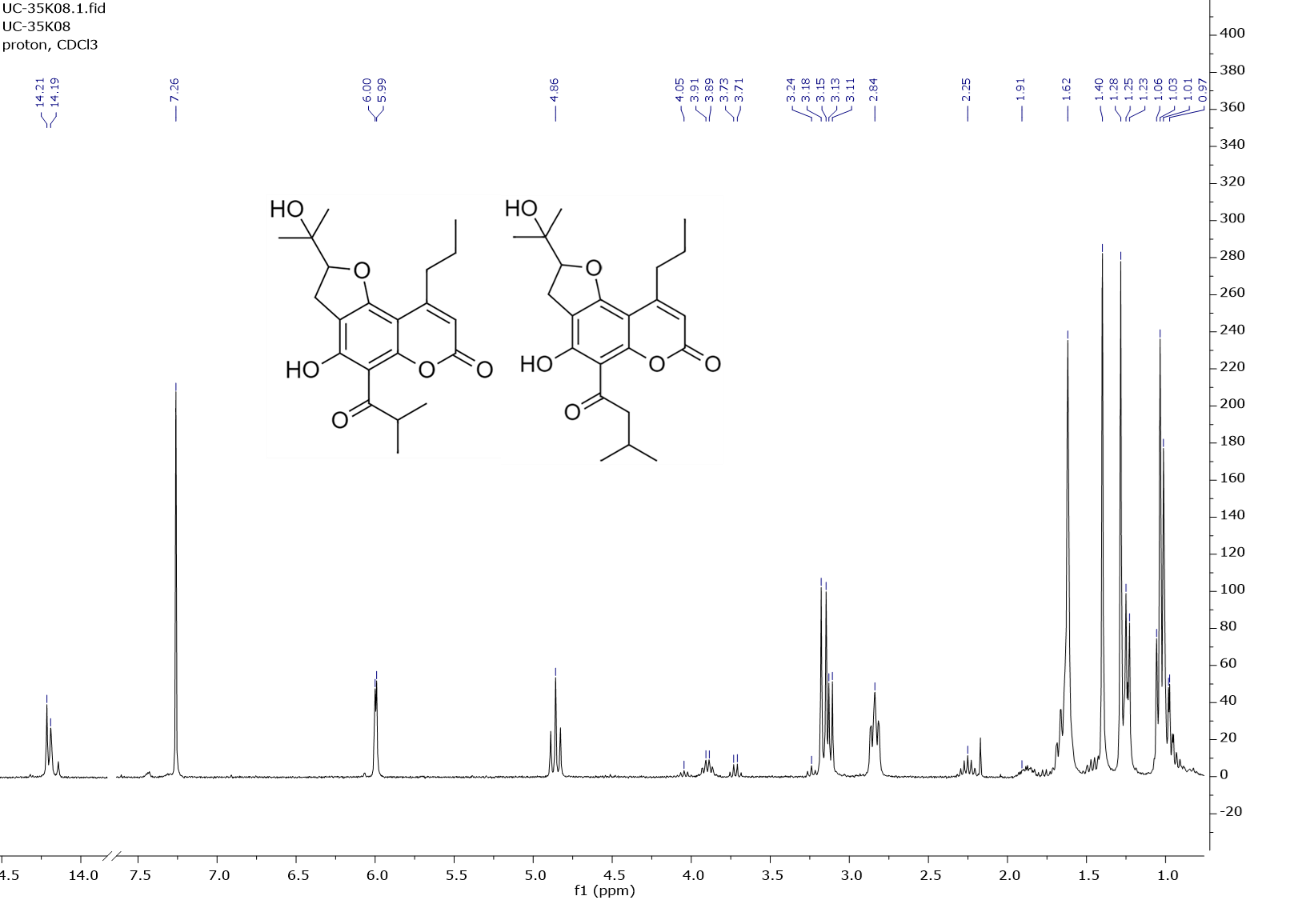


**Figure S16:** ^1^H spectrum of mixture of Mammea B/BA cycle F and Mammea B/BD cycle F.


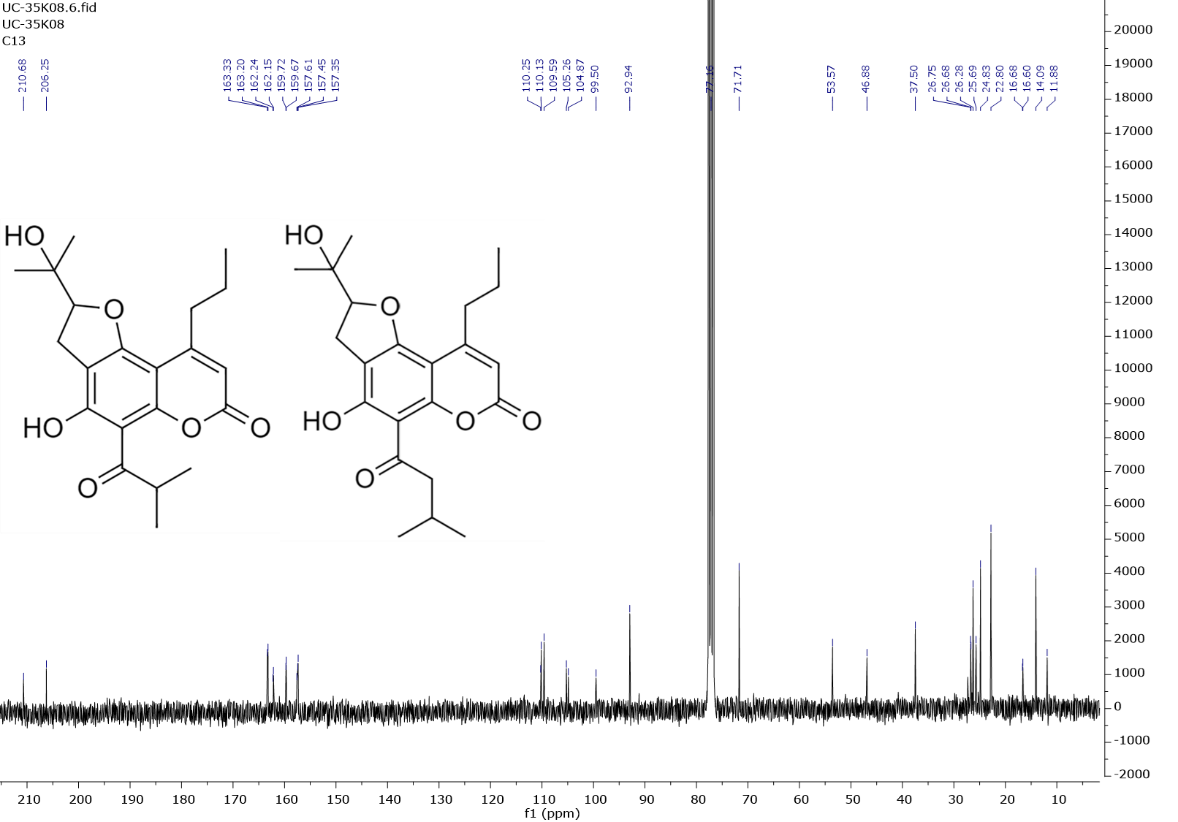


**Figure S17:** ^13^C spectrum of mixture of Mammea B/BA cycle F and Mammea B/BD cycle F.


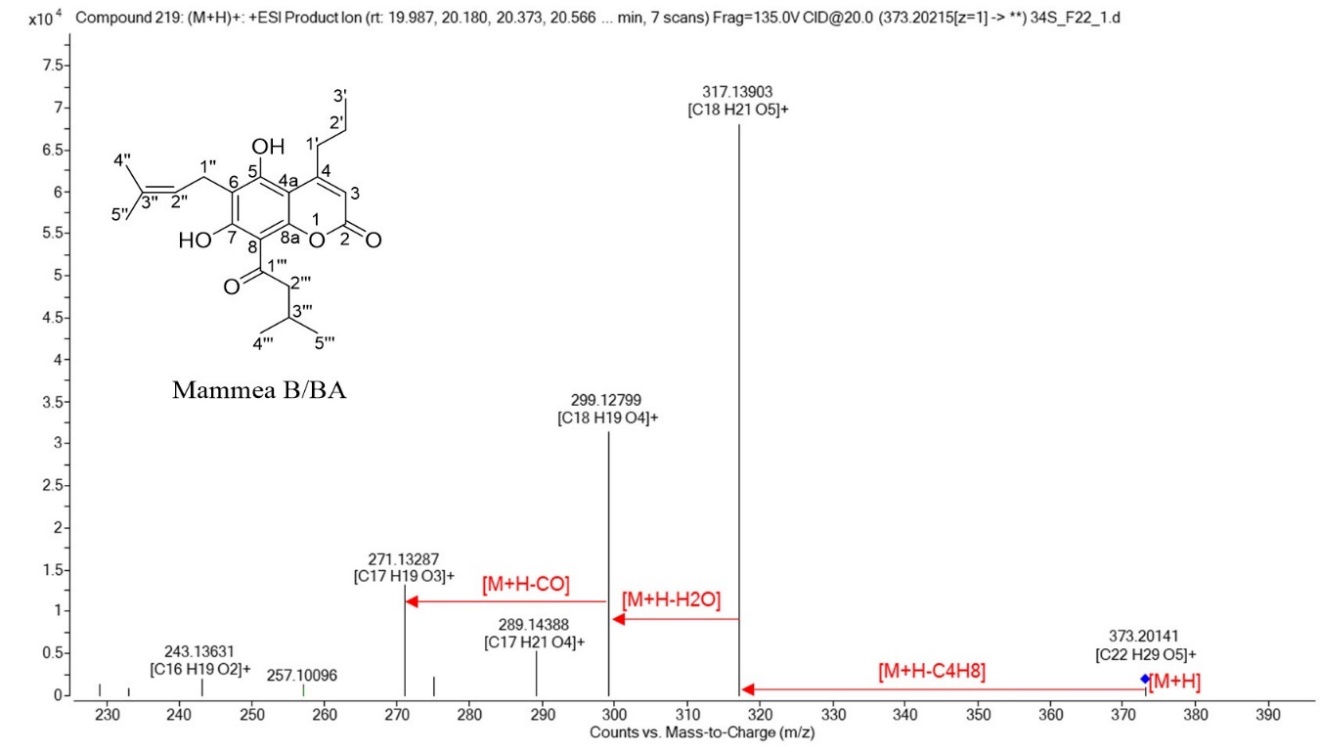


**Figure S18.** ESI-MS spectrum of Mammea B/BA corresponding to the molecular formula: C_22_H_28_O_5,_ with a protonated molecular ion at *m/z* 373,20141 [M+H] and a Retention Time (RT) of 20.34 minutes.


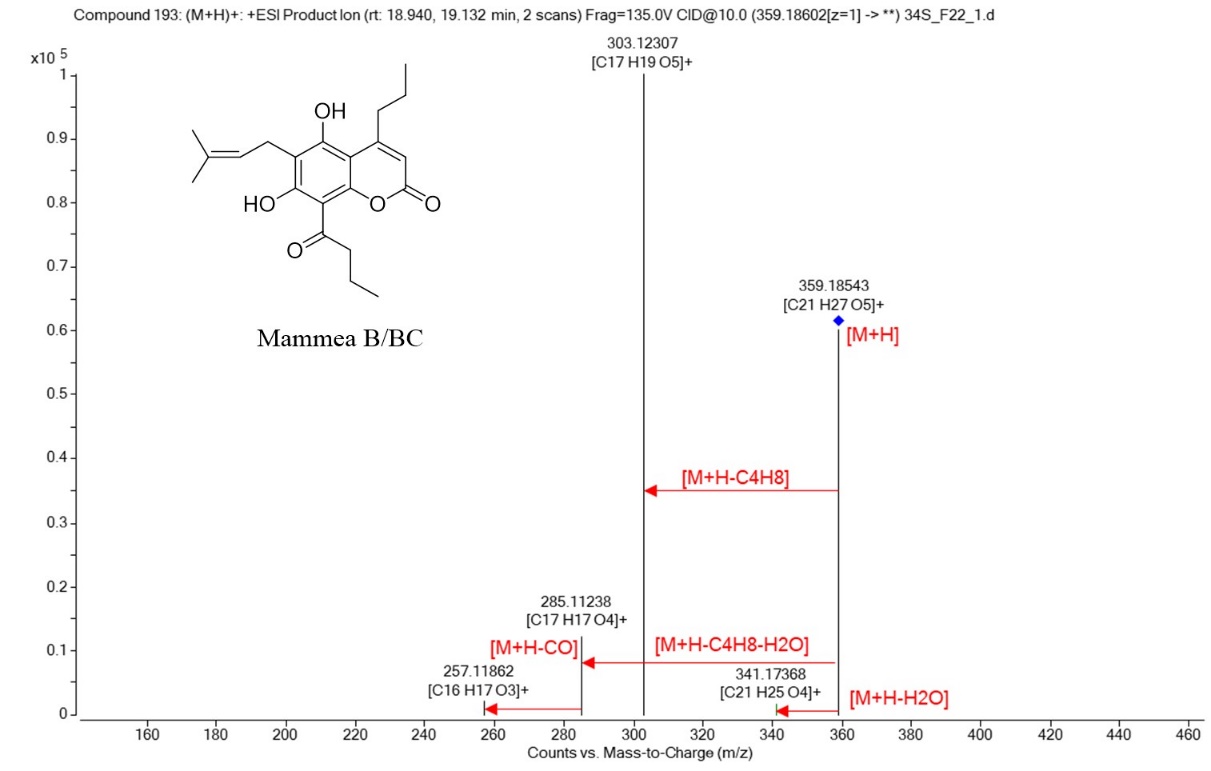


**Figure S19.** ESI-MS spectrum of Mammea B/BC corresponding to the molecular formula: C_21_H_26_O_5,_ with a protonated molecular ion at *m/z* 359,18543 [M+H] and a Retention Time (RT) of 19.06 minutes.


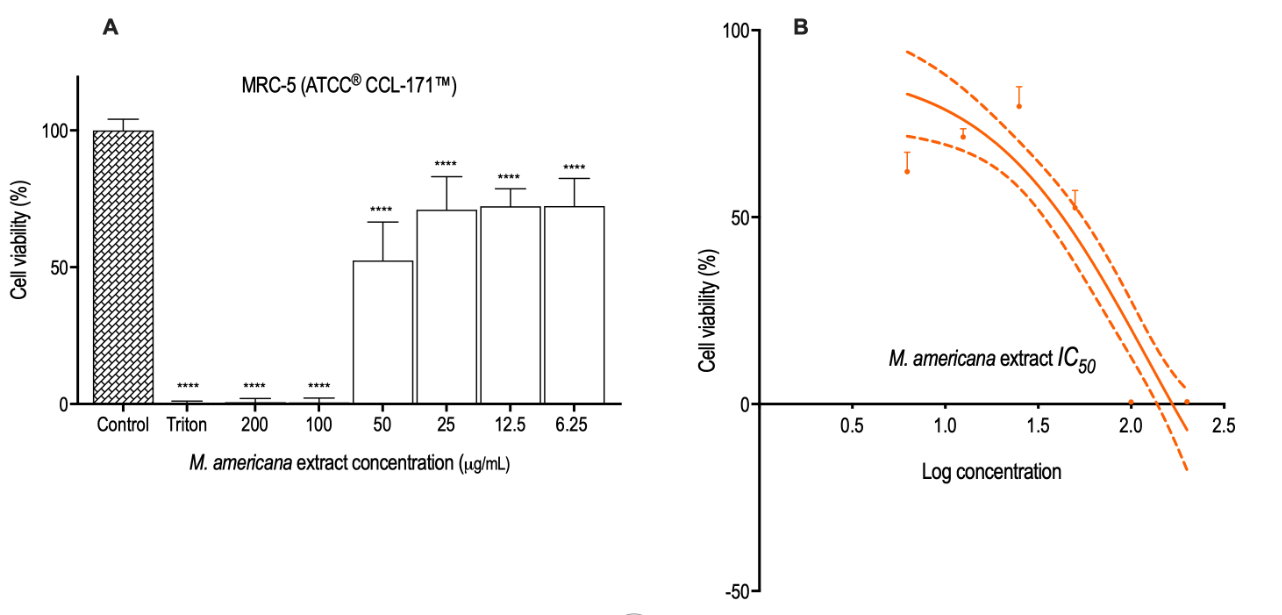


Figure S20. Cytotoxicity of *M. americana* total ethanolic extract against MRC-5 cell line. (A) Effect of different concentrations of the extract on cell growth. (b) Non-linear regression and IC_50_ calculate. Results are presented as mean± SEM of triplicate samples from three independent assays (n=9). Significant differences from growth control are indicated by ****P<0.0001.
